# Regulation of Carbon Partitioning in the Seed of the Model Legume *Medicago truncatula* and *Medicago orbicularis*: A Comparative Approach

**DOI:** 10.3389/fpls.2017.02070

**Published:** 2017-12-12

**Authors:** Youhong Song, Liang He, Xin-Ding Wang, Nathan Smith, Simon Wheeler, Manohar L. Garg, Ray J. Rose

**Affiliations:** ^1^School of Agronomy, Anhui Agricultural University, Hefei, China; ^2^School of Environmental and Life Sciences, University of Newcastle, Callaghan, NSW, Australia; ^3^School of Biomedical Sciences and Pharmacy, University of Newcastle, Callaghan, NSW, Australia

**Keywords:** GLABRA2, legumes, *Medicago orbicularis*, *Medicago truncatula*, mucilage, oil production, pectin, cell wall storage polysaccharides

## Abstract

The proportion of starch, protein and oil in legume seeds is species dependent. The model legume, *Medicago truncatula*, has predominantly oil and protein stores. To investigate the regulation of seed oil production we compared *M. truncatula* with *M. orbicularis*, which has less oil and protein. The types of protein and fatty acids are similar between the two species. Electron microscopy indicated that the size and distribution of the oil bodies in *M. orbicularis*, is consistent with reduced oil production. *M. orbicularis* has more extruded endosperm mucilage compared to *M. truncatula.* The cotyledons have a greater cell wall content, visualized as thicker cell walls. The reduced oil content in *M. orbicularis* is associated with increased expression of the *MtGLABRA2-like* (*MtGL2*) transcription factor, linked to an inverse relationship between mucilage and oil content in Arabidopsis. The expression of the pectin biosynthesis *GALACTURONOSYLTRANSFERASE* (*GAUT*) genes, is also increased in *M. orbicularis*. These increases in extruded mucilage and cell wall storage components in *M. orbicularis* are accompanied by reduced expression of transcriptional regulators of oil biosynthesis, *MtLEAFY COTYLEDON1-LIKE* (*MtL1L*), *MtABSCISIC ACID-INSENSITIVE3* (*MtABI3*), and *MtWRINKLED-like* (*MtWRI*), in *M. orbicularis*. The reduced oil in *M. orbicularis*, is consistent with increased synthesis of cell wall polysaccharides and decreased expression of master transcription factors regulating oil biosynthesis and embryo maturation. Comparative investigations between these two *Medicago* species is a useful system to investigate the regulation of oil content and carbon partitioning in legumes.

## Introduction

Legumes are the third largest family of flowering plants (Doyle and Luckow, 2003; Gepts et al., 2005) and the second most important crop family with approximately 20,000 species (Doyle and Luckow, 2003; Gepts et al., 2005), representing a significant source of protein and oil for the human diet, animal feedstock and industrial use. Legume seeds are rich in protein with globulin vicilins (7S) and legumins (11S) the most abundant (Shewry et al., 1995) but poor in sulfur-containing amino acids and contain nutritionally undesirable compounds such as protease inhibitors, which limits their nutritional value. Therefore, there has been a substantive focus on the improvement of seed protein quality (Molvig et al., 1997; Wang et al., 2003; Krishnan, 2005). There has also been a focus on sugar metabolism in non-oil storage legumes such as Vicia (Weber et al., 2005), the genetic modification of fatty acids and strategies to increase seed oil content in soybean (Clemente and Cahoon, 2009; Roesler et al., 2016). Soybean supplies nearly a third of world vegetable oil production (Graham and Vance, 2003). There is a need for more information on the cellular and genetic basis that underlies oil body formation during legume seed development (Repetto and Gallardo, 2012; Wang et al., 2012; Song et al., 2017).

Lipid storage starts from *de novo* fatty acids synthesis, followed by the synthesis of triacylglycerols (TAGs) and oil body biogenesis. The fatty acids are synthesized in the plastid, which relies on imported sucrose from maternal tissues to provide important precursors from glycolysis (Hills, 2004). TAGs are synthesized in the smooth ER by the esterification of fatty acids with the glycerol backbone, and eventually stored as individual oil bodies in the cytosol with oleosin as packaging protein. Recent studies suggest seipens are involved in early oil body assembly at specific ER domains (Cai et al., 2015). As a consequence, seed oil storage requires co-ordination of many steps. Master transcription factors identified to regulate oil storage in Arabidopsis, are LEAFY COTYLEDON1 (LEC1)/LEAFY COTYLEDON1-LIKE (L1L), LEAFY COTYLEDON2 (LEC2), FUSCA3 (FUS3) and ABSCISIC ACID INSENSITIVE3 (ABI3) (Crowe et al., 2000; Mendoza et al., 2005; Wang et al., 2007; Mu et al., 2008; Tan et al., 2011). For example, LEC1/L1L are key regulators of fatty acid synthesis, targeting multiple enzymes in the fatty acid pathway (Mu et al., 2008); FUS3 can induce *de novo* fatty acid synthesis in transgenic seedlings (Wang et al., 2007).

In addition to being used as a legume model for nodule development and the rhizobial-legume symbioses (Barker et al., 1990; Cook, 1999; Rose, 2008; Young et al., 2011) *Medicago truncatula* has been increasingly recognized as a model to study seed development and storage reserves in legumes (Gallardo et al., 2003; Wang and Grusak, 2005; Thompson et al., 2009; Wang et al., 2012; Verdier et al., 2013). We have studied embryogenesis from early development to cotyledon oil body formation (Wang et al., 2012) and profiled transcriptional regulation of early embryogenesis in preparation for seed storage (Kurdyukov et al., 2014).

An early study of Medicago seed composition (Tonnet and Snudden, 1974) showed that *M. orbicularis* seed contained approximately 3% oil compared to 9% in *M. truncatula.* As one approach to the regulation of Medicago seed oil production we carried out a comparative study, comparing *M. truncatula* with *M. orbicularis.* The comparative seed structure, cotyledon cell structure and storage composition between the two species was examined. *M. orbicularis* had more extruded endosperm mucilage compared to *M. truncatula*, thicker cotyledon cell walls and very small oil bodies. Investigations of endosperm mucilage, *GALACTURONOSYLTRANSFERASES* (*GAUTs*) involved in cell wall pectin biosynthesis and the transcriptional regulators of seed oil storage were consistent with more carbon being switched to polysaccharide biosynthesis, with less carbon available for oil biosynthesis in the *M. orbicularis* cotyledons.

## Materials and Methods

### Plant Materials

**FIGURE 1:**
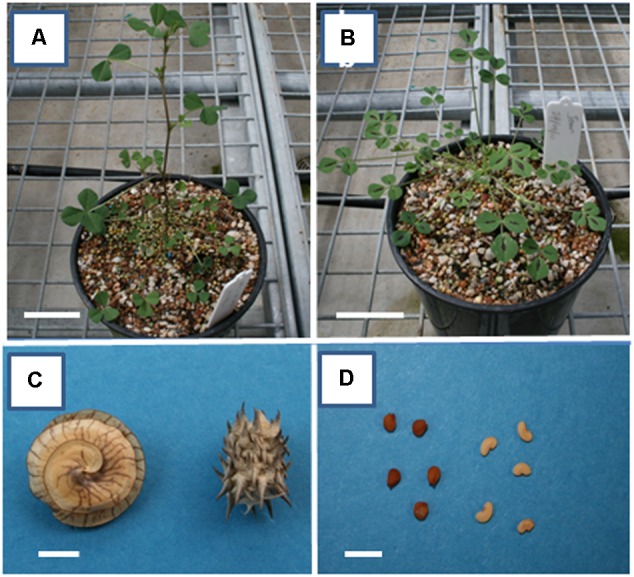
Vegetative canopy, pods and seeds of *Medicago truncatula* and *M. orbicularis.* **(A)** *M. orbicularis* and **(B)** *M. truncatula* plants 4 weeks after sowing. **(C)** Pods of *M. orbicularis* (left) and *M. truncatula* (right). **(D)** Seeds of *M. orbicularis* (left) and *M. truncatula* (right). Bars **(A,B)** 5 cm, **(C,D)** 5 mm.

*Medicago truncatula* and *M. orbicularis* (South Australia Agriculture Research and Development Institute, SARDI, accessions SA1619 and 2554, respectively) plants were glasshouse grown with a 14 h photoperiod and 23/19°C day/night temperature. The visual characteristics of plant canopy, pods and seeds for both species are shown in **Figure 1**. Plants were grown in potting mix in 15 cm pots supplemented with 5 g Osmocote Exact Standard slow release fertilizer and with automated watering. Seeds from both species were isolated from pods at 10, 14, 18, 22, and 26 days after anthesis (DAA) and stored at -80°C. Embryos from seeds of both species reach maturity at approximately the same time with similar timing for the different developmental stages (see Wang et al., 2012, for *M. truncatula* embryo development stages).

### Assays for Seed Protein, Oil and Starch

The protein assay was fully described by Wang et al. (2012). For each of three replicates, four seeds were ground in 500 μL of extraction buffer, sonicated and centrifuged at 16,000 × *g* at 4°C for 12 min, and the supernatant used to measure soluble protein by the Bradford Assay (Bradford, 1976), at a wavelength of 595 nm. The remaining insoluble protein was extracted by grinding in 500 μL of 1M NaOH and vortexing for 30 min. After centrifugation the protein in the supernatant was measured. A bovine serum albumin standard curve was used to derive protein concentrations.

Lipid extraction was based on the method of Folch et al. (1957). For each of three replicates, whole seed was homogenized with chloroform/methanol (2:1 v: v) in a final volume 20 times the volume of the sample. The mixture was agitated for 15–20 min in an orbital shaker at room temperature. The homogenate was centrifuged to recover the liquid phase. The solvent was washed with 0.2 volumes of a 0.9% NaCl solution, vortexed and then centrifuged at 3000 × *g*. The upper phase was discarded and the lower phase containing the lipid evaporated under a nitrogen stream. The fatty acid concentration was analyzed by direct transesterification of lipid using a gas chromatograph as described by Wang et al. (2012).

Total starch was extracted by following the instructions for the Abcam Starch Assay Kit (Cambridge, MA, United States). The method was slightly modified. For each of three replicates, tissue (5–10 mg) was ground using a plastic pestle in a 1.5 mL Eppendorf tube, followed by washing off of any free glucose and small oligosaccharides with 1 mL 90% ethanol at 60°C for 5 min with occasional vortexing. After removal of the supernatant, soluble starch in the pellet was extracted with 1 mL H_2_O and incubated in a boiling water bath for 60 min. After having extracted soluble starch, the water insoluble pellet was dissolved with 0.4 mL DMSO in a boiling water bath for 60 min. A standard curve was used to measure total starch concentration at OD 570 nm.

### Cell Wall Isolation

The whole seed cell wall content was determined for 6 seeds with three replicates while embryo cell wall content was determined for 10 seeds with three replicates. Dry seed was pierced using a needle or sliced with a scalpel blade and soaked in water at 60°C (in a heating block) for 10–15 min. Embryo cell wall isolation was obtained after removing the seed coat, including any adhering mucilage, after soaking in water. The details for the cell wall isolation are described in the Foster et al. (2010) protocol and outlined here. The tissue was first ground, followed by adding 1.5 ml of 70% aqueous ethanol, and the mixture vortexed. The mixture was centrifuged at 10,000 × *g* for 10 min to pellet insoluble residue and the supernatant removed. The pellet was resuspended in 1.5 mL of chloroform/methanol (1:1 v: v), vortexed, centrifuged again and the supernatant discarded. The pellet was then resuspended in 500 μL of acetone and evaporated in a fume hood. Starch was removed from the sample as described in the Foster et al. (2010) protocol. The remaining pellet was washed three times by adding 1.5 mL water. The pellet was resuspended in 500 μL of acetone and evaporated until dry. The dried material represents the lignocellulosics (cellulose, various hemicelluloses, and the polyphenol lignin) of the isolated cell walls.

### Major Seed Components

Dry weight was determined from mature seeds (three replicates of 100) after drying in an 80°C oven over 2 days. *M. truncatula* had a dry weight of 298.1 ± 25.2 mg and *M. orbicularis* 369.2 ± 3.05 mg per 100 seeds. Ten seeds with three replicates were used to determine the proportion of seed coat, embryo and extruded endosperm mucilage. The seed was first pierced, as described above, and imbibed in water at 60°C for 10 min. The seed coat, cotyledon and extruded endosperm mucilage were then separated and the dry weight determined.

### Mucilage Staining

Mucilage was isolated as in the previous section and stained by Ruthenium Red (Western et al., 2000; Verdier et al., 2013). Mucilage still adhering to the inner side of the seed coat was also stained with Ruthenium Red. The isolated swollen endosperm layer was prepared for Ruthenium Red staining by a shorter incubation at 60°C and removal of seed coat and embryo.

### Histology of Thin Sections

Dry seed was imbibed at 4°C for 5 days then fixed and embedded in LR White resin as previously described (Rose et al., 2006; Wang et al., 2012). Sections of 1 μm were stained in Toluidine Blue and Azur II and viewed and photographed with a Zeiss Axiophot microscope.

### Transmission Electron Microscopy

The dry seed was first imbibed in water for 5 days at 4°C and then fixed using 4% (w:v) glutaraldehyde and 1.5% (w:v) paraformaldehyde in 25 mM phosphate buffer (pH 7) at 4°C for 4 h, with at least one change of buffer. Tissue was transferred to phosphate buffer, washed once and incubated overnight at 4°C. The tissue was then postfixed in 1% aqueous osmium tetroxide overnight at 4°C and washed twice with distilled water, then chilled to 4°C. Dehydration steps and infiltration into LR White resin has been described (Wang et al., 2012). Ultrathin sections were cut with a diamond knife and transferred to 200-mesh grids. The sections were stained with uranyl acetate and lead citrate and viewed using a JEOL JEM 1200-EXII electron microscope (JEOL Ltd., Tokyo, Japan).

### Identification of Genes from the *M. truncatula* Genome Sequence

Homology-based methods using genes from other plant families have been successfully employed to identify specific classes of legume transcription factors (Udvardi et al., 2007). The phylogenetic analyses were based on protein or peptide sequences (e.g., Supplementary Figure S1a) from Arabidopsis, *M. truncatula* and soybean from phytozome v9.1 (*M. truncatula* genomic sequence 3.5). Candidate genes were checked against information from the NCBI database. Supplementary Table S1 provides information about the genes using the improved genome release *M. truncatula* version Mt4.0 (Tang et al., 2014).

### Sequencing of Candidate Genes for *M. orbicularis*

Primers for sequencing were designed based on *M. truncatula* genes using Primer 3. The cDNA template from *M. orbicularis* was used to amplify the fragment, and a single band with similar size to *M. truncatula* was produced. The product was purified with Promega cleanup (Promega, Sydney, NSW, Australia) prior to sequencing at the Australian Genome Research Facility, Brisbane (AGRF^1^). Alignment of sequences between *M. truncatula* and *M. orbicularis* showed high similarity with only a few nucleotides varying (e.g., Supplementary Figure S1b). The same primers for gene expression comparisons between the two species could be used.

### RNA Extraction, cDNA Synthesis and Real Time Quantitative PCR

RNA was extracted from young developing seed (before 18 DAA) using the RNAqueous-4PCR kit (Ambion, Sydney, NSW, Australia) and from older developing seed (after 18 DAA) using a phenol-chloroform method (Li and Trick, 2005) due to high phenolics or carbohydrate in these seeds. Genomic DNA was removed using DNase treatment. cDNA synthesis was performed with a SuperScript III first-strand synthesis system (Invitrogen, Melbourne, VIC, Australia) using 1 μg of total RNA and oligo(dT) primers.

The cDNA was diluted 1:25 for quantitative PCR (qPCR) reactions. All qPCR reactions were prepared using a CAS1200 robot (Qiagen, Melbourne, VIC, Australia) and run on a Rotor-Gene Q (Qiagen). Primers were designed using Primer3 (Supplementary Table S2). Reactions were performed with a 15 μL sample volume using Platinum Taq PCR polymerase and 2 μM SYTO9 fluorescent dye (Invitrogen). PCR cycling conditions were 94°C for 2 min, followed by 40 cycles of 94°C for 15 s, 60°C for 30 s and 72°C for 30 s. Dissociation analysis was performed in each run to verify the amplification of a specific product. Gene expression was normalized to the expression of GAPDH. GAPDH has been used as a suitable reference gene for *M. truncatula* embryogenesis and seed development investigations (Wang et al., 2012; Kurdyukov et al., 2014) and verified experimentally by other types of gene analysis (Nolan et al., 2003, 2009), microarray and RT-qPCR studies (Mantiri et al., 2008). Using geNORM software, Verdier et al. (2008) have also shown GAPDH to be a suitable reference gene.

PCR efficiency of each run was calculated using the LinRegPCR program of Ramakers et al. (2003). Relative expression was calculated using the Pfaffl (2001) method. Gene expression was relative to 10 days after anthesis. Results shown are means ± SE of three biological repeats.

### Proteomics

Protein was extracted using a Plant Total Protein Extraction Kit (Sigma–Aldrich). Seeds from *M. truncatula* and *M. orbicularis* 12, 14, 18, and 22 DAA were used to profile protein composition. The protein was quantified using the Bradford (1976) assay. Protein was precipitated using methanol/chloroform, digested with sequencing grade trypsin (Promega) and protein digestion was stopped with a final concentration of 0.1% trifluoroacetic acid. The samples were then centrifuged twice at 16,000 × g for 30 min to pellet undigested protein. The supernatant was transferred to vials for nanoflow reversed phase liquid chromatography tandem mass spectrometry (LC-MS/MS) analysis.

Liquid chromatography tandem mass spectrometry analysis was similar to Baker et al. (2010) with the following deviations: peptides were sequenced by nanoflow reverse phase Liquid Chromatography (nanoACQUITY Ultra Performance LC, Waters, Milford, MA, United States) coupled directly to an ESI Q-ToF Mass Spectrometer (MicrOtof QII, Bruker GmbH, Preston, VIC, Australia) operating in positive ion MS/MS mode. Peptides were loaded at 5 μl/min onto a C18 trap column (Waters, nanoACQUITY UPLC Symmetry C18 Trap, 5 μm, 180 μm × 20 mm) for desalting and pre-concentration. Peptide separation was then performed at 300 nl/min over an nanoACQUITY UPLC BEH C18 Column, 1.7 μm, 75 μm × 150 mm (Waters), using a linear gradient of 0-60% buffer (84% ACN, 0.1% formic acid) over 200 min. The peptides were eluted directly into the nanoflow ESI ion source of the MS system for MS/MS analysis. The Q-ToF system was set to perform MS/MS on the top 5 ions present in each MS scan with an ion exclusion time of 30 s. Raw MS Files were converted into MASCOT Generic Format using Data Analysis 4.0 and imported into ProteinScape 2.1 platform (both Bruker, Bremen, Germany) for database searching. Searches were performed against the annotated *M. truncatula* protein database (in FASTA format; IMGAG 3.5 release was obtained using the Phytozome site^2^) and SwissProt (Green Plant) databases using an in-house licensed MASCOT server (version 2.3, Matrix Science). The number of allowed trypsin missed cleavages was set to 2. Deamidation of asparagine and glutamine, oxidation of methionine and phosphorylation of serine, threonine and tyrosine were set as variable modifications. The parent peptide ion tolerance was set to 1.2 Da with MS/MS fragment ion tolerance set to 0.7 Da. Peptide thresholds were set requiring False Positive Rate less than 0.05% with a low stringency MASCOT score greater than 35. Those spectra meeting these criteria were validated by manual inspection to ensure accurate y- and b-ion detection with overlapping sequence coverage. Each *M. truncatula* protein returning a positive peptide ID (significance value of *p* < 0.05) was interrogated against the non-redundant protein database at the NCBI^3^ and at Phytozome^4^ using BLAST-P to confirm protein identity and to classify the protein’s function.

### Statistical Tests

Standard errors are indicated and Student’s two-tailed *t*-test was used for experimental comparisons. A single asterisk (^∗^) indicates that the difference is statistically significant at *P* < 0.05 while double asterisks (^∗∗^) indicate that the difference is statistically significant at *P* < 0.01. Treatments with different letters are significantly different at the 0.05 probability level, with the statistical analysis performed by comparing means in the SPSS 18.0 statistical package.

## Results

In our initial analyses we characterized the seed composition of the *M. truncatula* and *M. orbicularis* accessions (**Figure 1**) used in this study.

### Seed Oil and Fatty Acid Content

**FIGURE 2:**
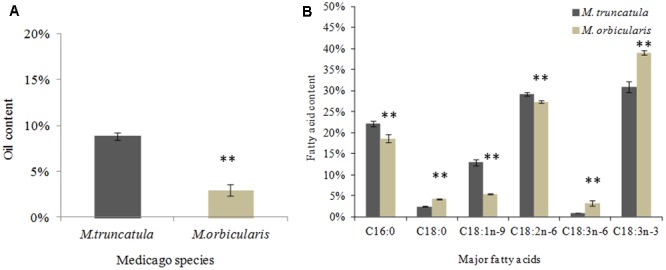
Oil and fatty acid content of *M. truncatula* and *M. orbicularis* as a percentage of seed dry weight. **(A)** Oil and **(B)** Fatty acid content. SE indicated and ^∗∗^*p* < 0.01 (*n* = 3).

Seed oil content (oil weight/seed dry weight) and fatty acid composition of seeds in *M. truncatula* and *M. orbicularis* are shown in **Figures 2A,B**. The mature seed accumulated approximately 9% oil in *M. truncatula* and 3% in *M. orbicularis* **Figure 2A**. The most abundant fatty acids in **Figure 2B** for both species were C16:0 (palmitic acid), C18 1n-9 (oleic acid), C18:2n-6 (linoleic acid), and C18:3n-3 (alpha-linolenic acid, an omega-3 fatty acid). *M. orbicularis* was lower in C16:0, C18:1n-9, and C18:2n-6. Alpha-linolenic acid (C18:3n-3) was higher in *M. orbicularis* than in *M. truncatula*. Two minor fatty acids, C18:0 (stearic acid) and C18:3n-6 (gamma-linolenic acid, an omega-6 fatty acid), were also higher in *M. orbicularis*.

### Seed Protein Content

**FIGURE 3:**
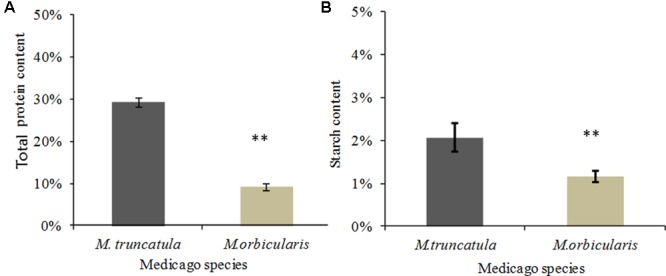
Protein and starch content of *M. truncatula* and *M. orbicularis* as a percentage of seed dry weight. **(A)** Total protein content. **(B)** Starch content. SE indicated and ^∗∗^*p* < 0.01 (*n* = 3).

The seed storage protein as a proportion of seed dry weight was compared between *M. truncatula* and *M. orbicularis* (**Figure 3A**). *M. truncatula* seed accumulated approximately 30% storage protein while *M. orbicularis* seed accumulated only 10% storage protein. We subsequently carried out proteomics using LC-MS/MS to identify the major storage proteins in both species. These are shown in **Table 1** and Supplementary Table S3. The storage proteins identified in both Medicagos were very similar at both 18 and 22 days. The proteins identified were synthesized by multiple genes. As is known in *M. truncatula* the major storage proteins are vicilins/convicilins, legumins, and conglutins (Gallardo et al., 2003). The gene redundancy presumably reflects the importance of the storage proteins for survival in the next generation. To check that the *M. truncatula* and *M. orbicularis* storage protein profiles showed similar trends during development, data were obtained at 12, 14, 18, and 22 days after anthesis (DAA). The data again are consistent with similar protein profiles between the two species (**Table 1** and Supplementary Table S3). In *M. truncatula* only the convicilin proteins are present at 12 DAA when SSP (seed storage protein) gene expression is initiated and the cotyledons have just started to develop (Supplementary Table S3 and Wang et al., 2012). A wider spectrum of SSPs are present at 14 DAA (Supplementary Table S3). In the case of *M. orbicularis* convicilin proteins are present at 14 DAA plus other SSPs, reflecting a slight difference (approximately 1 day) in cotyledon development timing. It was of interest that lipoxygenase is a major seed protein. This seed protein is important in germination to lyse oil bodies and is bound to the oil bodies at that time (Feussner et al., 2001). Proteome studies indicate it can also be bound to seed oil bodies (Jolivet et al., 2013).

**Table 1:** Major proteins of *Medicago truncatula* and *M. orbicularis*, days after anthesis (DAA) indicated.

Gene identifier (Supplementary Table S3)	Protein	*M.* truncatula 18 days	*M.* truncatula 22 days	*M.* orbicularis 18 days	*M.* orbicularis 22 days
Medtr7g079820	Convicilin^∗^	+	+	+	+
Medtr7g079780	Convicilin^∗^	+	+	+	+
Medtr7g079740	Convicilin^∗^	+	+	–	–
Medtr7g079730	Convicilin^∗^	+	+	+	+
Medtr7g079770	Provicilin	+	+	+	+
VCLC_PEA	Vicilin Precursor	+	+	+	+
Medtr5g019780	Vicilin-like antimicrobial	+	+	+	+
Medtr1g072630	Legumin B	+	+	+	+
Medtr1g072610.2	Legumin B	+	+	+	+
Medtr1g072600.1	Legumin B	+	+	+	+
LEG_CICAR	Legumin	+	+	+	+
LEG_PEA	Legumin A	+	+	–	+
LEG_JPEA	Legumin J	–	–	+	+
LEGU_CANEN	Legumin	–	–	+	–
Medtr2g083160	Conglutin	+	+	+	+
Medtr2g099570	Seed lipoxygenase	+	+	+	+
Medtr2g099560	Lipoxygenase	+	+	+	+

*The more detailed data relating to individual genes can be found in Supplementary Table S3. ^∗^Also annotated as vicilin (LegumeIP Database, Li et al., 2012).*

### Seed Starch Content

The content of total seed starch in *M. truncatula* and *M. orbicularis* is shown in **Figure 3B**. Seeds from both species had very low total starch content (with only 1–2% of seed dry weight) compared to protein or oil content. *M. truncatula* seed accumulated higher total starch than *M. orbicularis* mainly because of higher insoluble starch in *M. truncatula*. It was clear that the starch content in *M. orbicularis* was not increased despite there being less oil and protein.

### Ultrastructure of Mature Cotyledons in *M. truncatula* and *M. orbicularis*

**FIGURE 4:**
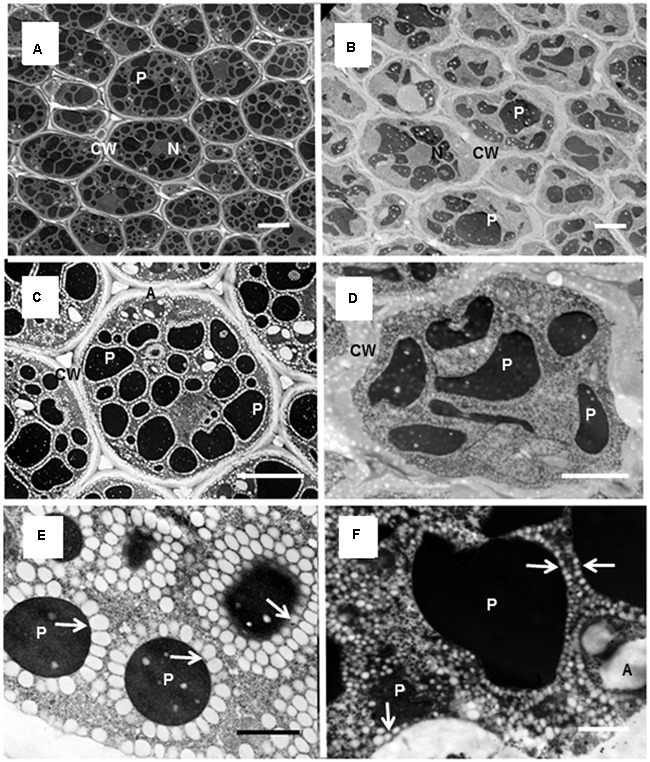
Electron micrographs of cells of *M. truncatula* and *M. orbicularis* cotyledons. View of multiple cells of *M. truncatula* **(A)** and *M. orbicularis* **(B)**; single cells of *M. truncatula* **(C)** and *M. orbicularis* **(D)** and subcellular structure of *M. truncatula* **(E)** and *M. orbicularis* **(F)**. Amyloplasts (A), cell walls (CW), nucleus (N), oil bodies (arrows), protein bodies (P). Bars **(A–D)** 5 μm, **(E,F)** 1 μm.

To obtain more information on the storage components, transmission electron micrographs of cotyledons of mature seeds in *M. truncatula* and *M. orbicularis* were obtained (**Figure 4**). The cotyledons are the major site of storage components. The cotyledon cells of *M. truncatula* are shown in **Figures 4A,C** and of *M. orbicularis* in **Figures 4B,D**. At higher magnification oil bodies could be readily visualized as white spherical to ovoid structures in both species (**Figures 4E,F**), but were very much smaller in *M. orbicularis*. As previously observed, the *M. truncatula* oil bodies surrounded the protein bodies and also aligned along the plasma membrane. The *M. orbicularis* oil bodies also surrounded the protein bodies but not in concentric layers and not as uniformly. The small *M. orbicularis* oil bodies were also randomly spread throughout the cytoplasm.

**FIGURE 5:**
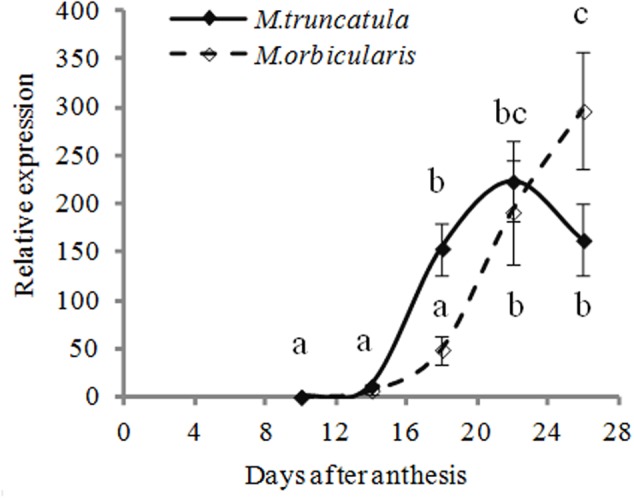
Relative expression (relative to 10 DAA) of *Mt-OLEOSIN2* (Medtr3g109190) during *M. truncatula* and *M. orbicularis* seed development. SE indicated and treatments with different letters are significantly different at the 0.05 probability level (*n* = 3).

We considered whether oleosin might be limiting in *M. orbicularis* but we couldn’t obtain any evidence for that, consistent with the small size of the oil bodies. The transcription of a highly expressed oleosin gene (Medtr3g109190) we had previously identified as *MtOLEOSIN2* (Wang et al., 2012) and present in both *M. truncatula* and *M. orbicularis* (Supplementary Table S3) was examined. There was clearly greater early expression in *M. truncatula* than in *M. orbicularis*. Then expression in *M. orbicularis* rapidly increased to *M. truncatula* levels or higher (**Figure 5**) and had not plateaued at the last measurement. Small oil bodies are usually associated with a low ratio of oil to oleosin (Shimada and Hara-Nishimura, 2010).

Starch containing amyloplasts were observed in both species but more commonly in *M. truncatula*, reflecting starch levels (**Figure 3B**).

### Cell Wall and Gross Seed Components Including Mucilage

The data obtained for the common storage products did not provide an explanation for the differences in the storage components of the two different Medicagos. Cell wall material is also a potential storage component (Daveby and Aman, 1993; Stombaugh et al., 2000, 2004) and the *M. orbicularis* cotyledon storage cells had a notably thicker cell wall (**Figures 4A–D**). The cell wall content of whole seeds (cell wall dry weight/seed dry weight) was 45.4% in *M. truncatula* and 50.0% in *M. orbicularis* while the cell wall content of the cotyledons alone was 41.7% in *M. truncatula* and 44.9% in *M. orbicularis*. In both cases, the cell wall content in *M. truncatula* was significantly lower compared to *M. orbicularis* (*p* < 0.05). This prompted an examination of the gross seed structure.

**FIGURE 6:**
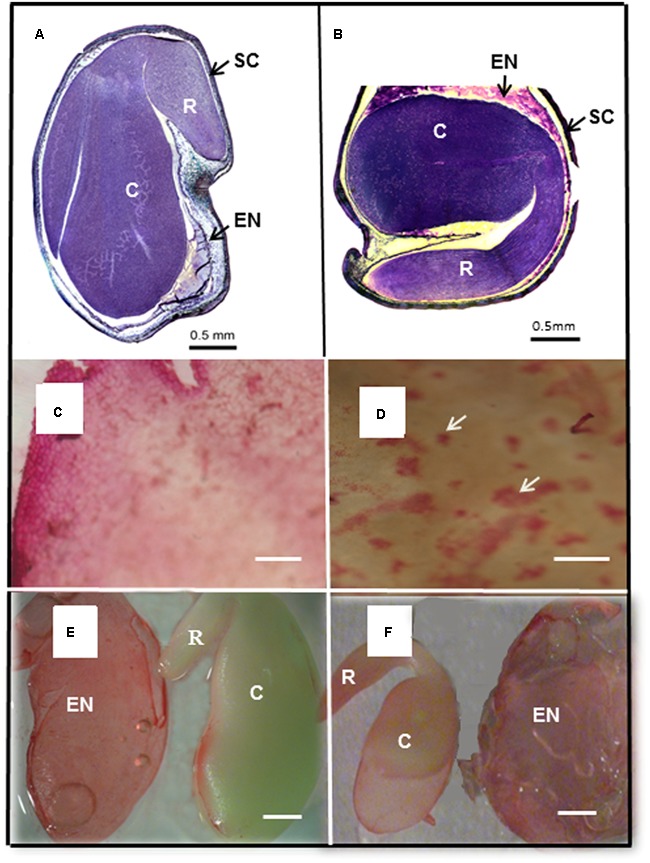
Seed morphology of *M. truncatula* and *M. orbicularis* and extruded endosperm mucilage. **(A)** Section (1 μm) through *M. truncatula* and **(B)** *M. orbicularis* seed stained with Toluidine Blue and Azur II. Seed coat (SC), endosperm (EN), embryonic cotyledons (C) and radicle (R). **(C)** Extruded endosperm mucilage with fine fibrous material visible and **(D)** mucilage (arrows) adhering to the inner side of the seed coat, stained with Ruthenium Red. **(E)** Isolated endosperm (EN) and embryo with cotyledons (C) and radicle (R) of *M. truncatula* and **(F)** *M. orbicularis* stained with Ruthenium Red. Bars **(A,B,E,F)** 0.5 mm, **(C,D)** 20 μm.

In legumes, the mature seed consists of an embryo, a reduced endosperm and a seed coat (**Figures 6A,B**). The seed coat, with several layers of cells is developed from the integuments of the ovule (Beeckman et al., 2000; Wang et al., 2012). In order to separate the seed coat from the cotyledons the seed was soaked in water. What we were surprised to observe was the extrusion of a gelatinous mass of mucilaginous material between the seed coat and the cotyledons, essentially enveloping the embryo. The mucilage (**Figures 6C,D**) characteristically stained with Ruthenium Red (Western et al., 2000; Verdier et al., 2013). The mucilage could be isolated as a gel-like sheet in which fibrillar material could be visualized (**Figure 6C**). After removal of most of the mucilage some traces of it still adhered to the inner seed coat in both species (**Figure 6D**). This differs from Arabidopsis with mucilage coming from the epidermal cell walls (Beeckman et al., 2000) which when hydrated forms a sphere around the seed. In Medicago the remnant endosperm forms a layer of cells surrounding the embryo (Wang and Grusak, 2005). The cell walls of these cells contain galactomannans (Reid, 1985; Edwards et al., 1999; Naoumkina et al., 2008), that releases as the mucilage gel forms between the embryo and the seed coat on hydration. This remnant endosperm layer can be isolated as a discrete envelope from around the embryo, staining with Ruthenium Red (**Figures 6E,F**).

**FIGURE 7:**
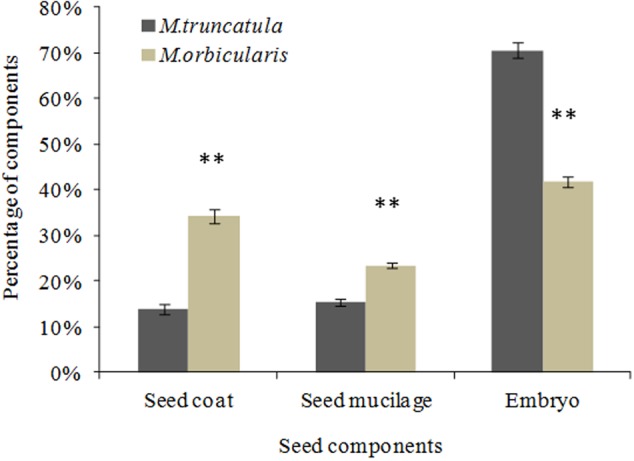
Proportion of seed dry weight of seed coat, extruded endosperm mucilage and embryo for *M. truncatula* and *M. orbicularis*. SE indicated and ^∗∗^*p* < 0.01 (*n* = 3).

After soaking the seed in water the seed was separated into the seed coat, extruded mucilage and cotyledons and the dry weights determined. *M. truncatula* seed had 14.0% seed coat, 15.3% extruded seed mucilage and 70.7% embryo while *M. orbicularis* had 34.4% seed coat, 23.6% extruded seed mucilage and 42.0% embryo (**Figure 7**). *M. orbicularis* seed had a higher content of seed coat and mucilage but lower embryo dry weight compared to *M. truncatula*. Measurement of mucilage by chemical analysis in *M. orbicularis*, in an older study, reported 18.6% seed mucilage rich in galactomannans (Tookey et al., 1962). The endosperm of *M. orbicularis* also stains more pink with Toluidine Blue and AzurII (**Figures 6A,B**). A pink colouration is consistent with increased pectinaceous material, such as mucilage (Verdier et al., 2013). Given the thicker cotyledon storage cell walls and extruded mucilage derived from the endosperm cell walls of *M. orbicularis* (**Figures 4A–D, 6, 7**); we investigated whether the increased mucilage and cell wall material could be related to changes in the expression of genes potentially involved in the regulation of mucilage biosynthesis and cell wall pectin biosynthesis. Cell wall polysaccharides can have an important storage role in addition to their function in the primary cell wall (Buckeridge et al., 2000).

### Expression of GLABRA2-Like and GALACTURONOSYLTRANSFERASE Genes

There is an inverse relationship between mucilage synthesis and oil production when *M. orbicularis* and *M. truncatula* are compared. Similarly, Arabidopsis mutants for the GL2 transcription factor (Shi et al., 2012) have reduced mucilage and increased oil production. Could the expression of a *GL2* transcription factor be a contributor to the differences in oil production between the two *Medicago* species?

**FIGURE 8:**
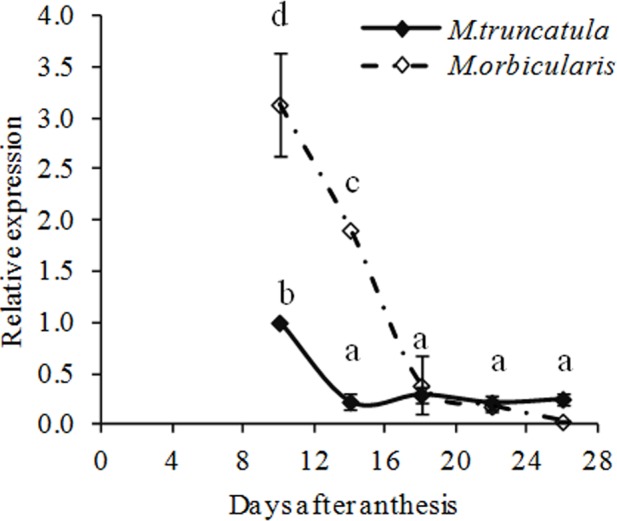
Relative expression (relative to 10 DAA) of *MtGLABRA2- like* (Medtr2g101720) during *M. truncatula* and *M. orbicularis* seed development. SE indicated and treatments with different letters are significantly different at the 0.05 probability level (*n* = 3).

We identified a *GL2* homolog in *M. truncatula* (Supplementary Figures S1a,b and Table S1) and gene expression of *MtGL2-like* was compared between *M. truncatula* and *M. orbicularis* (**Figure 8**). *Mt-GL2-like* expression decreased from 10 DAA onward in both species. From 10 to 18 DAA, expression of *MtGL2-like* in *M. truncatula* was markedly lower compared to *M. orbicularis* while from 18 DAA onward, the expression was at a low level in both species. This suggests that the MtGL2-like transcription factor is potentially a negative regulator of oil production, as in Arabidopsis.

**FIGURE 9:**
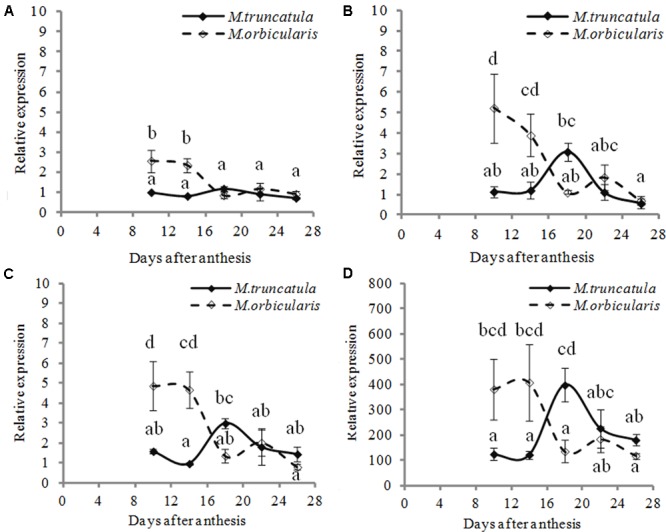
Relative expression (relative to 10 DAA) of four different *GAUT* genes during *M. truncatula* and *M. orbicularis* seed development. **(A)** *Mt-GAUT1* (Medtr7g075840). **(B)** *Mt-GAUT3* (Medtr3g107930). **(C)** *Mt-GAUT4* (Medtr 2g027740) **(D)** *Mt-GAUT7* (Medtr7g055600). SE indicated and treatments with different letters are significantly different at the 0.05 probability level (*n* = 3).

Given the increased cell wall material and the thicker cell walls in the *M. orbicularis* cotyledons, we also investigated the expression of *GALACTURONOSYLTRANSFERASES* (*GAUTs*) involved in the biosynthesis of the homogalacturonan pectin polysaccharide (Doong and Mohnen, 1998; Atmodjo et al., 2011), potentially a cell wall storage polysaccharide. We located seven *GAUT* genes (Supplementary Table S1) and studied their expression in the two Medicagos. Four of these genes showed enhanced expression in *M. orbicularis* (**Figure 9**) while the other three showed low expression with no difference between the two *Medicagos*. These data suggest cell wall pectins also contribute to the drain on carbon available for oil biosynthesis in *M. orbicularis*. In the case of *M. orbicularis* expression of both *GL2* and *GAUTs* is high early in seed development, just prior to the laying down of oil bodies (Wang et al., 2012).

### Gene Expression of Transcriptional Regulators of Seed Oil Storage

**FIGURE 10:**
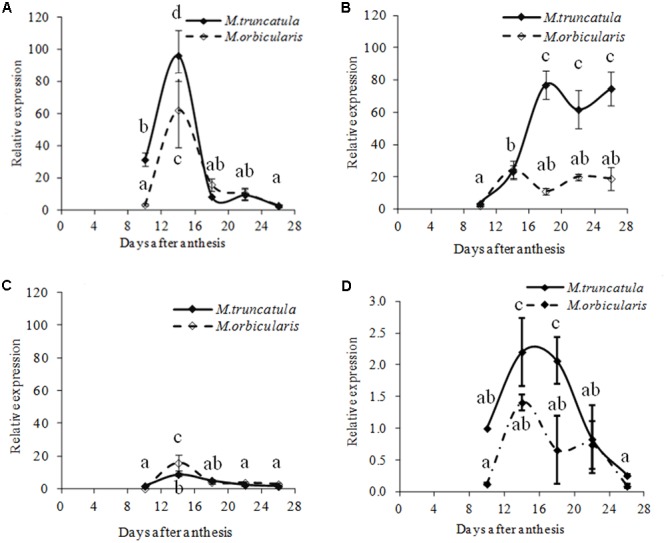
Relative expression (relative to 10 DAA) of four different transcription factors, linked to regulation of oil biosynthesis, during *M. truncatula* and *M. orbicularis* seed development. **(A)** *MtLEC1-LIKE* (Medtr4g133952), **(B)** *MtABI3* (Medtr7g059330), **(C)** *MtFUS3* (Medtr7g083700) and **(D)** *MtWRINKLE*-*like* (Medtr8g044070). SE indicated and treatments with different letters are significantly different at the 0.05 probability level (*n* = 3).

If in *M. orbicularis* there was more carbon being directed into mucilage and pectin polysaccharides, and less into oil, then it would be expected that transcriptional regulators of oil biosynthesis and storage would subsequently be down-regulated. Expression of homologs of the master regulators *MtLEC1-LIKE, MtFUS3* and *MtABI3* in Medicago was therefore carried out. *MtFUS3*, was expressed at a lower level in both species (**Figure 10C**). The expression patterns of *MtL1L* and *Mt*-*ABI3* are shown in **Figures 10A,B**. *MtL1L* expression from 10 to 18 DAA in *M. orbicularis* was lower than in *M. truncatula*, but similar after 18 DAA. *Mt-ABI3* expression increased from 10 DAA and reached a plateau at 18 DAA in *M. truncatula* and at 14 DAA in *M. orbicularis*. *ABI3* expression in *M. orbicularis* from 14 DAA onward was much lower than that in *M. truncatula*. The comparisons indicated that lower expression of *MtL1L* and *MtABI3* was associated with the lower seed protein and oil in *M. orbicularis*, with lower *MtL1L* expression in the early to middle stage and lower *ABI3* expression in the middle and late stage of seed development. The expression of the transcription factor *MtWRI-like*, which regulates fatty acid biosynthesis in Arabidopsis seed (To et al., 2012) was also reduced in *M. orbicularis* (**Figure 10D**).

## Discussion

The distinctive disparity of storage protein and oil content between *M. truncatula* and *M. orbicularis*, with their similar genetic background, provides a natural system for comparative studies of the regulation of seed storage accumulation (Tesfaye et al., 2006). The significant difference in oil between *M. truncatula* and *M. orbicularis*, shown in this study, is consistent with the report by Tonnet and Snudden (1974). This served as the basis of this investigation, following our earlier studies with *M. truncatula* on cellular and gene expression aspects of embryogenesis and storage filling (Wang et al., 2012; Kurdyukov et al., 2014).

The composition of major fatty acids was similar between *M. truncatula* and *M. orbicularis* (mainly palmitic, linolenic, α-linolenic, and oleic acids) but the quantity of individual fatty acids varied. Similar fatty acids were found by Djemel et al. (2005) in *M. truncatula* Jemalong 5. *Lotus japonicus* has higher stearate and linoleate levels than Medicago (Dam et al., 2009). Soybean seed oil is also primarily composed of five fatty acids (Kinney and Clemente, 2005) but with different levels, i.e., palmitic acid (∼13%), stearic acid (∼4%), oleic acid (∼18%), linoleic acid (∼55%), and linolenic acid (∼10%).

*Medicago orbicularis* has a much lower protein content compared to *M. truncatula*, but with a similar spectrum of storage proteins (also including lipoxygenases), though the contribution of the different genes differs (**Table 1** and Supplementary Table S3). The starch content is minor in both *M. truncatula* and *M. orbicularis*, but is higher in *M. truncatula*. Why are all the common storage components – oil, protein and starch, lower in *M. orbicularis*?

*Medicago orbicularis* has a higher seed dry weight compared to *M. truncatula.* Nevertheless, *M. truncatula* has three times the protein and oil relative to *M. orbicularis.* Further investigation indicated that *M. orbicularis* has a much higher seed coat dry weight than *M. truncatula* (**Figure 7**). The seed coat does not store significant storage protein and oil which is characteristically stored in the cotyledons as shown in the ultrastructure images (**Figure 4**). If the seed coat is deducted, *M. orbicularis* has a lower embryo dry weight compared to *M. truncatula* (**Figure 8**). This contributes to the protein and oil differences between the embryos of both species. However, taking this into account, *M. truncatula* embryos still have approximately 80% more protein and oil relative to *M. orbicularis*. So what is happening in the *M. orbicularis* embryo to reduce seed oil?

What is of special interest is the mucilage which on hydration forms a layer between the cotyledons and seed coat. Commonly when a seed has substantive mucilage it forms a gel-like capsule surrounding the seed (Western, 2012) where it could mediate seed dispersal through adhesion or possibly facilitate seed hydration (Deng et al., 2012; Western, 2012). There is little recent information on legume mucilage in the literature (Moise et al., 2005; Western, 2012), including *M. truncatula* (Wang and Grusak, 2005; Verdier et al., 2013). However, a much older publication identified mucilage in *M. orbicularis* at a similar level to the accession used here (18.6% on a protein free basis, Tookey et al., 1962). Subsequent investigation by Tookey and Jones (1965) of 300 legume species found a negative correlation between oil plus protein and mucilage. Mucilage is carbon rich [a galactomannan in legumes (Tookey et al., 1962; Edwards et al., 1999; Naoumkina et al., 2008)] and the increased mucilage in *M. orbicularis* correlates with the decreased oil production relative to *M. truncatula*. Recently there has been more attention given to mucilage biochemistry and formation in Arabidopsis (e.g., Saez-Aguayo et al., 2013; Voiniciuc et al., 2013). Of particular interest is the demonstration by Shi et al. (2012) showing that the *glabra2* mutant deficient in mucilage produced more oil. Consistent with this, *M. truncatula* with low expression of a *GL2* homolog, has more oil and less mucilage than *M. orbicularis.* In *M. orbicularis* there is a surge of *GL2* expression just prior to oil accumulation starting (**Figures 5, 8**). This is consistent with reducing the carbon flow into oil in *M. orbicularis*. It has to be noted that in Arabidopsis mucilage is rich in rhamnogalacturonan I (Macquet et al., 2007) while it is rich in galactomannans in legumes (Tookey et al., 1962; Edwards et al., 1999; Naoumkina et al., 2008). However, it is feasible that glycotransferases have similar regulation strategies in cell wall – linked polysaccharide assembly. It also seems likely that the increased biosynthesis of cell wall pectins (homogalacturonans) contributes to lower oil and protein in *M. orbicularis* cotyledons, based on the expression of *GAUT* genes. This is also consistent with total cell wall measurements, electron micrographs of cotyledon cells (**Figure 4**) and the storage role of cell wall polysaccharides (Buckeridge et al., 2000; Buckeridge, 2010).

We have focused on the embryo and the relationship to the extruded endosperm mucilage and cotyledon cell walls, but carbon also flows to the seed coat. In *M. orbicularis* the seed coat represents a greater proportion of the seed and as discussed above there are smaller embryos in *M. orbicularis* (**Figure 7**). There is also another aspect of the seed coat which differs between the two Medicagos. *M. orbicularis* has a very hard seed coat which is reddish brown, whereas *M. truncatula* has a light brown seed coat which is not as hard. This may reflect differences in phenylpropanoids which are negatively correlated with omega-3 fatty acids in flax (Ramsay et al., 2014). In Arabidopsis there is a negative correlation between proanthocyanidins and the amount of fatty acids (Wang et al., 2014). Further comparative metabolomic investigations between the Medicago seed coats is warranted, in conjunction with further studies on the cotyledons, as a precursor to further gene regulation studies.

The final storage outcome depends on the efficiency of the biosynthetic pathways once carbon is uploaded into the embryo. The master transcription factors AtLEC1/L1L, AtLEC2, AtFUS3, and AtABI3 have been shown to regulate seed protein and oil accumulation in Arabidopsis and homologs have been identified in *M. truncatula* (Verdier et al., 2008; Thompson et al., 2009; Tang et al., 2014). *In situ* hybridisation of *MtABI3* in Kurdyukov et al. (2014) showed expression throughout the whole *M. truncatula* cotyledon, after the torpedo stage, supporting the role of the *MtABI3* gene in *M. truncatula*. In *M. orbicularis, MtFUS3* expression was relatively low and similar to *M. truncatula*. *MtL1L* and *MtABI3* have reduced expression in *M. orbicularis*, consistent with lower accumulation of seed protein and oil in *M. orbicularis* (Kagaya et al., 2005; Tan et al., 2011). These data also support the role of these two transcription factors in seed storage in *M. truncatula* (Verdier et al., 2013). The expression of the *MtWRI-like* transcription factor, regulating fatty acid biosynthesis (To et al., 2012), also supports lower oil production in *M. orbicularis.*

The differences between *M. truncatula* and *M. orbicularis* reflects differences in ecology with *M. orbicularis* having a very thick seed coat (Patanè and Gresta, 2006), thick walled cells and increased mucilage which would facilitate hydration (Deng et al., 2012). These characteristics would be useful in more arid conditions.

The differences highlighted between *M. orbicularis* and *M. truncatula* provide additional approaches into carbon partitioning and the modification of oil content in legumes (Song et al., 2017). The direct testing of the transcription factor GL2 and the GAUT biosynthesis genes can be carried out in *M. truncatula*, using overexpression or gene knockout using CRISPR/Cas9 (Luo et al., 2016), where there are robust transformation protocols (Song et al., 2013).

## Author Contributions

All authors contributed to the drafting/writing and interpretation of the work, and approve and are accountable for the final version of the manuscript. In addition, YS and RR designed experimentation. LH did the statistics. YS did the majority of experiments. X-DW did the cytology. YS, NS, and SW did the proteomics and MG supervised and interpreted the fatty acid analysis.

## Conflict of Interest Statement

The authors declare that the research was conducted in the absence of any commercial or financial relationships that could be construed as a potential conflict of interest.

## Footnotes

1 http://www.agrf.org.au/

2 https://phytozome.jgi.doe.gov/biomart/martview/

3 www.ncbi.nlm.nih.gov

4 www.phytozome.net
