# Supplementary material for: Regulation of Carbon Partitioning in the Seed of the Model Legume Medicago truncatula and Medicago orbicularis: A Comparative Approach
Source: Front Plant Sci. 2017 Dec 12;8:2070. doi: 10.3389/fpls.2017.02070 (PMC5733034; doi:10.3389/fpls.2017.02070)
Supplement: Supplementary file 1 [file Table_1.PDF]

Supplementary Table S1. Genes used in gene expression analyses

Gene Name	Gene Locus Mt4.0	NCBI Reference amino acid sequence	Gene Locus closest Arabidopsis homologue and % identity	Gene Locus closest Legume homologue and % identity
ABI3	Medtr7g059330	XP_013448806.1	At3g24650 (46%)	Chickpea LOC101503180 (74%)
FUSCA3	Medtr7g083700	XP_003624470.1	At3g26790 (47%)	Chickpea LOC101500621 (79%)
LEC1-LIKE	Medtr4g133952	XP_013458658.1	At5g47670 (60%)	Chickpea LOC101496026 (81%)
WRINKLED	Medtr8g044070	XP_003628132.2	At1g79700 (54%)	Chickpea LOC101488375 (73%)
GAPDH	Medtr4g103920	XP_003608875.1	At3g04120 (97%)	Chickpea LOC101491441 (88%)
GAUT1	Medtr7g075840	XP_003623804.2	At3g61130 (74%)	Chickpea LOC101506824 (93%)
GAUT3	Medtr3g107930	XP_O13461906.1	At4g38270 (68%)	Chickpea LOC101510161 (87%)
GAUT4	Medtr2g027740	XP_003594361.1	At2g46480 (45%)	Chickpea LOC101502481 (86%)
GAUT7	Medtr7g074680	XP_003623702.1	At2g38650 (55%)	Chickpea LOC101497487 (85%)
GAUT8	Medtr7g055600	Xp_003622860.1	At3g25140 (89%)	Chickpea LOC101507690 (97%)
GAUT12	Medtr2g082650	XP_003596616.1	At5g54690 (82%)	Chickpea LOC101514507 (94%)
GAUT14	Medtr7g012370	XP_013447558.1	At5g15470 (84%)	Chickpea LOC101505839 (96%)
*GLABRA*2	Medtr2g101720	XP_003597736.1	At1g79840 (61%)	Chickpea LOC101512878 (86%)
OLEOSIN	Medtr3g109190	XP_003603568.1	At2g25890 (57%)	Chickpea LOC101488878 (83%)
