# Supplementary material for: Regulation of Carbon Partitioning in the Seed of the Model Legume Medicago truncatula and Medicago orbicularis: A Comparative Approach
Source: Front Plant Sci. 2017 Dec 12;8:2070. doi: 10.3389/fpls.2017.02070 (PMC5733034; doi:10.3389/fpls.2017.02070)
Supplement: Supplementary file 2 [file Table_2.PDF]

Supplementary Table S2. Primer sequences used for qPCR in *M. truncatula* and *M. orbicularis* and their locus in M. truncatula.

**Homologous gene name**	**Locus in *M. truncatula***	**Primer sequences (5' to 3')**
WRINKLED	Medtr8g044070	TAGATGGACCGGAAGGTTTG
		CGTCACTGCATTGATTCCTC
OLEOSIN	Medtr3g109190	TATCACTCATTGCTGGTGGCTTT
		GGGCGGTTACCAGAGACATAGTT
GAPDH	Medtr4g103920	GACTTTATTGGTGATACCAGGTCG
		GGTCAACCACACGGGTACTGTAA
L1L	Medtr4g133592	TGAGCTTGAAGGTGATCGTACCTC
		TGGTGCAGCATTACCATAATACCC
FUS3	Medtr7g083700	GCACGTGAAATCGATCATAGGAG
		TCTGCTGCTTTCTTAGGCAACAC
ABI3	Medtr7g059330	TGCAGACACAGAGTTCTCACCAG
		GACTTCCGACATCACTTTGCTTC
GL2	Medtr2g101720	AGGGGAGGAAGGGAGTACAA
		AGCGCTTCCATGACTCTGAT
GAUT1	Medtr7g075840	GCATATATCACAGGTGGCAGAA
		GTCCAGTAAGGGCGATACTTTG
GAUT3	Medtr3g107930	CTTTCTATGGCAAAGGACCAAC
		CTCTTTGCCAACATTTCCTTTC
GAUT4	Medtr2g027740	ATGAAGGCAATGGAGCAAAC
		TGATTTGGAAACTGCTGCTG
GAUT7	Medtr7g074680	GCTTGCAGCATCAGTGGTTA
		TGCGAAATGAAACACGAAAC
GAUT8	Medtr7g055600	GTCAATTCCGAAGAGCTTGC
		TTGCGTTCTTTGTTGCTGAG
GAUT12	Medtr2g082650	GAGGTTCATCGGCTATAGTTGC
		ACTGCTCCATTCACTTTTCCAT
GAUT14	Medtr7g012370	TCAAAATGGGATCAGGGATTAC
		CTTCAAGGTCAATTTCCCAAAG
