# Supplementary material for: Regulation of Carbon Partitioning in the Seed of the Model Legume Medicago truncatula and Medicago orbicularis: A Comparative Approach
Source: Front Plant Sci. 2017 Dec 12;8:2070. doi: 10.3389/fpls.2017.02070 (PMC5733034; doi:10.3389/fpls.2017.02070)
Supplement: Supplementary file 3 [file Table_3.PDF]

## Supplementary Table S3

### Sequence of tabulation – days after anthesis

*M. truncatula* 22 d

*M. orbicularis* 22 d

*M. truncatula* 18 d

*M. orbicularis* 18 d

*M. truncatula* 14 d

*M. orbicularis* 14 d

*M. truncatula* 12 d

*M. orbicularis* 12 d

**M.truncatula 22 d**

| Accession | Protein | Scores | S.Coverage | MW [kDa] | pI | Peptides | Rank |
|---|---|---|---|---|---|---|---|
| IMGA\|Medtr1g072600.1 | Legumin B (Fragment) | 3196.2 (M:3196. | 42.7 | 67.2 | 6.5 | 42 | 1 |
| IMGA\|Medtr1g072630.1 | Legumin B | 2402.4 (M:2402. | 47.1 | 65.3 | 6.1 | 10 | 2 |
| IMGA\|Medtr1g072630.2 | Legumin B (Fragment) | 2131.7 (M:2131. | 41.0 | 63.5 | 6.4 | 1 | 3 |
| IMGA\|Medtr1g072610.2 | Legumin B (Fragment) | 2005.7 (M:2005. | 39.7 | 63.4 | 6.4 | 1 | 4 |
| IMGA\|Medtr7g079820.1 | Convicilin (Fragment) | 1797.9 (M:1797. | 52.7 | 53.7 | 5.6 | 30 | 5 |
| IMGA\|Medtr7g079740.1 | Convicilin (Fragment) | 1776.6 (M:1776. | 53.3 | 54.2 | 5.6 | 13 | 6 |
| IMGA\|Medtr7g079780.1 | Convicilin (Fragment) | 1755.4 (M:1755. | 47.8 | 53.2 | 5.5 | 2 | 7 |
| IMGA\|Medtr7g079730.1 | Convicilin (Fragment) | 1631.6 (M:1631. | 42.5 | 53.1 | 5.3 | 3 | 8 |
| IMGA\|Medtr7g079770.1 | Provicilin (Fragment) | 1379.3 (M:1379. | 49.5 | 53.1 | 5.3 | 4 | 9 |
| IMGA\|Medtr2g099560.1 | Lipoxygenase | 735.7 (M:735.7) | 17.0 | 96.9 | 5.8 | 11 | 10 |
| IMGA\|Medtr2g083160.1 | Conglutin | 592.0 (M:592.0) | 25.0 | 16.8 | 6.3 | 9 | 11 |
| IMGA\|Medtr7g113790.1 | Basic 7S globulin 2 | 553.6 (M:553.6) | 22.0 | 48.1 | 10.0 | 10 | 12 |
| IMGA\|Medtr5g019780.1 | Vicilin-like antimicrobial peptides 2-3 (Fragment) | 543.9 (M:543.9) | 15.4 | 86.4 | 5.2 | 9 | 13 |
| IMGA\|Medtr2g099570.1 | Seed lipoxygenase-3 | 518.9 (M:518.9) | 12.5 | 97.3 | 6.1 | 6 | 14 |
| VCLC_PEA | Vicilin precursor | 369.2 (M:369.2) | 11.1 | 52.2 | 5.3 | 2 | 15 |
| IMGA\|Medtr4g060780.1 | Allergen Gly m Bd (Fragment) | 349.4 (M:349.4) | 16.3 | 52.8 | 5.7 | 5 | 16 |
| IMGA\|Medtr4g051270.1 | Ribulose bisphosphate carboxylase large chain | 347.1 (M:347.1) | 14.7 | 50.6 | 6.2 | 6 | 17 |
| IMGA\|Medtr4g103920.1 | Glyceraldehyde-3-phosphate dehydrogenase | 342.0 (M:342.0) | 31.6 | 36.6 | 6.7 | 6 | 18 |
| IMGA\|Medtr1g071710.1 | Vicilin-like antimicrobial peptides 2-2 | 243.8 (M:243.8) | 14.8 | 56.0 | 6.0 | 5 | 19 |
| BIP_SPIOL | Luminal-binding protein precursor (BiP) | 217.8 (M:217.8) | 7.2 | 73.5 | 4.9 | 3 | 20 |
| IMGA\|Medtr3g085850.1 | Glyceraldehyde 3-phosphate dehydrogenase | 205.0 (M:205.0) | 17.6 | 37.0 | 7.7 | 1 | 21 |
| IMGA\|Medtr1g108770.1 | ATP synthase subunit beta | 185.6 (M:185.6) | 3.5 | 120.9 | 5.8 | 3 | 22 |
| LEGA_PEA | Legumin A | 179.7 (M:179.7) | 3.9 | 58.8 | 6.2 | 2 | 23 |
| IMGA\|Medtr4g051370.1 | Photosystem II CP47 chlorophyll apoprotein | 164.2 (M:164.2) | 7.7 | 107.3 | 7.3 | 4 | 24 |
| IMGA\|Medtr3g114420.1 | Subtilisin-type protease | 156.6 (M:156.6) | 2.2 | 82.4 | 8.4 | 2 | 25 |
| ATPAM_PEA | ATP synthase subunit alpha | 152.7 (M:152.7) | 6.1 | 55.0 | 6.0 | 2 | 26 |
| ENO_RICCO | Enolase (EC 4.2.1.11) | 145.6 (M:145.6) | 7.4 | 47.9 | 5.5 | 2 | 27 |
| IMGA\|Medtr4g132270.2 | Lactoylglutathione lyase | 140.4 (M:140.4) | 10.7 | 27.0 | 4.8 | 2 | 28 |
| GLYG1_SOYBN | Glycinin G1 | 136.3 (M:136.3) | 4.2 | 55.7 | 5.8 | 3 | 29 |
| IMGA\|Medtr7g113650.1 | Glucose and ribitol dehydrogenase homolog 1 | 135.0 (M:135.0) | 19.5 | 31.8 | 7.7 | 3 | 30 |
| LEG_CICAR | Legumin | 133.3 (M:133.3) | 2.4 | 56.2 | 6.2 | 2 | 31 |
| ENO_SOLLC | Enolase | 132.8 (M:132.8) | 9.0 | 47.8 | 5.6 | 1 | 32 |
| IMGA\|Medtr5g025120.1 | 60S ribosomal protein | 109.7 (M:109.7) | 7.8 | 44.9 | 10.9 | 2 | 33 |
| ENO_ORYSJ | Enolase | 106.0 (M:106.0) | 5.6 | 47.9 | 5.3 | 2 | 34 |
| IMGA\|Medtr3g118030.1 | 60S ribosomal protein L5 | 105.3 (M:105.3) | 6.3 | 37.5 | 9.6 | 2 | 35 |
| RUBB_SECCE | RuBisCO large subunit-binding protein subunit beta | 100.3 (M:100.3) | 5.0 | 53.4 | 4.7 | 1 | 36 |
| ENO2_HEVBR | Enolase 2 (EC 4.2.1.11) | 99.3 (M:99.3) | 3.8 | 47.9 | 5.9 | 1 | 37 |
| IMGA\|Medtr3g109190.1 | Oleosin | 97.7 (M:97.7) | 17.4 | 16.8 | 9.3 | 1 | 38 |
| IMGA\|Medtr2g014030.1 | 40S ribosomal protein S6 | 90.6 (M:90.6) | 6.0 | 28.2 | 11.5 | 1 | 39 |
| ATPA_POPEU | ATP synthase subunit alpha | 88.6 (M:88.6) | 16.3 | 10.6 | 10.5 | 1 | 40 |
| IMGA\|Medtr8g102620.1 | Malic enzyme (Fragment) | 86.9 (M:86.9) | 2.5 | 65.3 | 6.0 | 1 | 41 |
| IMGA\|Medtr7g086300.2 | Methionine synthase | 86.9 (M:86.9) | 5.4 | 83.1 | 5.8 | 2 | 42 |
| PSBE_OENBE | Cytochrome b559 subunit alpha | 82.8 (M:82.8) | 14.5 | 9.3 | 4.9 | 1 | 43 |
| IMGA\|Medtr7g069980.1 | Ferritin-1, chloroplastic | 82.8 (M:82.8) | 5.9 | 28.8 | 6.0 | 2 | 44 |
| IMGA\|Medtr4g035360.1 | GDSL esterase/lipase | 79.9 (M:79.9) | 2.0 | 112.9 | 9.4 | 1 | 45 |
| IMGA\|Medtr5g074860.2 | Peroxidase | 79.6 (M:79.6) | 5.2 | 31.4 | 10.4 | 1 | 46 |
| IMGA\|Medtr7g088680.1 | Nascent polypeptide-associated complex subunit alpha-like protein | 77.7 (M:77.7) | 8.4 | 22.1 | 4.2 | 1 | 47 |
| H2B2_SOLLC | Histone H2B.2 | 73.4 (M:73.4) | 10.7 | 15.4 | 10.6 | 1 | 48 |
| IMGA\|Medtr8g072010.1 | Carboxylic ester hydrolase | 73.2 (M:73.2) | 4.7 | 45.6 | 4.9 | 1 | 49 |
| RNH2A_ARATH | Ribonuclease H2 subunit A | 73.1 (M:73.1) | 5.4 | 33.1 | 6.3 | 2 | 50 |
| IMGA\|Medtr7g052690.1 | Early tobacco anther 1 | 71.8 (M:71.8) | 13.9 | 16.9 | 4.5 | 1 | 51 |
| CHLB_CHAGL | Light-independent protochlorophyllide reductase subunit B | 68.2 (M:68.2) | 3.7 | 58.3 | 5.9 | 1 | 52 |
| RS5_NICPL | 40S ribosomal protein S5 (Fragment) | 67.9 (M:67.9) | 9.7 | 17.1 | 11.1 | 1 | 53 |
| IMGA\|Medtr6g018300.1 | Ribulose bisphosphate carboxylase small chain (Fragment) | 67.7 (M:67.7) | 7.9 | 19.8 | 9.4 | 1 | 54 |
| PGKY_WHEAT | Phosphoglycerate kinase, cytosolic (EC 2.7.2.3) | 66.8 (M:66.8) | 4.2 | 42.1 | 5.5 | 1 | 55 |
| RUB1_BRANA | RuBisCO large subunit-binding protein subunit alpha | 62.4 (M:62.4) | 3.1 | 57.7 | 4.7 | 1 | 56 |
| IMGA\|Medtr6g005040.1 | Oleosin 1 | 61.3 (M:61.3) | 7.3 | 20.7 | 9.3 | 1 | 57 |
| IMGA\|Medtr6g021800.1 | Elongation factor 1-alpha | 60.5 (M:60.5) | 2.4 | 109.7 | 9.8 | 1 | 58 |
| IMGA\|Medtr1g088450.1 | 60S ribosomal protein L22-like | 60.4 (M:60.4) | 10.9 | 13.6 | 10.0 | 1 | 59 |
| IMGA\|Medtr3g064480.1 | UTP--glucose-1-phosphate uridylyltransferase | 59.9 (M:59.9) | 6.4 | 26.1 | -1.6 | 1 | 60 |
| AL7A1_PEA | Aldehyde dehydrogenase family 7 member A1 | 59.6 (M:59.6) | 3.9 | 53.8 | 5.5 | 1 | 61 |
| IMGA\|Medtr4g115970.2 | Vacuolar proton-inorganic pyrophosphatase | 58.7 (M:58.7) | 3.1 | 75.6 | 4.9 | 1 | 62 |
| AGAL_CAPAA | Alpha-galactosidase (Fragment) | 57.4 (M:57.4) | 100.0 | 1.4 | 6.7 | 1 | 63 |
| IMGA\|Medtr4g131740.1 | Glucose and ribitol dehydrogenase homolog 1 | 56.4 (M:56.4) | 4.4 | 32.0 | 6.0 | 1 | 64 |
| IMGA\|Medtr8g079230.1 | Ubiquitin | 56.0 (M:56.0) | 30.8 | 5.8 | 4.9 | 1 | 65 |
| IMGA\|AC146630_2.1 | 2-cys peroxiredoxin | 55.7 (M:55.7) | 9.4 | 29.0 | 6.1 | 1 | 66 |
| CB13_SOLLC | Chlorophyll a-b binding protein 8 | 55.0 (M:55.0) | 6.2 | 29.3 | 9.4 | 1 | 67 |
| IMGA\|Medtr6g014480.2 | Phosphorylase | 54.9 (M:54.9) | 2.3 | 83.9 | 5.2 | 1 | 68 |
| IMGA\|Medtr3g087590.2 | L-myo inositol-1 phosphate synthase 1 | 54.9 (M:54.9) | 4.7 | 51.9 | 5.2 | 1 | 69 |
| TBB3_SOYBN | Tubulin beta chain (Beta tubulin) (Fragment) | 54.8 (M:54.8) | 6.4 | 45.7 | 5.6 | 1 | 70 |
| ALF_CICAR | Fructose-bisphosphate aldolase, cytoplasmic isozyme (EC 4.1.2.13) | 54.4 (M:54.4) | 6.4 | 38.4 | 6.2 | 1 | 71 |
| IMGA\|Medtr4g124660.4 | Sucrose synthase | 53.8 (M:53.8) | 4.4 | 54.2 | 6.0 | 1 | 72 |
| RS281_ARATH | 40S ribosomal protein S28-1 | 53.5 (M:53.5) | 18.8 | 7.4 | 11.3 | 1 | 73 |
| GBLPB_ORYSJ | Guanine nucleotide-binding protein subunit beta-like protein B | 52.9 (M:52.9) | 7.4 | 36.4 | 6.1 | 1 | 74 |
| IMGA\|Medtr2g005570.2 | Elongation factor 1-gamma | 52.0 (M:52.0) | 4.5 | 47.7 | 7.7 | 1 | 75 |
| IMGA\|Medtr1g099840.1 | Heat shock protein 90 (Fragment) | 51.9 (M:51.9) | 1.7 | 79.3 | 4.8 | 1 | 76 |
| CLPB2_ARATH | Putative chaperone protein ClpB2 | 51.3 (M:51.3) | 2.4 | 68.9 | 8.9 | 1 | 77 |
| IMGA\|Medtr6g052140.1 | 40S ribosomal protein S3 | 50.8 (M:50.8) | 6.0 | 26.2 | 10.4 | 1 | 78 |
| IMGA\|Medtr3g114850.1 | Plastocyanin | 50.8 (M:50.8) | 14.4 | 17.1 | 4.8 | 1 | 79 |
| MDHC_MEDSA | Malate dehydrogenase, cytoplasmic | 50.7 (M:50.7) | 6.0 | 35.5 | 6.4 | 1 | 80 |
| IMGA\|Medtr5g018940.1 | 40S ribosomal protein S4 | 49.4 (M:49.4) | 7.2 | 29.9 | 10.8 | 1 | 81 |
| IMGA\|Medtr6g021670.1 | 40S ribosomal protein S7-like protein | 49.1 (M:49.1) | 13.6 | 21.9 | 10.3 | 1 | 82 |
| IMGA\|Medtr7g072730.1 | Non-specific lipid-transfer protein | 48.5 (M:48.5) | 21.4 | 12.4 | 4.8 | 1 | 83 |
| ACT2_SOLTU | Actin-46 | 47.8 (M:47.8) | 4.8 | 37.1 | 5.2 | 1 | 84 |
| IMGA\|Medtr2g064660.2 | Photosystem II 10 kDa polypeptide | 47.6 (M:47.6) | 11.8 | 13.6 | 10.2 | 1 | 85 |
| IMGA\|Medtr2g008960.1 | Unknown Protein | 46.8 (M:46.8) | 4.5 | 42.4 | 9.1 | 1 | 86 |
| IMGA\|Medtr4g131390.1 | Outer membrane lipoprotein | 46.8 (M:46.8) | 8.7 | 21.4 | 6.1 | 1 | 87 |
| RS15_ORYSJ | 40S ribosomal protein S15 | 46.7 (M:46.7) | 11.0 | 17.4 | 10.6 | 1 | 88 |

| | | | | | | | |
|---|---|---|---|---|---|---|---|
| ACCC2_POPTR | Biotin carboxylase 2, chloroplastic | 46.4 (M:46.4) | 3.0 | 57.5 | 6.5 | 1 | 89 |
| ARFO_ARATH | Putative auxin response factor 15 | 45.6 (M:45.6) | 2.9 | 67.4 | 9.4 | 1 | 90 |
| IMGA\|Medtr3g088450.1 | Photosystem Q(B) protein | 45.2 (M:45.2) | 9.6 | 12.8 | 10.5 | 1 | 91 |
| IMGA\|AC225458_41.1 | Unknown Protein | 44.5 (M:44.5) | 17.1 | 20.2 | 4.8 | 1 | 92 |
| SODC5_MAIZE | Superoxide dismutase [Cu-Zn] | 44.4 (M:44.4) | 6.6 | 15.1 | 5.6 | 1 | 93 |
| P2C68_ORYSJ | Probable protein phosphatase 2C | 44.4 (M:44.4) | 5.0 | 37.7 | 6.2 | 1 | 94 |
| RH26_ARATH | DEAD-box ATP-dependent RNA helicase 26 | 43.4 (M:43.4) | 1.2 | 94.1 | 10.1 | 1 | 95 |
| CCA14_ORYSJ | Cyclin-A1-4 | 41.2 (M:41.2) | 5.1 | 40.6 | 5.3 | 1 | 96 |
| PARP1_ARATH | Poly [ADP-ribose] polymerase 1 | 40.8 (M:40.8) | 1.7 | 111.2 | 9.5 | 1 | 97 |
| IMGA\|Medtr5g090420.1 | WD repeat-containing protein 26 | 40.6 (M:40.6) | 2.1 | 63.6 | 6.3 | 1 | 98 |
| IMGA\|Medtr1g008240.1 | Cc-nbs resistance protein (Fragment) | 40.6 (M:40.6) | 2.2 | 143.6 | 6.2 | 1 | 99 |
| IMGA\|Medtr7g079500.1 | ALBINO3-like protein 2, chloroplastic | 40.4 (M:40.4) | 2.5 | 66.8 | 6.7 | 1 | 100 |
| HIBC4_ARATH | 3-hydroxyisobutyryl-CoA hydrolase-like protein 1 | 40.4 (M:40.4) | 4.7 | 44.7 | 6.4 | 1 | 101 |
| IMGA\|Medtr3g027280.1 | Vegetative lectin | 40.1 (M:40.1) | 5.0 | 30.2 | 7.7 | 1 | 102 |
| PDV2_ARATH | Plastid division protein PDV2 | 40.0 (M:40.0) | 9.1 | 33.6 | 5.1 | 1 | 103 |
| SYT5_ARATH | Synaptotagmin-5 | 39.9 (M:39.9) | 2.1 | 62.9 | 5.5 | 1 | 104 |
| SWT7A_ORYSJ | Bidirectional sugar transporter SWEET7a | 39.8 (M:39.8) | 11.2 | 28.7 | 10.2 | 1 | 105 |
| IMGA\|Medtr8g076910.1 | Tir-nbs-lrr resistance protein (Fragment) | 39.4 (M:39.4) | 1.1 | 126.6 | 6.2 | 1 | 106 |
| ADT1_ARATH | ADP,ATP carrier protein 1 | 39.3 (M:39.3) | 4.2 | 41.4 | 10.2 | 1 | 107 |
| IMGA\|Medtr2g013640.1 | Unknown Protein | 39.2 (M:39.2) | 1.0 | 106.3 | 9.8 | 1 | 108 |
| C81F1_ARATH | Cytochrome P450 | 39.2 (M:39.2) | 1.4 | 57.5 | 8.9 | 1 | 109 |
| IMGA\|Medtr2g064060.1 | Unknown Protein | 38.8 (M:38.8) | 27.9 | 8.0 | 10.5 | 1 | 110 |
| YCF2_PSINU | Protein ycf2 | 38.8 (M:38.8) | 1.0 | 273.4 | 9.6 | 1 | 111 |
| IMGA\|Medtr8g085550.1 | Unknown Protein | 38.7 (M:38.7) | 9.9 | 17.2 | 4.8 | 1 | 112 |
| CRR3_ARATH | Cysteine-rich repeat secretory protein 3 | 38.3 (M:38.3) | 8.8 | 32.7 | 9.4 | 1 | 113 |
| GLTB2_ARATH | Ferredoxin-dependent glutamate synthase 2 | 38.3 (M:38.3) | 0.7 | 177.6 | 6.6 | 1 | 114 |
| MAN2_SOLLC | Mannan endo-1,4-beta-mannosidase 2 | 38.3 (M:38.3) | 3.6 | 46.8 | 5.6 | 1 | 115 |
| IMGA\|Medtr5g078260.1 | ATP-dependent DNA helicase PIF1 | 38.2 (M:38.2) | 1.2 | 178.4 | 6.1 | 1 | 116 |
| CCSA_PINTH | Cytochrome c biogenesis protein | 38.2 (M:38.2) | 8.1 | 36.2 | 7.9 | 1 | 117 |
| IMGA\|Medtr2g086420.1 | BZIP transcription factor | 38.0 (M:38.0) | 2.6 | 55.4 | 6.4 | 1 | 118 |
| IMGA\|Medtr4g127700.1 | Unknown Protein | 37.8 (M:37.8) | 16.0 | 18.8 | 4.7 | 1 | 119 |
| BH130_ARATH | Transcription factor bHLH130 | 37.5 (M:37.5) | 3.6 | 39.9 | 8.8 | 1 | 120 |
| FH3_ARATH | Formin-like protein 3 | 37.5 (M:37.5) | 1.9 | 85.8 | 7.7 | 1 | 121 |
| IMGA\|Medtr3g099380.2 | 14-3-3-like protein | 37.5 (M:37.5) | 6.8 | 23.3 | 5.4 | 1 | 122 |
| 14331_ARATH | 14-3-3-like protein GF14 | 37.4 (M:37.4) | 6.4 | 29.9 | 4.5 | 1 | 123 |
| EF1D2_ARATH | Elongation factor 1-delta 2 | 37.4 (M:37.4) | 9.5 | 25.3 | 4.3 | 1 | 124 |
| TGT1_ARATH | Trihelix transcription factor GT-1 | 37.3 (M:37.3) | 2.5 | 46.6 | 6.4 | 1 | 125 |
| BH139_ARATH | Transcription factor bHLH139 | 37.2 (M:37.2) | 5.8 | 25.1 | 6.6 | 1 | 126 |
| URIC1_CANLI | Uricase-2 isozyme 1 (EC 1.7.3.3) | 37.0 (M:37.0) | 6.8 | 34.9 | 9.2 | 1 | 127 |
| ARRS_MAIZE | Anthocyanin regulatory R-S protein | 36.9 (M:36.9) | 3.4 | 66.6 | 4.9 | 1 | 128 |
| IMGA\|Medtr4g127690.1 | Unknown Protein | 36.9 (M:36.9) | 7.7 | 20.8 | 10.4 | 1 | 129 |
| IMGA\|Medtr7g116430.1 | Translational activator | 36.9 (M:36.9) | 0.9 | 300.6 | 6.5 | 1 | 130 |
| IMGA\|AC235667_9.1 | Embryonic abundant protein-like protein (Fragment) | 36.8 (M:36.8) | 3.7 | 37.2 | 7.8 | 1 | 131 |
| PPR63_ARATH | Pentatricopeptide repeat-containing protein | 36.8 (M:36.8) | 3.6 | 53.8 | 6.4 | 1 | 132 |
| PP287_ARATH | Pentatricopeptide repeat-containing protein | 36.7 (M:36.7) | 2.7 | 66.1 | 5.3 | 1 | 133 |
| IMGA\|AC202349_3.1 | Receptor protein kinase-like protein | 36.6 (M:36.6) | 2.7 | 75.0 | 9.2 | 1 | 134 |
| AMYB_TRIRP | Beta-amylase (EC 3.2.1.2) | 36.6 (M:36.6) | 4.0 | 56.1 | 4.8 | 1 | 135 |
| IMGA\|Medtr5g005100.1 | Vacuolar-sorting receptor 1 | 36.5 (M:36.5) | 4.0 | 68.7 | 5.7 | 1 | 136 |
| WDR12_CHLRE | Ribosome biogenesis protein WDR12 homolog | 36.2 (M:36.2) | 3.4 | 46.8 | 6.3 | 1 | 137 |
| IMGA\|Medtr8g070780.1 | Defensin | 36.0 (M:36.0) | 31.2 | 8.3 | 10.8 | 1 | 138 |
| GME_ARATH | GDP-mannose 3,5-epimerase (EC 5.1.3.18) | 36.0 (M:36.0) | 7.7 | 42.7 | 5.8 | 1 | 139 |
| IMGA\|Medtr3g101640.1 | Laccase-15 | 35.9 (M:35.9) | 2.9 | 125.4 | 9.8 | 1 | 140 |
| IMGA\|Medtr3g025380.1 | E3 ubiquitin-protein ligase | 35.9 (M:35.9) | 5.0 | 16.5 | 9.6 | 1 | 141 |
| NDHK_MARPO | NAD(P)H-quinone oxidoreductase chain K | 35.8 (M:35.8) | 4.5 | 27.6 | 9.6 | 1 | 142 |
| IMGA\|Medtr4g104360.1 | Eukaryotic translation initiation factor 4 gamma 2 | 35.8 (M:35.8) | 2.3 | 87.5 | 5.0 | 1 | 143 |
| IMGA\|Medtr2g088430.1 | Glutamate receptor 3.7 | 35.7 (M:35.7) | 1.5 | 102.2 | 9.0 | 1 | 144 |
| IMGA\|Medtr4g009620.1 | Unknown Protein | 35.7 (M:35.7) | 3.3 | 65.7 | 6.1 | 1 | 145 |
| PSAF_ARATH | Photosystem I reaction center subunit III | 35.6 (M:35.6) | 6.3 | 24.2 | 10.2 | 1 | 146 |
| IMGA\|Medtr4g026450.1 | Uncharacterized aarF domain-containing protein kinase | 35.6 (M:35.6) | 1.8 | 79.8 | 5.9 | 1 | 147 |
| YCF1_DIOEL | Putative membrane protein | 35.4 (M:35.4) | 1.3 | 222.8 | 10.0 | 1 | 148 |
| CALR_RICCO | Calreticulin | 35.3 (M:35.3) | 5.8 | 47.5 | 4.2 | 1 | 149 |
| RPOC1_BARVE | DNA-directed RNA polymerase subunit beta | 35.3 (M:35.3) | 1.9 | 78.5 | 9.5 | 1 | 150 |
| IMGA\|Medtr3g118040.1 | Exportin-7 | 35.2 (M:35.2) | 2.0 | 97.0 | 5.8 | 1 | 151 |
| FBT4_ARATH | Probable folate-biopterin transporter 4 | 35.1 (M:35.1) | 6.9 | 53.8 | 9.9 | 1 | 152 |
| IMGA\|Medtr8g060700.1 | Unknown Protein | 35.1 (M:35.1) | 2.6 | 84.3 | 5.1 | 1 | 153 |
| IMGA\|Medtr4g130620.1 | F-box protein SKIP1 | 35.1 (M:35.1) | 5.0 | 34.7 | 5.7 | 1 | 154 |
| IMGA\|Medtr3g092600.1 | 60S acidic ribosomal protein P1 | 35.1 (M:35.1) | 35.5 | 11.1 | 4.1 | 1 | 155 |
| IMGA\|Medtr2g104400.1 | CTV.22 | 35.1 (M:35.1) | 2.2 | 142.5 | 9.7 | 1 | 156 |
| SPL12_ARATH | Squamosa promoter-binding-like protein 12 | 35.0 (M:35.0) | 2.0 | 104.1 | 5.8 | 1 | 157 |

**M. orbicularis 22 d**

| Accession | Protein | Scores | S.Coverage | MW [kDa] | pI | Peptides | Rank |
|---|---|---|---|---|---|---|---|
| IMGA\|Medtr7g079730.1 | Convicilin (Fragment) | 1530.7 (M:1530.7) | 26.7 | 53.1 | 5.3 | 24 | 1 |
| IMGA\|Medtr1g072630.1 | Legumin B | 1329.3 (M:1329.3) | 32.0 | 65.3 | 6.1 | 22 | 2 |
| IMGA\|Medtr7g079780.1 | Convicilin (Fragment) | 1148.3 (M:1148.3) | 30.4 | 53.2 | 5.5 | 7 | 3 |
| IMGA\|Medtr1g072600.1 | Legumin B (Fragment) | 1055.7 (M:1055.7) | 30.7 | 67.2 | 6.5 | 3 | 4 |
| IMGA\|Medtr7g079770.1 | Provicilin (Fragment) | 1025.2 (M:1025.2) | 31.3 | 53.1 | 5.3 | 2 | 5 |
| IMGA\|Medtr1g072610.2 | Legumin B (Fragment) | 1015.2 (M:1015.2) | 26.8 | 63.4 | 6.4 | 2 | 6 |
| IMGA\|Medtr7g079820.1 | Convicilin (Fragment) | 916.7 (M:916.7) | 25.7 | 53.7 | 5.6 | 1 | 7 |
| IMGA\|Medtr2g099560.1 | Lipoxygenase | 584.0 (M:584.0) | 14.7 | 96.9 | 5.8 | 10 | 8 |
| VCLC_PEA | Vicilin precursor | 519.5 (M:519.5) | 14.8 | 52.2 | 5.3 | 4 | 9 |
| IMGA\|Medtr2g099570.1 | Seed lipoxygenase-3 | 502.1 (M:502.1) | 11.6 | 97.3 | 6.1 | 7 | 10 |
| IMGA\|Medtr5g019780.1 | Vicilin-like antimicrobial peptides 2-3 (Fragment) | 421.0 (M:421.0) | 15.1 | 86.4 | 5.2 | 7 | 11 |
| IMGA\|Medtr4g103920.1 | Glyceraldehyde-3-phosphate dehydrogenase (Fragment) | 411.4 (M:411.4) | 30.4 | 36.6 | 6.7 | 6 | 12 |
| IMGA\|Medtr1g108770.2 | ATP synthase subunit beta | 393.9 (M:393.9) | 15.6 | 59.9 | 5.8 | 6 | 13 |
| IMGA\|Medtr5g096430.1 | Heat shock protein 90 (Fragment) | 378.4 (M:378.4) | 15.3 | 80.1 | 4.8 | 7 | 14 |
| IMGA\|Medtr4g051270.1 | Ribulose bisphosphate carboxylase large chain | 372.5 (M:372.5) | 13.8 | 50.6 | 6.2 | 6 | 15 |
| EF1A_MAIZE | Elongation factor 1-alpha | 370.5 (M:370.5) | 16.1 | 49.2 | 9.8 | 7 | 16 |
| IMGA\|Medtr7g024580.1 | Heat shock protein 70 | 346.5 (M:346.5) | 12.8 | 71.0 | 4.9 | 5 | 17 |
| IMGA\|Medtr2g083160.1 | Conglutin | 346.4 (M:346.4) | 12.1 | 16.8 | 6.3 | 5 | 18 |
| IMGA\|Medtr1g071710.1 | Vicilin-like antimicrobial peptides 2-2 | 311.6 (M:311.6) | 11.1 | 56.0 | 6.0 | 5 | 19 |
| IMGA\|Medtr7g086300.1 | Methionine synthase | 283.8 (M:283.8) | 13.6 | 88.8 | 6.2 | 5 | 20 |
| IMGA\|Medtr7g113650.1 | Glucose and ribitol dehydrogenase homolog 1 | 280.9 (M:280.9) | 22.5 | 31.8 | 7.7 | 5 | 21 |
| HSP70_MAIZE | Heat shock 70 kDa protein | 274.7 (M:274.7) | 9.0 | 70.5 | 5.1 | 1 | 22 |
| IMGA\|Medtr2g066120.1 | Phosphoglycerate kinase | 271.7 (M:271.7) | 17.5 | 42.4 | 5.7 | 4 | 23 |
| IMGA\|Medtr4g060780.1 | Allergen Gly m Bd (Fragment) | 266.4 (M:266.4) | 11.2 | 52.8 | 5.7 | 5 | 24 |
| IMGA\|Medtr1g043040.1 | Malate dehydrogenase | 249.9 (M:249.9) | 13.6 | 35.5 | 6.1 | 4 | 25 |
| IMGA\|Medtr4g124660.2 | Sucrose synthase | 234.5 (M:234.5) | 8.0 | 92.2 | 5.8 | 5 | 26 |
| ENO_RICCO | Enolase (EC 4.2.1.11) | 229.3 (M:229.3) | 9.9 | 47.9 | 5.5 | 3 | 27 |
| ATPAM_PEA | ATP synthase subunit alpha, mitochondrial | 224.5 (M:224.5) | 10.5 | 55.0 | 6.0 | 4 | 28 |
| IMGA\|Medtr7g113790.1 | Basic 7S globulin 2 | 218.2 (M:218.2) | 7.1 | 48.1 | 10.0 | 3 | 29 |
| IMGA\|Medtr3g098420.1 | Staphylococcal nuclease domain-containing protein | 210.5 (M:210.5) | 8.0 | 108.2 | 6.6 | 4 | 30 |
| ALFC1_PEA | Fructose-bisphosphate aldolase 1, chloroplastic | 208.8 (M:208.8) | 18.0 | 38.6 | 5.8 | 4 | 31 |
| BIP_SPIOL | Luminal-binding protein precursor (BiP) | 204.7 (M:204.7) | 6.7 | 73.5 | 4.9 | 2 | 32 |
| IMGA\|Medtr3g085850.1 | Glyceraldehyde 3-phosphate dehydrogenase | 201.1 (M:201.1) | 12.6 | 37.0 | 7.7 | 1 | 33 |
| IMGA\|Medtr2g029730.1 | Peroxidase | 198.8 (M:198.8) | 4.3 | 37.4 | 6.2 | 2 | 34 |
| IMGA\|Medtr8g085980.1 | Alpha-tubulin | 195.2 (M:195.2) | 14.7 | 49.5 | 4.9 | 3 | 35 |
| IMGA\|Medtr2g005570.2 | Elongation factor 1-gamma | 188.5 (M:188.5) | 12.7 | 47.7 | 7.7 | 3 | 36 |
| ENO_SOLLC | Enolase | 178.8 (M:178.8) | 9.0 | 47.8 | 5.6 | 1 | 37 |
| G3PC2_ARATH | Glyceraldehyde-3-phosphate dehydrogenase | 173.5 (M:173.5) | 13.9 | 36.9 | 6.8 | 1 | 38 |
| PSBB_CICAR | Photosystem II CP47 | 171.5 (M:171.5) | 8.1 | 56.0 | 6.3 | 3 | 39 |
| IMGA\|Medtr1g083960.1 | Calreticulin | 169.1 (M:169.1) | 12.3 | 48.4 | 4.3 | 3 | 40 |
| PDI_MEDSA | Protein disulfide-isomerase precursor | 166.4 (M:166.4) | 7.0 | 57.1 | 4.8 | 3 | 41 |
| METE_SOLSC | 5-methyltetrahydropteroyltriglutamate--homocysteine methyltransferase | 138.6 (M:138.6) | 7.1 | 84.5 | 6.1 | 1 | 42 |
| CALR_NICPL | Calreticulin | 134.8 (M:134.8) | 13.2 | 47.5 | 4.3 | 1 | 43 |
| IMGA\|Medtr5g074860.2 | Peroxidase | 133.7 (M:133.7) | 8.4 | 31.4 | 10.4 | 2 | 44 |
| G3PA_TOBAC | Glyceraldehyde-3-phosphate dehydrogenase A | 121.4 (M:121.4) | 10.5 | 41.8 | 6.7 | 2 | 45 |
| LEG_CICAR | Legumin | 119.8 (M:119.8) | 2.4 | 56.2 | 6.2 | 2 | 46 |
| IMGA\|Medtr5g069050.1 | Fructose-bisphosphate aldolase | 118.8 (M:118.8) | 7.7 | 78.3 | 5.7 | 2 | 47 |
| ILV5_PEA | Ketol-acid reductoisomerase | 118.0 (M:118.0) | 5.3 | 62.8 | 6.7 | 2 | 48 |
| LEGJ_PEA | Legumin J precursor | 114.7 (M:114.7) | 5.4 | 56.9 | 5.6 | 2 | 49 |
| IMGA\|Medtr2g069050.1 | Elongation factor EF-2 (Fragment) | 113.4 (M:113.4) | 5.6 | 94.1 | 5.7 | 3 | 50 |
| PDIA6_MEDSA | Probable protein disulfide-isomerase A6 precursor | 113.3 (M:113.3) | 9.9 | 40.5 | 5.3 | 2 | 51 |
| IMGA\|Medtr6g018300.1 | Ribulose bisphosphate carboxylase small chain (Fragment) | 111.0 (M:111.0) | 13.0 | 19.8 | 9.4 | 2 | 52 |
| GBLPA_ORYSJ | Guanine nucleotide-binding protein subunit beta-like protein A | 108.3 (M:108.3) | 8.1 | 36.2 | 6.0 | 1 | 53 |
| IMGA\|Medtr7g009330.1 | Outer plastidial membrane protein porin | 108.2 (M:108.2) | 11.9 | 29.6 | 9.4 | 2 | 54 |
| ATPB_MEDSA | ATP synthase subunit beta, chloroplastic | 104.5 (M:104.5) | 9.6 | 52.7 | 5.0 | 2 | 55 |
| IMGA\|Medtr4g076100.1 | Aminotransferase | 103.4 (M:103.4) | 4.5 | 56.5 | 8.5 | 2 | 56 |
| UBIQ_AVESA | Ubiquitin | 103.2 (M:103.2) | 32.9 | 8.5 | 7.6 | 2 | 57 |
| IMGA\|Medtr1g025430.1 | Endoplasmin homolog | 103.1 (M:103.1) | 3.2 | 94.1 | 4.6 | 2 | 58 |
| IMGA\|Medtr5g064580.1 | 14-3-3 protein (Fragment) | 103.1 (M:103.1) | 5.8 | 29.2 | 4.5 | 1 | 59 |
| IMGA\|Medtr6g052140.1 | 40S ribosomal protein S3 | 102.5 (M:102.5) | 9.4 | 26.2 | 10.4 | 2 | 60 |
| WDR12_CHLRE | Ribosome biogenesis protein WDR12 homolog | 101.7 (M:101.7) | 3.4 | 46.8 | 6.3 | 2 | 61 |
| IMGA\|Medtr4g130860.1 | Leucine aminopeptidase 2 | 98.2 (M:98.2) | 5.3 | 59.6 | 8.6 | 2 | 62 |
| IMGA\|Medtr2g038250.1 | 60S ribosomal protein L7-4 | 97.0 (M:97.0) | 6.1 | 28.5 | 10.4 | 1 | 63 |
| IMGA\|AC235488_13.1 | 40S ribosomal protein S5 | 97.0 (M:97.0) | 11.3 | 27.9 | 9.8 | 2 | 64 |
| IMGA\|Medtr2g098010.1 | Proteasome subunit beta type | 96.3 (M:96.3) | 20.8 | 25.4 | 6.3 | 2 | 65 |
| RL37A_GOSHI | 60S ribosomal protein L37a | 96.2 (M:96.2) | 17.4 | 10.2 | 11.3 | 1 | 66 |
| IMGA\|AC146630_2.1 | 2-cys peroxiredoxin BAS1 | 93.4 (M:93.4) | 14.7 | 29.0 | 6.1 | 2 | 67 |
| IMGA\|Medtr7g083560.1 | Translocon-associated protein subunit beta | 93.2 (M:93.2) | 20.6 | 21.0 | 9.9 | 2 | 68 |
| IMGA\|Medtr2g014030.1 | 40S ribosomal protein S6 | 92.8 (M:92.8) | 6.0 | 28.2 | 11.5 | 1 | 69 |
| IMGA\|Medtr4g034980.1 | Photosystem II CP43 chlorophyll apoprotein | 91.6 (M:91.6) | 3.1 | 81.0 | 6.4 | 2 | 70 |
| ACT2_SOLTU | Actin-46 | 87.6 (M:87.6) | 4.8 | 37.1 | 5.2 | 1 | 71 |
| IMGA\|Medtr8g081490.1 | Adenosylhomocysteinase | 86.8 (M:86.8) | 7.9 | 49.8 | 5.2 | 2 | 72 |
| ACLB1_ARATH | ATP-citrate synthase beta chain protein 1 | 86.8 (M:86.8) | 5.4 | 65.8 | 8.8 | 2 | 73 |
| IMGA\|Medtr6g005820.3 | ADP-ribosylation factor | 86.5 (M:86.5) | 22.0 | 18.7 | 5.5 | 2 | 74 |
| CPNA1_ARATH | Chaperonin 60 subunit alpha 1 | 85.8 (M:85.8) | 4.3 | 62.0 | 4.9 | 2 | 75 |
| CHI4_SOLTU | Endochitinase 4 (Fragment) | 84.2 (M:84.2) | 7.3 | 32.3 | 9.8 | 1 | 76 |
| IMGA\|Medtr4g015570.1 | Chlorophyll a-b binding protein, chloroplastic | 82.9 (M:82.9) | 8.4 | 38.0 | 8.8 | 2 | 77 |
| IMGA\|Medtr4g050400.1 | Unknown Protein (AHRD V1) | 82.8 (M:82.8) | 13.0 | 16.4 | 9.0 | 2 | 78 |
| IMGA\|Medtr3g100220.1 | Argininosuccinate lyase | 81.1 (M:81.1) | 3.8 | 52.4 | 5.6 | 1 | 79 |
| SODM_PRUPE | Superoxide dismutase [Mn], mitochondrial precursor (EC 1.15.1.1) | 80.3 (M:80.3) | 6.6 | 25.4 | 9.1 | 1 | 80 |
| IMGA\|Medtr7g092720.1 | 40S ribosomal protein S20-2 | 78.5 (M:78.5) | 9.8 | 13.7 | 10.2 | 1 | 81 |

| | | | | | | |
|---|---|---|---|---|---|---|
| IMGA\|Medtr8g058480.1 | Zinc finger CCCH domain-containing protein 51 | 78.4 (M:78.4) | 7.0 | 39.0 | 4.7 | 1 | 82 |
| IMGA\|Medtr8g095680.1 | Calnexin homolog | 78.4 (M:78.4) | 3.7 | 61.8 | 4.7 | 2 | 83 |
| IMGA\|Medtr8g046140.2 | 60S ribosomal protein L11 | 78.0 (M:78.0) | 12.0 | 13.2 | 10.4 | 1 | 84 |
| IMGA\|Medtr1g064060.2 | Adenosine kinase 2 | 76.9 (M:76.9) | 5.7 | 35.1 | 5.6 | 1 | 85 |
| RNH2A_ARATH | Ribonuclease H2 subunit A | 75.6 (M:75.6) | 5.4 | 33.1 | 6.3 | 2 | 86 |
| ACT3_SOLLC | Actin-52 | 75.1 (M:75.1) | 4.8 | 37.1 | 5.4 | 1 | 87 |
| IMGA\|Medtr2g076490.1 | At1g78850-like protein (Fragment) | 74.6 (M:74.6) | 4.9 | 55.0 | 6.4 | 1 | 88 |
| IMGA\|Medtr3g087590.3 | L-myo inositol-1 phosphate synthase 1 | 73.8 (M:73.8) | 5.8 | 47.3 | 5.3 | 1 | 89 |
| IMGA\|Medtr4g070080.2 | Glycine-rich RNA-binding protein | 73.3 (M:73.3) | 23.5 | 5.7 | 4.3 | 1 | 90 |
| TBB3_SOYBN | Tubulin beta chain (Beta tubulin) (Fragment) | 73.3 (M:73.3) | 6.4 | 45.7 | 5.6 | 1 | 91 |
| DIOX3_PAPSO | Codeine O-demethylase | 71.6 (M:71.6) | 6.1 | 40.8 | 4.9 | 2 | 92 |
| PSBQ_ONOVI | Oxygen-evolving enhancer protein 3 | 71.5 (M:71.5) | 5.6 | 24.8 | 10.0 | 1 | 93 |
| IMGA\|Medtr1g081410.1 | 40S ribosomal protein S24 | 69.3 (M:69.3) | 10.9 | 15.7 | 11.1 | 1 | 94 |
| RL24_HORVU | 60S ribosomal protein L24 | 68.8 (M:68.8) | 7.4 | 18.4 | 11.4 | 1 | 95 |
| IMGA\|Medtr3g093110.1 | Ribosomal protein L9 (Fragment) | 68.2 (M:68.2) | 6.2 | 21.7 | 9.9 | 1 | 96 |
| IMGA\|Medtr1g075320.1 | N-carbamoyl-L-amino acid hydrolase | 66.8 (M:66.8) | 2.6 | 54.1 | 5.6 | 1 | 97 |
| IMGA\|Medtr3g089970.3 | Alcohol dehydrogenase 1 (Fragment) | 66.1 (M:66.1) | 4.7 | 23.1 | 7.8 | 1 | 98 |
| IMGA\|Medtr4g049550.1 | Disease resistance response protein 206 | 65.6 (M:65.6) | 6.4 | 25.8 | 9.7 | 1 | 99 |
| IMGA\|Medtr1g094630.1 | 60S ribosomal protein L4 | 63.1 (M:63.1) | 1.7 | 130.9 | 6.5 | 1 | 100 |
| IMGA\|Medtr4g090220.1 | 6-phosphogluconate dehydrogenase, decarboxylating | 63.0 (M:63.0) | 7.8 | 20.7 | 6.2 | 1 | 101 |
| IMGA\|Medtr5g033920.1 | ATP-citrate synthase | 62.8 (M:62.8) | 5.7 | 46.6 | 5.5 | 1 | 102 |
| RS271_ARATH | 40S ribosomal protein S27-1 | 62.7 (M:62.7) | 20.2 | 9.4 | 10.6 | 1 | 103 |
| IMGA\|Medtr4g061140.1 | Cytosolic ascorbate peroxidase | 61.8 (M:61.8) | 7.2 | 27.1 | 5.5 | 1 | 104 |
| SUS2_HORVU | Sucrose synthase 2 (EC 2.4.1.13) | 61.2 (M:61.2) | 2.6 | 92.5 | 6.3 | 1 | 105 |
| IMGA\|Medtr2g042330.2 | Aldehyde dehydrogenase family 7 member A1 | 60.7 (M:60.7) | 5.1 | 54.2 | 5.8 | 1 | 106 |
| IMGA\|AC235753_1.1 | Cysteine proteinase | 60.5 (M:60.5) | 3.9 | 40.3 | 6.1 | 1 | 107 |
| IMGA\|Medtr4g103800.1 | Adenylate kinase B | 59.3 (M:59.3) | 6.2 | 26.7 | 8.8 | 1 | 108 |
| IMGA\|Medtr4g063060.1 | 60S ribosomal protein L23a | 59.2 (M:59.2) | 8.6 | 17.1 | 10.7 | 1 | 109 |
| ENO_ORYSJ | Enolase | 58.6 (M:58.6) | 5.6 | 47.9 | 5.3 | 1 | 110 |
| IMGA\|Medtr6g005040.1 | Oleosin 1 | 58.4 (M:58.4) | 5.7 | 20.7 | 9.3 | 1 | 111 |
| PSBP1_ARATH | Oxygen-evolving enhancer protein 2-1 | 58.2 (M:58.2) | 5.3 | 28.1 | 7.7 | 1 | 112 |
| IMGA\|Medtr7g050870.1 | Pectinesterase | 58.1 (M:58.1) | 3.1 | 60.5 | 9.6 | 1 | 113 |
| GSA_SOYBN | Glutamate-1-semialdehyde 2,1-aminomutase | 58.1 (M:58.1) | 4.5 | 49.6 | 5.6 | 1 | 114 |
| IMGA\|Medtr4g115970.2 | Vacuolar proton-inorganic pyrophosphatase | 58.0 (M:58.0) | 3.1 | 75.6 | 4.9 | 1 | 115 |
| IMGA\|Medtr8g067080.1 | ADP-ribosylation factor GTPase-activating protein | 57.5 (M:57.5) | 6.6 | 43.9 | 9.7 | 1 | 116 |
| IMGA\|Medtr3g088450.1 | Photosystem Q(B) protein | 56.8 (M:56.8) | 9.6 | 12.8 | 10.5 | 1 | 117 |
| IMGA\|Medtr1g066380.2 | Peroxidase | 56.6 (M:56.6) | 6.6 | 28.5 | 6.8 | 1 | 118 |
| AGAL_CAPAA | Alpha-galactosidase (Fragment) | 55.8 (M:55.8) | 100.0 | 1.4 | 6.7 | 1 | 119 |
| IMGA\|Medtr8g076210.1 | Thymus-specific serine protease | 55.7 (M:55.7) | 2.9 | 51.0 | 5.0 | 1 | 120 |
| IMGA\|Medtr8g072000.4 | Unknown Protein | 55.5 (M:55.5) | 22.1 | 9.8 | 7.7 | 1 | 121 |
| ADT_CHLRE | ADP,ATP carrier protein (ADP/ATP translocase) | 55.2 (M:55.2) | 3.6 | 33.5 | 10.2 | 1 | 122 |
| IMGA\|Medtr2g025120.1 | 1-aminocyclopropane-1-carboxylate oxidase (Fragment) | 55.1 (M:55.1) | 10.4 | 36.1 | 4.9 | 1 | 123 |
| IMGA\|Medtr4g059400.1 | 60S ribosomal protein L12 | 54.7 (M:54.7) | 9.0 | 17.8 | 9.7 | 1 | 124 |
| IMGA\|Medtr1g045410.1 | 60S ribosomal protein L4 | 53.6 (M:53.6) | 3.0 | 44.7 | 10.9 | 1 | 125 |
| IMGA\|Medtr1g090130.1 | Chaperonin CPN60-2, mitochondrial | 53.5 (M:53.5) | 4.2 | 61.1 | 6.3 | 1 | 126 |
| PGMC_POPTN | Phosphoglucomutase, cytoplasmic (EC 5.4.2.2) | 52.6 (M:52.6) | 4.0 | 63.1 | 5.4 | 1 | 127 |
| IMGA\|Medtr3g077050.1 | 60S ribosomal protein L27a-3 | 52.1 (M:52.1) | 6.8 | 16.6 | 11.2 | 1 | 128 |
| IMGA\|Medtr1g106900.2 | 40S ribosomal protein S18 | 51.9 (M:51.9) | 14.1 | 10.2 | 11.9 | 1 | 129 |
| EF1B_ORYSJ | Elongation factor 1-beta | 51.4 (M:51.4) | 7.6 | 23.8 | 4.5 | 1 | 130 |
| IMGA\|Medtr3g118030.1 | 60S ribosomal protein L5 | 50.4 (M:50.4) | 2.7 | 37.5 | 9.6 | 1 | 131 |
| IMGA\|Medtr7g072000.4 | Alpha-soluble NSF attachment protein | 50.3 (M:50.3) | 11.1 | 13.9 | 10.2 | 1 | 132 |
| CDC48_CAPAN | Cell division cycle protein 48 homolog | 50.3 (M:50.3) | 2.6 | 89.3 | 4.9 | 1 | 133 |
| IMGA\|Medtr4g013100.1 | Glyoxylate reductase | 50.0 (M:50.0) | 6.4 | 34.3 | 6.0 | 1 | 134 |
| TKTC_CRAPL | Transketolase, chloroplastic (Fragment) | 49.8 (M:49.8) | 4.0 | 56.2 | 5.8 | 1 | 135 |
| IMGA\|Medtr3g064480.1 | UTP--glucose-1-phosphate uridylyltransferase | 49.8 (M:49.8) | 6.4 | 26.1 | -1.6 | 1 | 136 |
| IMGA\|Medtr5g091930.1 | Citrate synthase | 49.4 (M:49.4) | 2.7 | 56.6 | 9.1 | 1 | 137 |
| IMGA\|Medtr3g104930.1 | Subtilisin-type protease | 49.2 (M:49.2) | 1.4 | 81.0 | 6.1 | 1 | 138 |
| IMGA\|Medtr7g026030.1 | Heat shock protein 70 (Fragment) | 48.8 (M:48.8) | 1.9 | 69.6 | 5.3 | 1 | 139 |
| IMGA\|Medtr4g107040.1 | Mitochondrial aldehyde dehydrogenase | 48.3 (M:48.3) | 2.4 | 59.1 | 9.1 | 1 | 140 |
| IMGA\|Medtr7g021030.1 | 60S ribosomal protein L30 | 47.5 (M:47.5) | 14.3 | 12.3 | 10.2 | 1 | 141 |
| IMGA\|Medtr2g095750.1 | Serine carboxypeptidase | 47.3 (M:47.3) | 3.1 | 57.3 | 5.2 | 1 | 142 |
| H2A3_VOLCA | Histone H2A-III | 47.2 (M:47.2) | 7.0 | 13.5 | 10.7 | 1 | 143 |
| IMGA\|Medtr2g096660.1 | UDP-glucuronic acid decarboxylase 3 | 47.1 (M:47.1) | 8.0 | 39.6 | 6.9 | 1 | 144 |
| NDK1_PSEMZ | Nucleoside diphosphate kinase 1 (Fragments) | 47.1 (M:47.1) | 50.0 | 1.9 | 4.4 | 1 | 145 |
| IMGA\|Medtr5g062540.1 | Beta-glucosidase (Fragment) | 46.8 (M:46.8) | 3.0 | 39.5 | 6.0 | 1 | 146 |
| IMGA\|Medtr5g018940.1 | 40S ribosomal protein S4 | 45.9 (M:45.9) | 3.4 | 29.9 | 10.8 | 1 | 147 |
| RLA01_ARATH | 60S acidic ribosomal protein P0-1 | 45.8 (M:45.8) | 4.1 | 33.6 | 5.0 | 1 | 148 |
| IMGA\|Medtr4g075340.1 | Translocon-associated protein subunit alpha | 45.8 (M:45.8) | 5.1 | 27.5 | 4.6 | 1 | 149 |
| IMGA\|Medtr2g064660.2 | Photosystem II 10 kDa polypeptide, chloroplastic | 45.4 (M:45.4) | 11.8 | 13.6 | 10.2 | 1 | 150 |
| IMGA\|Medtr4g112010.1 | Potassium channel | 45.2 (M:45.2) | 2.9 | 63.4 | 10.3 | 1 | 151 |
| IMGA\|Medtr2g098520.1 | Beta-glucosidase D4 | 45.0 (M:45.0) | 4.6 | 58.2 | 9.3 | 1 | 152 |
| IMGA\|Medtr2g033930.1 | GDSL esterase/lipase | 44.7 (M:44.7) | 4.4 | 40.0 | 7.6 | 1 | 153 |
| IMGA\|AC235665_13.2 | 40S ribosomal protein S3a | 44.6 (M:44.6) | 8.8 | 18.4 | 10.1 | 1 | 154 |
| IMGA\|Medtr6g071760.1 | Unknown Protein | 44.2 (M:44.2) | 3.8 | 71.5 | 9.3 | 1 | 155 |
| IMGA\|Medtr7g101070.1 | DnaJ homolog subfamily C member 3 homolog | 44.0 (M:44.0) | 2.9 | 52.7 | 6.3 | 1 | 156 |
| IMGA\|Medtr7g113470.1 | T-complex protein 1 subunit beta | 43.8 (M:43.8) | 4.2 | 56.9 | 5.3 | 1 | 157 |
| IMGA\|Medtr1g075790.1 | Os12g0236050 protein (Fragment) | 43.8 (M:43.8) | 1.9 | 111.0 | 5.0 | 1 | 158 |
| IMGA\|Medtr1g088450.1 | 60S ribosomal protein L22-like | 43.8 (M:43.8) | 7.6 | 13.6 | 10.0 | 1 | 159 |
| EF1D1_ARATH | Elongation factor 1-delta 1 (EF-1-delta 1) | 43.8 (M:43.8) | 5.6 | 25.1 | 4.3 | 1 | 160 |
| IMGA\|Medtr1g079530.1 | GDSL esterase/lipase | 43.6 (M:43.6) | 3.6 | 39.6 | 9.4 | 1 | 161 |
| IMGA\|Medtr2g018290.2 | Serine hydroxymethyltransferase | 43.4 (M:43.4) | 7.2 | 39.0 | 6.8 | 1 | 162 |
| Y4634_ARATH | Uncharacterized zinc finger protein | 43.3 (M:43.3) | 5.2 | 44.7 | 6.0 | 1 | 163 |
| IMGA\|Medtr3g104720.1 | Puromycin-sensitive aminopeptidase | 43.2 (M:43.2) | 1.5 | 98.9 | 5.4 | 1 | 164 |
| IMGA\|Medtr2g039680.1 | Nucleosome assembly protein 1-like 1 | 43.2 (M:43.2) | 4.9 | 41.8 | 4.1 | 1 | 165 |

| | | | | | | | |
|---|---|---|---|---|---|---|---|
| UN03_PINPS | Unknown protein | 43.1 (M:43.1) | 34.0 | 5.8 | 4.2 | 1 | 166 |
| RFC1_ARATH | Replication factor C subunit 1 | 43.0 (M:43.0) | 1.4 | 104.3 | 9.9 | 1 | 167 |
| EXS_ARATH | Leucine-rich repeat receptor protein kinase | 42.8 (M:42.8) | 1.7 | 129.7 | 5.5 | 1 | 168 |
| IMGA\|Medtr5g080770.1 | Mannose-1-phosphate guanyltransferase | 42.5 (M:42.5) | 5.0 | 39.5 | 6.5 | 1 | 169 |
| IMGA\|Medtr2g030240.1 | 2-succinylbenzoate--CoA ligase | 42.4 (M:42.4) | 3.7 | 63.3 | 8.0 | 1 | 170 |
| IMGA\|Medtr8g091850.1 | Transcription factor tfiiib component | 42.3 (M:42.3) | 3.1 | 34.5 | 10.0 | 1 | 171 |
| DRB7_ORYSJ | Double-stranded RNA-binding protein7 | 42.2 (M:42.2) | 3.6 | 51.2 | 4.9 | 1 | 172 |
| IMGA\|Medtr3g025380.1 | E3 ubiquitin-protein ligase RNF181 | 42.0 (M:42.0) | 5.0 | 16.5 | 9.6 | 1 | 173 |
| IMGA\|Medtr6g084400.1 | Plant synaptotagmin | 42.0 (M:42.0) | 0.8 | 93.3 | 6.8 | 1 | 174 |
| GH34_ARATH | Indole-3-acetic acid-amido synthetase GH3.4 (EC 6.3.2.-) | 42.0 (M:42.0) | 3.5 | 67.0 | 5.3 | 1 | 175 |
| IMGA\|Medtr8g031830.1 | Ras-related protein Rab-2-A | 41.4 (M:41.4) | 8.6 | 22.6 | 5.6 | 1 | 176 |
| IMGA\|Medtr2g015560.1 | Pyruvate decarboxylase | 41.3 (M:41.3) | 3.4 | 63.1 | 5.6 | 1 | 177 |
| P2C09_ORYSJ | Probable protein phosphatase 2C | 41.2 (M:41.2) | 4.1 | 44.0 | 5.3 | 1 | 178 |
| IMGA\|Medtr2g034690.1 | Unknown Protein | 41.1 (M:41.1) | 5.3 | 21.4 | 10.1 | 1 | 179 |
| IMGA\|Medtr3g088940.1 | Argininosuccinate synthase | 41.1 (M:41.1) | 10.5 | 16.6 | 7.6 | 1 | 180 |
| IMGA\|Medtr7g118060.1 | 60s acidic ribosomal protein P1 | 41.0 (M:41.0) | 32.7 | 11.4 | 4.0 | 1 | 181 |
| IMGA\|Medtr4g079700.2 | Unknown Protein | 40.5 (M:40.5) | 4.4 | 31.6 | 9.6 | 1 | 182 |
| RLA31_ARATH | 60S acidic ribosomal protein P3-1 | 40.4 (M:40.4) | 15.1 | 11.8 | 4.3 | 1 | 183 |
| IMGA\|Medtr2g034720.1 | Beta xylosidase | 40.4 (M:40.4) | 2.4 | 82.6 | 9.6 | 1 | 184 |
| G6PI_SPIOL | Glucose-6-phosphate isomerase | 40.2 (M:40.2) | 2.1 | 62.1 | 6.4 | 1 | 185 |
| IMGA\|Medtr1g025610.1 | Vacuolar protein sorting 35 | 40.0 (M:40.0) | 2.5 | 89.4 | 5.2 | 1 | 186 |
| IMGA\|Medtr2g013640.1 | Unknown Protein | 40.0 (M:40.0) | 1.0 | 106.3 | 9.8 | 1 | 187 |
| RS10_ORYSJ | 40S ribosomal protein S10 | 39.8 (M:39.8) | 7.1 | 20.3 | 10.2 | 1 | 188 |
| IF415_TOBAC | Eukaryotic initiation factor 4A-15 | 39.8 (M:39.8) | 3.6 | 46.7 | 5.3 | 1 | 189 |
| IMGA\|Medtr4g061960.1 | CCR4-NOT transcription complex subunit 2 | 39.6 (M:39.6) | 4.8 | 37.7 | 6.2 | 1 | 190 |
| IMGA\|Medtr7g022750.1 | Unknown Protein | 39.6 (M:39.6) | 14.9 | 15.2 | 9.9 | 1 | 191 |
| IMGA\|Medtr1g008240.1 | Cc-nbs resistance protein (Fragment) | 39.5 (M:39.5) | 1.8 | 143.6 | 6.2 | 1 | 192 |
| IMGA\|Medtr8g076910.1 | Tir-nbs-lrr resistance protein (Fragment) | 39.3 (M:39.3) | 1.1 | 126.6 | 6.2 | 1 | 193 |
| FBD1_ARATH | Putative FBD-associated F-box protein | 39.1 (M:39.1) | 3.2 | 42.4 | 10.1 | 1 | 194 |
| SYM_ORYSJ | Probable methionyl-tRNA synthetase | 39.0 (M:39.0) | 2.0 | 89.4 | 6.6 | 1 | 195 |
| PDC2_TOBAC | Pyruvate decarboxylase isozyme 2 | 39.0 (M:39.0) | 4.2 | 67.0 | 5.6 | 1 | 196 |
| RL7A2_ARATH | 60S ribosomal protein L7a-2 | 39.0 (M:39.0) | 4.7 | 29.0 | 10.7 | 1 | 197 |
| C85A3_SOLLC | Cytochrome P450 | 38.8 (M:38.8) | 1.7 | 53.8 | 9.7 | 1 | 198 |
| IMGA\|Medtr6g065270.1 | Arylacetamide deacetylase-like 2 | 38.8 (M:38.8) | 7.0 | 38.1 | 6.0 | 1 | 199 |
| IMGA\|Medtr3g079780.1 | Tir-nbs-lrr resistance protein (Fragment) | 38.7 (M:38.7) | 1.7 | 176.6 | 5.8 | 1 | 200 |
| IMGA\|Medtr7g081700.1 | Ras-like protein (Fragment) | 38.6 (M:38.6) | 8.4 | 22.5 | 4.8 | 1 | 201 |
| YCF1_CUCSA | Putative membrane protein | 38.4 (M:38.4) | 1.4 | 220.8 | 10.2 | 1 | 202 |
| CEMA_MESVI | Chloroplast envelope membrane protein | 38.4 (M:38.4) | 7.2 | 27.9 | 5.2 | 1 | 203 |
| SPT52_ARATH | Putative transcription elongation factor SPT5 homolog 2 | 38.3 (M:38.3) | 1.2 | 110.2 | 6.0 | 1 | 204 |
| IMGA\|Medtr3g070210.2 | Aquaporin protein PIP11 | 38.2 (M:38.2) | 8.8 | 23.0 | 9.2 | 1 | 205 |
| DRL12_ARATH | Probable disease resistance protein | 38.1 (M:38.1) | 2.3 | 101.0 | 9.2 | 1 | 206 |
| IMGA\|Medtr8g077830.1 | Unknown Protein | 38.0 (M:38.0) | 1.2 | 53.2 | 6.2 | 1 | 207 |
| IMGA\|Medtr1g044720.1 | Pre-mRNA-processing factor 19 homolog 2 | 38.0 (M:38.0) | 4.2 | 56.3 | 6.2 | 1 | 208 |
| PRR73_ORYSJ | Two-component response regulator-like PRR73 | 37.9 (M:37.9) | 1.8 | 84.0 | 6.0 | 1 | 209 |
| IMGA\|Medtr6g006990.6 | Carbonic anhydrase | 37.8 (M:37.8) | 11.9 | 15.4 | 6.5 | 1 | 210 |
| C3H24_ORYSJ | Zinc finger CCCH domain-containing protein 24 | 37.7 (M:37.7) | 3.5 | 81.0 | 6.7 | 1 | 211 |
| MDAR_PEA | Monodehydroascorbate reductase | 37.6 (M:37.6) | 5.5 | 47.3 | 5.7 | 1 | 212 |
| IMGA\|Medtr5g047620.1 | Lysine-specific demethylase 3B | 37.6 (M:37.6) | 2.3 | 95.6 | 8.9 | 1 | 213 |
| IMGA\|Medtr4g014720.1 | Disease resistance protein-like | 37.5 (M:37.5) | 3.1 | 79.6 | 9.4 | 1 | 214 |
| IMGA\|Medtr5g020710.1 | Genomic DNA chromosome 3 | 37.5 (M:37.5) | 10.1 | 28.0 | 10.2 | 1 | 215 |
| IMGA\|Medtr8g103380.1 | Pentatricopeptide repeat-containing protein | 37.3 (M:37.3) | 2.5 | 75.5 | 9.6 | 1 | 216 |
| IMGA\|Medtr3g102170.1 | Kinesin-like protein | 37.3 (M:37.3) | 2.1 | 117.0 | 9.6 | 1 | 217 |
| PP201_ARATH | Pentatricopeptide repeat-containing protein | 37.2 (M:37.2) | 3.4 | 63.2 | 6.4 | 1 | 218 |
| Y1684_ARATH | Probable LRR receptor-like serine/threonine-protein kinase | 37.2 (M:37.2) | 2.2 | 105.6 | 6.4 | 1 | 219 |
| IAA31_ORYSJ | Auxin-responsive protein IAA31 | 37.1 (M:37.1) | 13.2 | 20.9 | -1.1 | 1 | 220 |
| PSBO_PEA | Oxygen-evolving enhancer protein 1 | 37.0 (M:37.0) | 7.3 | 34.9 | 6.3 | 1 | 221 |
| IMGA\|Medtr1g012540.1 | N-acetyl-gamma-glutamyl-phosphate reductase | 36.6 (M:36.6) | 4.2 | 42.2 | 7.9 | 1 | 222 |
| RPOC2_ATRBE | DNA-directed RNA polymerase beta'' chain (EC 2.7.7.6) (PEP) | 36.6 (M:36.6) | 1.3 | 156.8 | 9.9 | 1 | 223 |
| IMGA\|Medtr5g039600.1 | TCP family transcription factor-like | 36.5 (M:36.5) | 5.5 | 44.2 | 6.5 | 1 | 224 |
| IMGA\|Medtr2g101500.1 | Auxin-responsive protein IAA27 | 36.5 (M:36.5) | 6.4 | 35.3 | 9.1 | 1 | 225 |
| IMGA\|Medtr4g130680.2 | ATP phosphoribosyltransferase | 36.4 (M:36.4) | 8.3 | 23.3 | 9.3 | 1 | 226 |
| AB14C_ARATH | ABC transporter C family member 14 | 36.3 (M:36.3) | 1.2 | 172.0 | 9.5 | 1 | 227 |
| IMGA\|Medtr3g101910.1 | N-hydroxycinnamoyl/benzoyltransferase 1 | 36.3 (M:36.3) | 2.7 | 84.4 | 6.0 | 1 | 228 |
| ORP1A_ARATH | Oxysterol-binding protein-related protein 1A | 36.3 (M:36.3) | 2.5 | 87.3 | 6.3 | 1 | 229 |
| PABP8_ARATH | Polyadenylate-binding protein 8 | 36.2 (M:36.2) | 2.8 | 72.7 | 7.8 | 1 | 230 |
| IMGA\|Medtr2g083330.1 | Homeodomain protein (HB2) (Fragment) | 36.1 (M:36.1) | 2.2 | 61.1 | 6.3 | 1 | 231 |
| CESA2_ARATH | Cellulose synthase A catalytic subunit 2 | 36.1 (M:36.1) | 3.0 | 122.0 | 8.3 | 1 | 232 |
| IMGA\|Medtr7g114730.1 | DNA-damage-repair/toleration protein DRT102 | 36.0 (M:36.0) | 6.4 | 21.9 | 5.3 | 1 | 233 |
| IMGA\|Medtr3g082660.1 | Long-chain-fatty-acid--CoA ligase 4 | 36.0 (M:36.0) | 2.5 | 79.4 | 7.8 | 1 | 234 |
| IMGA\|AC233659_4.1 | Os03g0264300 protein (Fragment) | 35.9 (M:35.9) | 5.4 | 43.8 | 9.6 | 1 | 235 |
| IMGA\|Medtr4g124040.1 | ABC transporter B family member 11 | 35.9 (M:35.9) | 1.1 | 144.0 | 8.3 | 1 | 236 |
| IMGA\|Medtr3g019360.1 | U-box domain-containing protein 44 | 35.9 (M:35.9) | 2.2 | 91.6 | 6.2 | 1 | 237 |
| PP241_ARATH | Pentatricopeptide repeat-containing protein | 35.8 (M:35.8) | 1.1 | 162.2 | 7.0 | 1 | 238 |
| IMGA\|Medtr2g042840.1 | Unknown Protein | 35.8 (M:35.8) | 25.0 | 8.2 | 10.2 | 1 | 239 |
| IMGA\|Medtr8g044810.1 | Unknown Protein | 35.8 (M:35.8) | 11.1 | 13.5 | 10.7 | 1 | 240 |
| MSH2_MAIZE | DNA mismatch repair protein MSH2 | 35.8 (M:35.8) | 1.9 | 105.0 | 5.4 | 1 | 241 |
| ATPB_LONHI | ATP synthase subunit beta (EC 3.6.3.14) | 35.7 (M:35.7) | 0.0 | 22.7 | 4.7 | 1 | 242 |
| PUB39_ARATH | U-box domain-containing protein 39 | 35.7 (M:35.7) | 5.5 | 55.1 | 6.7 | 1 | 243 |
| CCSA_NUPAD | Cytochrome c biogenesis protein | 35.7 (M:35.7) | 8.1 | 35.1 | 9.5 | 1 | 244 |
| IMGA\|Medtr4g085540.1 | Poly(A)-binding protein | 35.6 (M:35.6) | 4.0 | 71.0 | 9.0 | 1 | 245 |
| NCPR_CATRO | NADPH--cytochrome P450 reductase | 35.6 (M:35.6) | 2.4 | 78.9 | 5.1 | 1 | 246 |
| RR4_PINKO | 30S ribosomal protein S4 | 35.6 (M:35.6) | 5.4 | 24.1 | 11.6 | 1 | 247 |
| CC135_CHLRE | Coiled-coil domain-containing protein lobo homolog (Fragment) | 35.5 (M:35.5) | 2.1 | 132.5 | 5.4 | 1 | 248 |
| IMGA\|Medtr4g112560.1 | Movement protein | 35.5 (M:35.5) | 7.4 | 18.8 | 5.4 | 1 | 249 |

| | | | | | | | |
|---|---|---|---|---|---|---|---|
| IMGA\|Medtr4g078290.1 | Os03g0349200 protein (Fragment) | 35.5 (M:35.5) | 2.0 | 76.5 | 10.1 | 1 | 250 |
| IMGA\|Medtr5g034170.1 | Unknown Protein | 35.4 (M:35.4) | 20.9 | 9.8 | 6.5 | 1 | 251 |
| PP443_ARATH | Pentatricopeptide repeat-containing protein | 35.4 (M:35.4) | 0.8 | 110.2 | 8.8 | 1 | 252 |
| IMGA\|Medtr5g006340.3 | Cysteine synthase | 35.3 (M:35.3) | 10.4 | 19.2 | 5.4 | 1 | 253 |
| IMGA\|Medtr2g033590.2 | Transmembrane protein 93 | 35.3 (M:35.3) | 28.2 | 12.9 | 9.7 | 1 | 254 |
| PEN5_ARATH | Marneral synthase | 35.2 (M:35.2) | 3.2 | 87.2 | 6.3 | 1 | 255 |
| IMGA\|Medtr2g008960.1 | Unknown Protein | 35.1 (M:35.1) | 4.5 | 42.4 | 9.1 | 1 | 256 |
| IMGA\|AC225507_16.1 | Eukaryotic translation initiation factor 3 subunit L | 35.1 (M:35.1) | 4.3 | 60.2 | 5.8 | 1 | 257 |
| IMGA\|Medtr3g056300.1 | NBS-containing resistance-like protein (Fragment) | 35.1 (M:35.1) | 2.6 | 76.0 | 6.4 | 1 | 258 |
| IMGA\|Medtr4g095360.1 | Subtilisin-like protease | 35.0 (M:35.0) | 2.0 | 79.8 | 6.4 | 1 | 259 |
| IF2C_PHAVU | Translation initiation factor IF-2 | 35.0 (M:35.0) | 1.5 | 108.7 | 9.9 | 1 | 260 |

**M. truncatula 18 d**

| Accession | Protein | Scores | S.Coverage | MW [kDa] | pI | Peptides | Rank |
|---|---|---|---|---|---|---|---|
| IMGA\|Medtr7g079820.1 | Convicilin (Fragment) | 2684.8 (M:2684.8) | 60.9 | 53.7 | 5.6 | 41 | 1 |
| IMGA\|Medtr7g079730.1 | Convicilin (Fragment) | 2678.8 (M:2678.8) | 44.0 | 53.1 | 5.3 | 15 | 2 |
| IMGA\|Medtr1g072600.1 | Legumin B (Fragment) | 2503.6 (M:2503.6) | 44.9 | 67.2 | 6.5 | 34 | 3 |
| IMGA\|Medtr7g079740.1 | Convicilin (Fragment) | 2473.2 (M:2473.2) | 61.5 | 54.2 | 5.6 | 8 | 4 |
| IMGA\|Medtr7g079780.1 | Convicilin (Fragment) | 2437.9 (M:2437.9) | 52.4 | 53.2 | 5.5 | 3 | 5 |
| IMGA\|Medtr7g079770.1 | Provicilin (Fragment) | 2241.8 (M:2241.8) | 51.0 | 53.1 | 5.3 | 8 | 6 |
| IMGA\|Medtr1g072630.1 | Legumin B | 1964.1 (M:1964.1) | 40.4 | 65.3 | 6.1 | 7 | 7 |
| IMGA\|Medtr1g072630.2 | Legumin B | 1871.7 (M:1871.7) | 47.1 | 63.5 | 6.4 | 1 | 8 |
| IMGA\|Medtr1g072610.2 | Legumin B (Fragment) | 1840.0 (M:1840.0) | 46.7 | 63.4 | 6.4 | 2 | 9 |
| IMGA\|Medtr2g099560.1 | Lipoxygenase | 685.9 (M:685.9) | 17.3 | 96.9 | 5.8 | 11 | 10 |
| IMGA\|Medtr2g099570.1 | Seed lipoxygenase-3 | 574.8 (M:574.8) | 15.0 | 97.3 | 6.1 | 9 | 11 |
| IMGA\|Medtr5g019780.1 | Vicilin-like antimicrobial peptides 2-3 (Fragment) | 534.4 (M:534.4) | 19.2 | 86.4 | 5.2 | 8 | 12 |
| VCLC_PEA | Vicilin precursor | 520.7 (M:520.7) | 12.6 | 52.2 | 5.3 | 2 | 13 |
| IMGA\|Medtr4g103920.1 | Glyceraldehyde-3-phosphate dehydrogenase (Fragment) | 519.0 (M:519.0) | 30.4 | 36.6 | 6.7 | 8 | 14 |
| IMGA\|Medtr2g066130.1 | Phosphoglycerate kinase | 475.0 (M:475.0) | 25.4 | 42.6 | 5.5 | 7 | 15 |
| IMGA\|Medtr2g083160.1 | Conglutin | 463.7 (M:463.7) | 24.3 | 16.8 | 6.3 | 6 | 16 |
| IMGA\|Medtr4g060780.1 | Allergen Gly m Bd (Fragment) | 460.5 (M:460.5) | 21.4 | 52.8 | 5.7 | 7 | 17 |
| PDI_MEDSA | Protein disulfide-isomerase precursor (EC 5.3.4.1) | 323.7 (M:323.7) | 14.8 | 57.1 | 4.8 | 6 | 18 |
| RBL_PASQU | Ribulose bisphosphate carboxylase large chain (Fragment) | 315.9 (M:315.9) | 12.9 | 51.4 | 6.1 | 5 | 19 |
| IMGA\|Medtr1g083960.1 | Calreticulin | 308.2 (M:308.2) | 17.5 | 48.4 | 4.3 | 5 | 20 |
| IMGA\|Medtr1g108770.1 | ATP synthase subunit beta | 306.5 (M:306.5) | 9.1 | 120.9 | 5.8 | 6 | 21 |
| IMGA\|Medtr5g069050.1 | Fructose-bisphosphate aldolase | 302.6 (M:302.6) | 9.6 | 78.3 | 5.7 | 5 | 22 |
| IMGA\|Medtr7g024390.1 | Heat shock protein 70 | 284.7 (M:284.7) | 10.2 | 70.9 | 4.9 | 4 | 23 |
| IMGA\|Medtr3g109190.1 | Oleosin | 282.4 (M:282.4) | 17.4 | 16.8 | 9.3 | 3 | 24 |
| IMGA\|Medtr3g085850.1 | Glyceraldehyde 3-phosphate dehydrogenase | 273.1 (M:273.1) | 20.3 | 37.0 | 7.7 | 1 | 25 |
| PGKH_TOBAC | Phosphoglycerate kinase | 265.1 (M:265.1) | 11.9 | 50.1 | 9.2 | 1 | 26 |
| IMGA\|AC233663_14.1 | RuBisCO large subunit-binding protein subunit alpha | 255.3 (M:255.3) | 18.8 | 49.3 | 4.8 | 5 | 27 |
| EF1A_MAIZE | Elongation factor 1-alpha (EF-1-alpha) | 246.7 (M:246.7) | 17.2 | 49.2 | 9.8 | 5 | 28 |
| IMGA\|Medtr5g098780.3 | Chlorophyll a-b binding protein 8 | 239.7 (M:239.7) | 17.3 | 29.7 | 8.8 | 4 | 29 |
| IMGA\|Medtr7g117920.1 | ATP synthase subunit beta, chloroplastic | 226.8 (M:226.8) | 15.8 | 33.8 | 9.5 | 3 | 30 |
| IMGA\|AC157372_6.1 | Oxygen-evolving enhancer protein 1 (Fragment) | 226.6 (M:226.6) | 26.4 | 35.0 | 6.3 | 4 | 31 |
| IMGA\|Medtr2g025120.1 | 1-aminocyclopropane-1-carboxylate oxidase (Fragment) | 225.5 (M:225.5) | 10.4 | 36.1 | 4.9 | 3 | 32 |
| IMGA\|Medtr3g114420.1 | Subtilisin-type protease | 223.0 (M:223.0) | 8.9 | 82.4 | 8.4 | 3 | 33 |
| CALM_MALDO | Calmodulin | 204.8 (M:204.8) | 42.3 | 16.8 | 4.0 | 4 | 34 |
| ATPAM_PEA | ATP synthase subunit alpha, mitochondrial | 203.8 (M:203.8) | 9.1 | 55.0 | 6.0 | 3 | 35 |
| ATPA_PEA | ATP synthase subunit alpha, chloroplastic | 199.3 (M:199.3) | 12.6 | 54.6 | 5.6 | 4 | 36 |
| IMGA\|Medtr4g124660.2 | Sucrose synthase | 199.1 (M:199.1) | 6.7 | 92.2 | 5.8 | 4 | 37 |
| IMGA\|Medtr8g106790.1 | Guanine nucleotide-binding protein subunit beta-like protein | 198.6 (M:198.6) | 20.9 | 35.7 | 7.8 | 3 | 38 |
| IMGA\|AC146630_2.1 | 2-cys peroxiredoxin BAS1 | 198.6 (M:198.6) | 21.1 | 29.0 | 6.1 | 3 | 39 |
| IMGA\|Medtr2g029730.1 | Peroxidase | 195.0 (M:195.0) | 4.3 | 37.4 | 6.2 | 2 | 40 |
| IMGA\|Medtr1g087520.1 | Protein disulfide-isomerase | 193.8 (M:193.8) | 19.1 | 41.3 | 5.4 | 4 | 41 |
| IMGA\|Medtr5g077000.2 | UTP-glucose 1 phosphate uridylyltransferase | 193.8 (M:193.8) | 8.5 | 51.4 | 5.1 | 3 | 42 |
| HSP7S_PEA | Stromal 70 kDa heat shock-related protein | 192.5 (M:192.5) | 3.5 | 75.5 | 5.1 | 3 | 43 |
| IMGA\|Medtr3g098420.1 | Staphylococcal nuclease domain-containing protein 1 | 191.8 (M:191.8) | 7.0 | 108.2 | 6.6 | 4 | 44 |
| ENO_RICCO | Enolase (EC 4.2.1.11) | 191.1 (M:191.1) | 7.4 | 47.9 | 5.5 | 2 | 45 |
| IMGA\|Medtr1g043040.1 | Malate dehydrogenase | 185.8 (M:185.8) | 13.3 | 35.5 | 6.1 | 3 | 46 |
| IMGA\|Medtr3g088970.1 | Argininosuccinate synthase | 180.0 (M:180.0) | 13.0 | 52.7 | 6.3 | 4 | 47 |
| IMGA\|Medtr7g118060.1 | 60s acidic ribosomal protein P1 | 177.2 (M:177.2) | 46.9 | 11.4 | 4.0 | 3 | 48 |
| IMGA\|Medtr7g052690.1 | Early tobacco anther 1 | 174.4 (M:174.4) | 24.2 | 16.9 | 4.5 | 2 | 49 |
| ENO_SOLLC | Enolase | 166.4 (M:166.4) | 9.0 | 47.8 | 5.6 | 1 | 50 |
| IMGA\|Medtr2g020360.1 | Acyl carrier protein 1 | 162.9 (M:162.9) | 4.3 | 37.9 | 5.3 | 2 | 51 |
| RUBB_SECCE | RuBisCO large subunit-binding protein subunit beta | 162.6 (M:162.6) | 7.6 | 53.4 | 4.7 | 3 | 52 |
| IMGA\|Medtr1g071710.1 | Vicilin-like antimicrobial peptides 2-2 | 161.9 (M:161.9) | 5.1 | 56.0 | 6.0 | 2 | 53 |
| IMGA\|Medtr2g066110.1 | Phosphoglycerate kinase (Fragment) | 158.8 (M:158.8) | 11.7 | 50.0 | 6.8 | 1 | 54 |
| PSBP_PEA | Oxygen-evolving enhancer protein 2 | 158.1 (M:158.1) | 13.5 | 28.0 | 9.1 | 3 | 55 |
| IMGA\|Medtr4g132270.2 | Lactoylglutathione lyase | 155.7 (M:155.7) | 12.8 | 27.0 | 4.8 | 2 | 56 |
| IMGA\|Medtr3g077050.1 | 60S ribosomal protein L27a-3 | 152.2 (M:152.2) | 19.7 | 16.6 | 11.2 | 3 | 57 |
| HSP80_SOLLC | Heat shock cognate protein 80 | 146.6 (M:146.6) | 7.0 | 80.1 | 4.8 | 3 | 58 |
| H2B_PEA | Histone H2B (Fragments) | 146.1 (M:146.1) | 12.5 | 13.5 | 10.7 | 2 | 59 |
| BIP2_MAIZE | Luminal-binding protein 2 precursor (BiP2) | 143.5 (M:143.5) | 4.5 | 73.0 | 5.0 | 1 | 60 |
| IMGA\|Medtr7g009330.1 | Outer plastidial membrane protein porin | 142.5 (M:142.5) | 11.9 | 29.6 | 9.4 | 2 | 61 |
| IMGA\|Medtr8g070780.1 | Defensin | 138.2 (M:138.2) | 31.2 | 8.3 | 10.8 | 2 | 62 |
| CALM1_SOLTU | Calmodulin-1 | 137.2 (M:137.2) | 30.9 | 16.9 | 4.0 | 1 | 63 |
| IMGA\|AC235488_13.1 | 40S ribosomal protein S5 | 136.9 (M:136.9) | 11.3 | 27.9 | 9.8 | 2 | 64 |
| ACT2_SOLTU | Actin-46 | 135.8 (M:135.8) | 11.0 | 37.1 | 5.2 | 2 | 65 |
| LEG_CICAR | Legumin | 135.7 (M:135.7) | 2.4 | 56.2 | 6.2 | 2 | 66 |
| IMGA\|Medtr2g038250.1 | 60S ribosomal protein L7-4 | 129.5 (M:129.5) | 11.5 | 28.5 | 10.4 | 1 | 67 |
| IMGA\|Medtr5g033090.1 | 60S ribosomal protein | 128.8 (M:128.8) | 14.0 | 18.5 | 11.0 | 1 | 68 |
| IMGA\|Medtr2g008050.1 | Actin | 127.8 (M:127.8) | 9.8 | 41.6 | 5.2 | 1 | 69 |
| IMGA\|Medtr4g057240.1 | Superoxide dismutase | 126.4 (M:126.4) | 11.9 | 20.7 | 6.0 | 2 | 70 |
| IMGA\|Medtr5g025120.1 | 60S ribosomal protein L4-1 | 122.4 (M:122.4) | 7.8 | 44.9 | 10.9 | 2 | 71 |
| IMGA\|Medtr6g018300.1 | Ribulose bisphosphate carboxylase small chain (Fragment) | 120.5 (M:120.5) | 18.1 | 19.8 | 9.4 | 2 | 72 |
| IMGA\|Medtr7g086300.4 | Methionine synthase | 117.1 (M:117.1) | 15.0 | 25.4 | 6.2 | 2 | 73 |
| IMGA\|Medtr3g106580.1 | Protease inhibitor | 116.8 (M:116.8) | 3.9 | 103.5 | 9.6 | 2 | 74 |
| IMGA\|Medtr8g104540.2 | Phosphoglucomutase | 116.6 (M:116.6) | 7.2 | 63.2 | 5.4 | 2 | 75 |
| IMGA\|Medtr8g012380.1 | FK506-binding protein 2 | 115.6 (M:115.6) | 9.9 | 16.5 | 9.0 | 1 | 76 |
| IMGA\|Medtr8g067080.1 | ADP-ribosylation factor GTPase-activating protein | 115.3 (M:115.3) | 13.9 | 43.9 | 9.7 | 2 | 77 |
| IMGA\|Medtr4g079780.1 | Ketol-acid reductoisomerase | 114.6 (M:114.6) | 5.5 | 62.9 | 6.3 | 2 | 78 |
| IMGA\|Medtr4g075290.1 | Peptidyl-prolyl cis-trans isomerase | 113.7 (M:113.7) | 24.4 | 18.2 | 9.5 | 2 | 79 |
| IMGA\|Medtr2g028190.1 | Aspartic proteinase nepenthesin-2 | 112.3 (M:112.3) | 8.6 | 48.6 | 9.7 | 2 | 80 |
| IMGA\|Medtr5g018940.1 | 40S ribosomal protein S4 | 111.8 (M:111.8) | 12.1 | 29.9 | 10.8 | 2 | 81 |
| ENO2_HEVBR | Enolase 2 (EC 4.2.1.11) | 109.4 (M:109.4) | 3.8 | 47.9 | 5.9 | 1 | 82 |
| IMGA\|Medtr3g092600.1 | 60S acidic ribosomal protein P1 | 107.1 (M:107.1) | 50.0 | 11.1 | 4.1 | 2 | 83 |
| IMGA\|Medtr3g118030.1 | 60S ribosomal protein L5 | 106.9 (M:106.9) | 8.5 | 37.5 | 9.6 | 2 | 84 |
| IMGA\|Medtr7g113650.1 | Glucose and ribitol dehydrogenase homolog 1 | 106.4 (M:106.4) | 15.7 | 31.8 | 7.7 | 2 | 85 |
| IMGA\|Medtr1g025430.1 | Endoplasmin homolog | 105.1 (M:105.1) | 3.5 | 94.1 | 4.6 | 2 | 86 |
| IMGA\|Medtr7g088680.1 | Nascent polypeptide-associated complex subunit | 103.3 (M:103.3) | 23.6 | 22.1 | 4.2 | 2 | 87 |
| LEGA_PEA | Legumin A | 101.8 (M:101.8) | 3.9 | 58.8 | 6.2 | 1 | 88 |

| | | | | | | | |
|---|---|---|---|---|---|---|---|
| IMGA\|Medtr2g095730.1 | Serine carboxypeptidase | 101.4 (M:101.4) | 6.5 | 57.3 | 5.6 | 2 | 89 |
| IMGA\|Medtr5g064580.1 | 14-3-3 protein (Fragment) | 99.9 (M:99.9) | 5.8 | 29.2 | 4.5 | 1 | 90 |
| IMGA\|Medtr4g070080.1 | Glycine-rich RNA binding protein 1 | 98.1 (M:98.1) | 14.3 | 15.7 | 5.4 | 2 | 91 |
| IMGA\|Medtr3g087590.2 | L-myo inositol-1 phosphate synthase 1 | 97.7 (M:97.7) | 4.7 | 51.9 | 5.2 | 2 | 92 |
| IMGA\|Medtr2g095750.1 | Serine carboxypeptidase | 97.2 (M:97.2) | 6.5 | 57.3 | 5.2 | 1 | 93 |
| IMGA\|Medtr2g014030.1 | 40S ribosomal protein S6 | 96.5 (M:96.5) | 6.0 | 28.2 | 11.5 | 1 | 94 |
| IMGA\|Medtr8g105340.1 | 40S ribosomal protein S2 | 95.9 (M:95.9) | 11.1 | 30.4 | 11.0 | 2 | 95 |
| IMGA\|Medtr2g069050.1 | Elongation factor EF-2 (Fragment) | 91.9 (M:91.9) | 3.6 | 94.1 | 5.7 | 2 | 96 |
| IMGA\|Medtr4g112010.1 | Potassium channel | 90.6 (M:90.6) | 2.9 | 63.4 | 10.3 | 1 | 97 |
| IMGA\|Medtr3g092090.1 | YSL transporter 3 | 90.4 (M:90.4) | 4.6 | 92.9 | 9.0 | 2 | 98 |
| PSBE_OENBE | Cytochrome b559 subunit alpha | 88.8 (M:88.8) | 14.5 | 9.3 | 4.9 | 1 | 99 |
| H2A3_ORYSI | Probable histone H2A.3 | 88.4 (M:88.4) | 20.1 | 13.9 | 10.7 | 1 | 100 |
| IMGA\|Medtr3g098430.1 | Calnexin homolog | 86.1 (M:86.1) | 6.1 | 61.7 | 4.6 | 2 | 101 |
| PSBD_NEPOL | Photosystem II D2 protein | 85.3 (M:85.3) | 6.2 | 39.2 | 5.4 | 2 | 102 |
| IMGA\|Medtr5g088660.1 | Elongation factor 1-beta 1 | 83.6 (M:83.6) | 17.0 | 24.2 | 4.5 | 2 | 103 |
| GLYG1_SOYBN | Glycinin G1 | 82.1 (M:82.1) | 4.2 | 55.7 | 5.8 | 2 | 104 |
| PSBQ_ONOVI | Oxygen-evolving enhancer protein 3 | 82.0 (M:82.0) | 5.6 | 24.8 | 10.0 | 1 | 105 |
| IMGA\|Medtr4g016670.1 | Ribosomal protein S8 | 81.6 (M:81.6) | 5.4 | 31.4 | 10.4 | 2 | 106 |
| IMGA\|Medtr3g104720.1 | Puromycin-sensitive aminopeptidase | 81.0 (M:81.0) | 3.3 | 98.9 | 5.4 | 2 | 107 |
| IMGA\|Medtr3g085740.1 | Beta-oxoacyl-ACP reductase (Fragment) | 80.9 (M:80.9) | 7.2 | 33.5 | 9.9 | 1 | 108 |
| IMGA\|Medtr6g087990.1 | Peroxiredoxin | 80.7 (M:80.7) | 12.3 | 17.5 | 5.5 | 1 | 109 |
| GLTB2_ARATH | Ferredoxin-dependent glutamate synthase 2 | 79.3 (M:79.3) | 1.8 | 177.6 | 6.6 | 1 | 110 |
| IMGA\|Medtr5g006130.1 | Photosystem I reaction center subunit II | 78.4 (M:78.4) | 15.8 | 23.0 | 10.1 | 1 | 111 |
| IMGA\|Medtr4g024550.1 | 40S ribosomal protein S13 | 77.0 (M:77.0) | 9.4 | 15.8 | 10.8 | 2 | 112 |
| IMGA\|Medtr4g035360.1 | GDSL esterase/lipase | 76.8 (M:76.8) | 2.0 | 112.9 | 9.4 | 1 | 113 |
| IMGA\|Medtr4g063060.1 | 60S ribosomal protein L23a | 75.8 (M:75.8) | 8.6 | 17.1 | 10.7 | 1 | 114 |
| IMGA\|Medtr5g061080.1 | Unknown Protein | 75.5 (M:75.5) | 7.7 | 23.0 | 9.4 | 2 | 115 |
| IMGA\|Medtr3g105860.1 | Unknown Protein | 74.1 (M:74.1) | 15.0 | 10.8 | 4.9 | 1 | 116 |
| IMGA\|AC233685_77.1 | Ribulose bisphosphate carboxylase small chain 3A | 73.5 (M:73.5) | 7.7 | 20.2 | 9.5 | 1 | 117 |
| IMGA\|Medtr5g091120.2 | 60S ribosomal protein L18a | 72.4 (M:72.4) | 9.5 | 17.5 | 11.0 | 1 | 118 |
| IMGA\|AC235488_9.1 | 60S ribosomal protein L21 | 71.6 (M:71.6) | 6.1 | 18.7 | 11.0 | 1 | 119 |
| IMGA\|Medtr8g085580.1 | Unknown Protein | 70.9 (M:70.9) | 12.8 | 19.3 | 8.9 | 1 | 120 |
| REHY_MEDTR | 1-Cys peroxiredoxin | 70.4 (M:70.4) | 11.5 | 24.4 | 6.1 | 1 | 121 |
| IMGA\|Medtr1g088450.1 | 60S ribosomal protein L22-like | 70.3 (M:70.3) | 10.9 | 13.6 | 10.0 | 1 | 122 |
| ENO_ORYSJ | Enolase OS | 69.3 (M:69.3) | 5.6 | 47.9 | 5.3 | 1 | 123 |
| IMGA\|Medtr5g026750.1 | 60S ribosomal protein L13 | 68.8 (M:68.8) | 13.8 | 27.4 | 11.4 | 1 | 124 |
| IMGA\|Medtr4g049550.1 | Disease resistance response protein 206 | 68.4 (M:68.4) | 6.4 | 25.8 | 9.7 | 1 | 125 |
| IMGA\|Medtr1g081410.1 | 40S ribosomal protein S24 | 66.0 (M:66.0) | 10.9 | 15.7 | 11.1 | 1 | 126 |
| IMGA\|Medtr7g111590.3 | 60S ribosomal protein L13 | 65.8 (M:65.8) | 17.3 | 15.7 | 10.8 | 1 | 127 |
| RL24_HORVU | 60S ribosomal protein L24 | 64.8 (M:64.8) | 7.4 | 18.4 | 11.4 | 1 | 128 |
| IMGA\|Medtr8g076210.1 | Thymus-specific serine protease | 64.0 (M:64.0) | 2.9 | 51.0 | 5.0 | 1 | 129 |
| IMGA\|Medtr7g113790.1 | Basic 7S globulin 2 | 63.8 (M:63.8) | 5.3 | 48.1 | 10.0 | 1 | 130 |
| 6PGD2_ARATH | 6-phosphogluconate dehydrogenase | 63.4 (M:63.4) | 4.9 | 53.3 | 5.5 | 1 | 131 |
| IMGA\|Medtr8g046140.2 | 60S ribosomal protein L11 | 62.6 (M:62.6) | 12.0 | 13.2 | 10.4 | 1 | 132 |
| EFTU_PEA | Elongation factor Tu | 62.4 (M:62.4) | 2.3 | 53.0 | 6.7 | 1 | 133 |
| IMGA\|Medtr4g121880.1 | Dihydrolipoyl dehydrogenase | 60.6 (M:60.6) | 3.2 | 59.8 | 6.8 | 1 | 134 |
| SODM_PRUPE | Superoxide dismutase [Mn] | 60.5 (M:60.5) | 6.6 | 25.4 | 9.1 | 1 | 135 |
| RS271_ARATH | 40S ribosomal protein S27-1 | 59.9 (M:59.9) | 20.2 | 9.4 | 10.6 | 1 | 136 |
| IMGA\|Medtr4g061140.1 | Cytosolic ascorbate peroxidase | 59.3 (M:59.3) | 7.2 | 27.1 | 5.5 | 1 | 137 |
| EF1B_ORYSJ | Elongation factor 1-beta | 59.2 (M:59.2) | 7.6 | 23.8 | 4.5 | 1 | 138 |
| IMGA\|Medtr3g115930.1 | Unknown Protein | 58.6 (M:58.6) | 9.5 | 17.9 | 10.2 | 1 | 139 |
| IMGA\|Medtr4g053120.1 | Ferredoxin-6 | 58.5 (M:58.5) | 14.1 | 15.1 | 4.5 | 1 | 140 |
| IMGA\|Medtr6g021670.1 | 40S ribosomal protein S7-like protein | 57.8 (M:57.8) | 13.6 | 21.9 | 10.3 | 1 | 141 |
| IMGA\|Medtr6g005040.1 | Oleosin 1 | 57.7 (M:57.7) | 7.3 | 20.7 | 9.3 | 1 | 142 |
| IMGA\|Medtr6g052140.1 | 40S ribosomal protein S3 | 56.8 (M:56.8) | 6.0 | 26.2 | 10.4 | 1 | 143 |
| ACLB1_ARATH | ATP-citrate synthase beta chain protein 1 | 55.7 (M:55.7) | 3.0 | 65.8 | 8.8 | 1 | 144 |
| IMGA\|Medtr7g092720.1 | 40S ribosomal protein S20-2 | 55.6 (M:55.6) | 9.8 | 13.7 | 10.2 | 1 | 145 |
| PDI12_ORYSJ | Protein disulfide isomerase-like 1-2 | 54.3 (M:54.3) | 1.9 | 57.3 | 4.5 | 1 | 146 |
| IMGA\|Medtr8g058480.1 | Zinc finger CCCH domain-containing protein 51 | 54.1 (M:54.1) | 7.0 | 39.0 | 4.7 | 1 | 147 |
| IMGA\|Medtr2g064660.2 | Photosystem II 10 kDa polypeptide | 52.7 (M:52.7) | 11.8 | 13.6 | 10.2 | 1 | 148 |
| PSBP1_ARATH | Oxygen-evolving enhancer protein 2-1 | 52.6 (M:52.6) | 5.3 | 28.1 | 7.7 | 1 | 149 |
| RS10_ORYSJ | 40S ribosomal protein S10 | 51.3 (M:51.3) | 7.1 | 20.3 | 10.2 | 1 | 150 |
| IMGA\|Medtr2g008620.1 | (3R)-hydroxymyristoyl- | 51.2 (M:51.2) | 9.8 | 15.7 | 9.2 | 1 | 151 |
| G3PB_TOBAC | Glyceraldehyde-3-phosphate dehydrogenase B | 50.8 (M:50.8) | 3.9 | 47.4 | 9.8 | 1 | 152 |
| IMGA\|Medtr7g075270.1 | Fasciclin-like arabinogalactan protein 13 | 50.8 (M:50.8) | 4.1 | 42.5 | 5.8 | 1 | 153 |
| IMGA\|Medtr2g005570.2 | Elongation factor 1-gamma | 50.0 (M:50.0) | 4.5 | 47.7 | 7.7 | 1 | 154 |
| IMGA\|Medtr7g084030.1 | Lysosomal alpha-mannosidase | 49.2 (M:49.2) | 2.7 | 118.0 | 5.6 | 1 | 155 |
| IMGA\|Medtr7g012950.1 | UDP-glucose dehydrogenase | 49.1 (M:49.1) | 3.5 | 52.9 | 5.6 | 1 | 156 |
| IMGA\|Medtr5g057990.1 | Alpha-D-xylosidase | 48.9 (M:48.9) | 1.8 | 102.9 | 6.3 | 1 | 157 |
| IMGA\|Medtr4g095450.2 | Peroxidase | 48.2 (M:48.2) | 8.4 | 21.9 | 9.7 | 1 | 158 |
| IMGA\|Medtr6g069630.1 | Aldose 1-epimerase family protein expressed | 46.8 (M:46.8) | 7.1 | 36.9 | 5.9 | 1 | 159 |
| IMGA\|Medtr4g075340.1 | Translocon-associated protein subunit alpha | 46.5 (M:46.5) | 5.9 | 27.5 | 4.6 | 1 | 160 |
| IMGA\|Medtr7g026030.1 | Heat shock protein 70 (Fragment) | 46.0 (M:46.0) | 1.9 | 69.6 | 5.3 | 1 | 161 |
| IMGA\|Medtr7g098980.1 | Unknown Protein | 45.6 (M:45.6) | 4.9 | 39.7 | 10.1 | 1 | 162 |
| IMGA\|Medtr3g114850.1 | Plastocyanin | 45.6 (M:45.6) | 14.4 | 17.1 | 4.8 | 1 | 163 |
| IMGA\|Medtr3g018780.1 | Annexin-like protein RJ4 | 45.4 (M:45.4) | 4.1 | 36.2 | 8.7 | 1 | 164 |
| MDHM_IMPCY | Malate dehydrogenase | 45.1 (M:45.1) | 61.3 | 3.1 | 7.0 | 1 | 165 |
| IMGA\|Medtr2g013640.1 | Unknown Protein | 45.0 (M:45.0) | 1.0 | 106.3 | 9.8 | 1 | 166 |
| IMGA\|Medtr5g084930.1 | Protein disulfide isomerase family | 44.8 (M:44.8) | 3.2 | 47.4 | 5.3 | 1 | 167 |
| IMGA\|Medtr8g102620.1 | Malic enzyme (Fragment) | 44.7 (M:44.7) | 2.5 | 65.3 | 6.0 | 1 | 168 |
| IMGA\|Medtr7g069980.1 | Ferritin-1, chloroplastic | 44.7 (M:44.7) | 5.9 | 28.8 | 6.0 | 1 | 169 |
| IMGA\|AC235753_1.1 | Cysteine proteinase | 44.5 (M:44.5) | 6.6 | 40.3 | 6.1 | 1 | 170 |
| IMGA\|Medtr3g102040.1 | Poly(A)-binding protein | 44.5 (M:44.5) | 3.7 | 68.4 | 6.2 | 1 | 171 |
| AB15C_ARATH | Putative ABC transporter C family | 44.5 (M:44.5) | 2.7 | 117.2 | 6.2 | 1 | 172 |
| AGAL_CAPAA | Alpha-galactosidase (Fragment) | 43.8 (M:43.8) | 100.0 | 1.4 | 6.7 | 1 | 173 |
| IMGA\|Medtr8g092040.1 | Unknown Protein | 43.8 (M:43.8) | 4.5 | 27.7 | 8.8 | 1 | 174 |
| IMGA\|Medtr4g062130.1 | Nudix hydrolase 9 | 43.7 (M:43.7) | 5.0 | 33.6 | 5.3 | 1 | 175 |
| TGT1_ARATH | Trihelix transcription factor GT-1 | 43.7 (M:43.7) | 2.5 | 46.6 | 6.4 | 1 | 176 |
| SECA_SPIOL | Preprotein translocase subunit secA | 43.6 (M:43.6) | 1.5 | 116.5 | 5.8 | 1 | 177 |
| IMGA\|Medtr8g106020.1 | 40S ribosomal protein S16 | 43.2 (M:43.2) | 7.1 | 16.1 | 11.0 | 1 | 178 |
| RS281_ARATH | 40S ribosomal protein S28 | 43.0 (M:43.0) | 18.8 | 7.4 | 11.3 | 1 | 179 |

| | | | | | | | |
|---|---|---|---|---|---|---|---|
| IMGA\|Medtr1g023210.1 | Cysteine proteinase | 43.0 (M:43.0) | 5.8 | 39.9 | 6.6 | 1 | 180 |
| NDK1_PSEMZ | Nucleoside diphosphate kinase 1 (Fragments) | 42.3 (M:42.3) | 50.0 | 1.9 | 4.4 | 1 | 181 |
| IMGA\|Medtr8g052300.1 | S locus glycoprotein (Fragment) | 42.2 (M:42.2) | 7.4 | 37.0 | 9.1 | 1 | 182 |
| IMGA\|Medtr1g075790.1 | Os12g0236050 protein (Fragment) | 42.1 (M:42.1) | 1.9 | 111.0 | 5.0 | 1 | 183 |
| TBB1_DAUCA | Tubulin beta-1 chain (Beta-1 tubulin) (Fragmennt) | 42.1 (M:42.1) | 3.8 | 35.8 | 4.6 | 1 | 184 |
| CB22_POPEU | Chlorophyll a-b binding protein 2 | 41.7 (M:41.7) | 25.0 | 3.8 | 9.5 | 1 | 185 |
| IMGA\|Medtr1g106900.2 | 40S ribosomal protein S18 | 41.7 (M:41.7) | 9.4 | 10.2 | 11.9 | 1 | 186 |
| IMGA\|Medtr8g074010.1 | Phosphoserine aminotransferase | 41.1 (M:41.1) | 4.2 | 47.2 | 9.1 | 1 | 187 |
| TKTC_CRAPL | Transketolase | 40.8 (M:40.8) | 4.0 | 56.2 | 5.8 | 1 | 188 |
| IMGA\|Medtr7g072730.1 | Non-specific lipid-transfer protein | 40.1 (M:40.1) | 21.4 | 12.4 | 4.8 | 1 | 189 |
| IMGA\|Medtr3g093110.1 | Ribosomal protein L9 (Fragment) | 40.0 (M:40.0) | 6.2 | 21.7 | 9.9 | 1 | 190 |
| IMGA\|Medtr5g081710.3 | 60S ribosomal protein L35a | 39.9 (M:39.9) | 13.8 | 15.1 | 11.2 | 1 | 191 |
| IMGA\|Medtr2g031390.1 | Reticuline oxidase-like protein | 39.6 (M:39.6) | 4.4 | 61.4 | 9.0 | 1 | 192 |
| IMGA\|Medtr8g076770.1 | Protein transport protein Sec61 subunit alpha | 39.3 (M:39.3) | 1.9 | 52.2 | 9.7 | 1 | 193 |
| IMGA\|Medtr8g035730.1 | BEACH domain-containing protein lvsC | 39.2 (M:39.2) | 4.0 | 72.4 | 5.0 | 1 | 194 |
| IMGA\|Medtr1g014120.1 | Early nodulin-like protein 1 | 39.2 (M:39.2) | 5.4 | 19.4 | 8.9 | 1 | 195 |
| CB4A_ARATH | Chlorophyll a-b binding protein CP29.1 | 39.2 (M:39.2) | 5.2 | 31.1 | 5.7 | 1 | 196 |
| IMGA\|Medtr5g080450.1 | Ribulose-1 5-bisphosphate carboxylase/oxygenase activase 1 | 39.2 (M:39.2) | 3.4 | 52.3 | 6.1 | 1 | 197 |
| IMGA\|Medtr2g098010.1 | Proteasome subunit beta type | 39.0 (M:39.0) | 11.3 | 25.4 | 6.3 | 1 | 198 |
| TBA_PRUDU | Tubulin alpha chain | 39.0 (M:39.0) | 4.4 | 49.5 | 4.8 | 1 | 199 |
| IMGA\|Medtr1g023120.1 | Beta-galactosidase | 38.9 (M:38.9) | 3.6 | 83.1 | 9.2 | 1 | 200 |
| IMGA\|Medtr7g028570.1 | Unknown Protein | 38.8 (M:38.8) | 6.6 | 26.9 | 4.7 | 1 | 201 |
| P2A06_ARATH | Uncharacterized protein PHLOEM PROTEIN 2-LIKE A6 | 38.5 (M:38.5) | 6.1 | 44.1 | 9.3 | 1 | 202 |
| IMGA\|Medtr7g006560.1 | Transaldolase | 38.5 (M:38.5) | 4.1 | 48.5 | 6.2 | 1 | 203 |
| IMGA\|Medtr4g130970.1 | BTB/POZ domain-containing protein KCTD6 | 38.5 (M:38.5) | 4.1 | 49.4 | 5.1 | 1 | 204 |
| IMGA\|Medtr7g051650.3 | Beta-glucosidase 24 | 38.4 (M:38.4) | 5.3 | 46.7 | 8.6 | 1 | 205 |
| IMGA\|Medtr4g035550.1 | PHD finger protein At1g33420 | 38.3 (M:38.3) | 2.6 | 80.8 | 7.9 | 1 | 206 |
| IMGA\|Medtr3g051310.1 | Acyl-peptide hydrolase-like | 38.1 (M:38.1) | 2.7 | 80.8 | 5.5 | 1 | 207 |
| ALFL3_ARATH | PHD finger protein ALFIN-LIKE 3 | 38.1 (M:38.1) | 7.2 | 28.2 | 4.9 | 1 | 208 |
| LLOS5_ARTAN | R-linalool synthase QH5, chloroplastic | 37.9 (M:37.9) | 1.9 | 67.3 | 6.2 | 1 | 209 |
| IMGA\|Medtr4g006770.1 | Unknown Protein (AHRD V1) chr04_pseudomolecule | 37.8 (M:37.8) | 29.7 | 7.4 | 10.1 | 1 | 210 |
| IMGA\|Medtr8g013980.1 | Homeobox-leucine zipper protein | 37.6 (M:37.6) | 2.4 | 91.8 | 6.2 | 1 | 211 |
| PSBB_CUCSA | Photosystem II CP47 chlorophyll apoprotein | 37.6 (M:37.6) | 2.8 | 55.9 | 6.3 | 1 | 212 |
| IMGA\|Medtr8g106860.1 | Unknown Protein (AHRD V1) chr08_pseudomolecule | 37.5 (M:37.5) | 7.4 | 13.0 | 7.8 | 1 | 213 |
| IMGA\|Medtr8g076910.1 | Tir-nbs-lrr resistance protein (Fragment) | 37.5 (M:37.5) | 1.1 | 126.6 | 6.2 | 1 | 214 |
| P24B2_ARATH | Transmembrane emp24 domain-containing protein p24beta2 | 37.4 (M:37.4) | 5.3 | 24.3 | 5.9 | 1 | 215 |
| IMGA\|AC229724_1067.1 | Genomic DNA chromosome 3 | 37.2 (M:37.2) | 3.3 | 71.1 | 7.8 | 1 | 216 |
| ISW2_ARATH | Putative chromatin-remodeling complex ATPase | 37.1 (M:37.1) | 1.5 | 122.4 | 5.6 | 1 | 217 |
| IMGA\|Medtr5g006340.3 | Cysteine synthase | 37.1 (M:37.1) | 9.3 | 19.2 | 5.4 | 1 | 218 |
| CYF_LOBMA | Apocytochrome | 37.1 (M:37.1) | 5.9 | 35.4 | 9.1 | 1 | 219 |
| IMGA\|Medtr5g021460.1 | Unknown Protein | 37.0 (M:37.0) | 21.6 | 10.9 | 11.0 | 1 | 220 |
| AB30G_ARATH | ABC transporter G family member 30 | 37.0 (M:37.0) | 2.0 | 157.7 | 9.3 | 1 | 221 |
| IMGA\|Medtr3g025940.1 | RING finger protein 44 | 37.0 (M:37.0) | 4.8 | 49.4 | 4.7 | 1 | 222 |
| RL37A_GOSHI | 60S ribosomal protein L37a | 37.0 (M:37.0) | 17.4 | 10.2 | 11.3 | 1 | 223 |
| IMGA\|Medtr2g094150.1 | Unknown Protein | 36.9 (M:36.9) | 11.0 | 19.7 | 10.0 | 1 | 224 |
| IMGA\|Medtr7g100660.1 | E3 ubiquitin-protein ligase UPL4 | 36.8 (M:36.8) | 6.0 | 31.4 | 5.1 | 1 | 225 |
| IMGA\|Medtr3g027280.1 | Vegetative lectin | 36.8 (M:36.8) | 5.0 | 30.2 | 7.7 | 1 | 226 |
| IMGA\|Medtr3g087830.1 | Apoptotic chromatin condensation inducer in the nucleus | 36.8 (M:36.8) | 2.2 | 79.2 | 4.6 | 1 | 227 |
| ORP1A_ARATH | Oxysterol-binding protein-related protein 1A | 36.6 (M:36.6) | 2.5 | 87.3 | 6.3 | 1 | 228 |
| RNH2A_ARATH | Ribonuclease H2 subunit A | 36.5 (M:36.5) | 5.4 | 33.1 | 6.3 | 1 | 229 |
| IMGA\|Medtr8g102330.2 | Profilin | 36.4 (M:36.4) | 18.8 | 11.1 | 5.0 | 1 | 230 |
| IMGA\|Medtr2g039680.1 | Nucleosome assembly protein 1-like | 36.4 (M:36.4) | 4.9 | 41.8 | 4.1 | 1 | 231 |
| IMGA\|Medtr2g077700.1 | Unknown Protein | 36.4 (M:36.4) | 2.8 | 89.5 | 7.8 | 1 | 232 |
| IMGA\|Medtr5g014260.2 | Glycine cleavage system H protein | 36.3 (M:36.3) | 10.3 | 16.7 | 4.5 | 1 | 233 |
| IMGA\|Medtr3g025380.1 | E3 ubiquitin-protein ligase | 36.3 (M:36.3) | 5.0 | 16.5 | 9.6 | 1 | 234 |
| RK20_MARPO | 50S ribosomal protein L20, chloroplastic | 36.3 (M:36.3) | 9.5 | 13.6 | 12.3 | 1 | 235 |
| MES2_ARATH | Methylesterase 2 | 36.2 (M:36.2) | 5.7 | 29.6 | 5.1 | 1 | 236 |
| IMGA\|Medtr6g047090.1 | Unknown Protein | 36.0 (M:36.0) | 6.2 | 31.4 | 11.0 | 1 | 237 |
| IMGA\|Medtr3g046650.1 | Unknown Protein | 36.0 (M:36.0) | 6.8 | 28.1 | 4.5 | 1 | 238 |
| DYHG_CHLRE | Dynein gamma chain, flagellar outer arm | 35.8 (M:35.8) | 0.4 | 512.5 | 6.2 | 1 | 239 |
| IMGA\|Medtr7g069580.1 | TIR-NBS-LRR type disease resistance protein | 35.7 (M:35.7) | 1.3 | 138.2 | 6.1 | 1 | 240 |
| WOX1B_ORYSJ | WUSCHEL-related homeobox 1B | 35.6 (M:35.6) | 8.0 | 30.7 | 7.9 | 1 | 241 |
| IMGA\|Medtr2g012830.1 | Helicase-like transcription factor | 35.5 (M:35.5) | 1.1 | 145.3 | 4.8 | 1 | 242 |
| RK2_PHYPA | Chloroplast 50S ribosomal protein L2 | 35.4 (M:35.4) | 7.6 | 30.7 | 11.9 | 1 | 243 |
| GDL81_ARATH | GDSL esterase/lipase | 35.4 (M:35.4) | 6.8 | 40.7 | 5.9 | 1 | 244 |
| IMGA\|Medtr6g023750.1 | Triacylglycerol lipase 2 | 35.4 (M:35.4) | 4.5 | 40.4 | 9.4 | 1 | 245 |
| RPOB_PANGI | DNA-directed RNA polymerase beta chain (EC 2.7.7.6) (PEP) | 35.4 (M:35.4) | 1.9 | 120.8 | 9.4 | 1 | 246 |
| CCD21_ARATH | Cyclin-D2-1 OS=Arabidopsis thaliana | 35.3 (M:35.3) | 8.0 | 40.6 | 4.8 | 1 | 247 |
| AL7A1_PEA | Aldehyde dehydrogenase family 7 member | 35.3 (M:35.3) | 3.9 | 53.8 | 5.5 | 1 | 248 |
| IMGA\|Medtr7g011590.1 | Heat shock 70 kDa protein | 35.2 (M:35.2) | 2.5 | 66.8 | 5.9 | 1 | 249 |
| BH139_ARATH | Transcription factor bHLH139 | 35.2 (M:35.2) | 5.8 | 25.1 | 6.6 | 1 | 250 |
| VSR1_ARATH | Vacuolar-sorting receptor 1 | 35.2 (M:35.2) | 5.9 | 68.9 | 5.9 | 1 | 251 |
| IMGA\|Medtr3g069160.1 | Folylpolyglutamate synthase | 35.0 (M:35.0) | 6.5 | 61.8 | 6.1 | 1 | 252 |
| TPS9_RICCO | Probable terpene synthase 9 | 35.0 (M:35.0) | 2.7 | 67.7 | 5.8 | 1 | 253 |

**M. orbicularis 18 d**

| Accession | Protein | Scores | S.Coverage | MW [kDa] | pI | Peptides | Rank |
|---|---|---|---|---|---|---|---|
| IMGA\|Medtr7g079730.1 | Convicilin (Fragment) | 1595.5 (M:1595.5) | 35.1 | 53.1 | 5.3 | 27 | 1 |
| IMGA\|Medtr7g079820.1 | Convicilin (Fragment) | 1178.8 (M:1178.8) | 41.8 | 53.7 | 5.6 | 6 | 2 |
| IMGA\|Medtr7g079780.1 | Convicilin (Fragment) | 1155.4 (M:1155.4) | 36.2 | 53.2 | 5.5 | 3 | 3 |
| IMGA\|Medtr1g072630.1 | Legumin B | 1135.1 (M:1135.1) | 35.9 | 65.3 | 6.1 | 17 | 4 |
| IMGA\|Medtr7g079770.1 | Provicilin (Fragment) | 984.9 (M:984.9) | 32 | 53.1 | 5.3 | 1 | 5 |
| IMGA\|Medtr1g072600.1 | Legumin B (Fragment) | 982.2 (M:982.2) | 34.6 | 67.2 | 6.5 | 5 | 6 |
| IMGA\|Medtr1g072630.2 | Legumin B (Fragment) | 767.5 (M:767.5) | 30.5 | 63.5 | 6.4 | 1 | 7 |
| IMGA\|Medtr1g072610.2 | Legumin B (Fragment) | 757.9 (M:757.9) | 30.1 | 63.4 | 6.4 | 1 | 8 |
| IMGA\|Medtr4g103920.1 | Glyceraldehyde-3-phosphate dehydrogenase (Fragment) | 500.1 (M:500.1) | 29.2 | 36.6 | 6.7 | 8 | 9 |
| IMGA\|Medtr2g083160.1 | Conglutin | 421.5 (M:421.5) | 25 | 16.8 | 6.3 | 8 | 10 |
| IMGA\|Medtr1g108770.2 | ATP synthase subunit beta | 418.2 (M:418.2) | 21.1 | 59.9 | 5.8 | 7 | 11 |
| VCLC_PEA | Vicilin precursor | 414.5 (M:414.5) | 10.5 | 52.2 | 5.3 | 2 | 12 |
| MDHC_MEDSA | Malate dehydrogenase | 387.4 (M:387.4) | 24.1 | 35.5 | 6.4 | 6 | 13 |
| IMGA\|Medtr1g083960.1 | Calreticulin | 378.4 (M:378.4) | 20.1 | 48.4 | 4.3 | 5 | 14 |
| G3PC_ANTMA | Glyceraldehyde-3-phosphate dehydrogenase | 375.0 (M:375.0) | 23.4 | 36.7 | 9 | 1 | 15 |
| IMGA\|Medtr1g043040.1 | Malate dehydrogenase | 369.8 (M:369.8) | 27.7 | 35.5 | 6.1 | 1 | 16 |
| IMGA\|Medtr3g085850.1 | Glyceraldehyde 3-phosphate dehydrogenase | 357.9 (M:357.9) | 35.9 | 37 | 7.7 | 3 | 17 |
| RBL_HEDHE | Ribulose bisphosphate carboxylase large chain | 357.4 (M:357.4) | 11.6 | 51.5 | 6.3 | 5 | 18 |
| IMGA\|Medtr2g066120.1 | Phosphoglycerate kinase | 355.4 (M:355.4) | 23.7 | 42.4 | 5.7 | 6 | 19 |
| PDI_MEDSA | Protein disulfide-isomerase precursor (EC 5.3.4.1) | 343.5 (M:343.5) | 15.4 | 57.1 | 4.8 | 5 | 20 |
| IMGA\|Medtr1g023120.1 | Beta-galactosidase | 342.5 (M:342.5) | 13.6 | 83.1 | 9.2 | 7 | 21 |
| PGKH2_ARATH | Phosphoglycerate kinase 2 | 326.0 (M:326.0) | 14.2 | 49.9 | 9.1 | 1 | 22 |
| IMGA\|Medtr5g019780.1 | Vicilin-like antimicrobial peptides 2-3 (Fragment) | 303.9 (M:303.9) | 12.9 | 86.4 | 5.2 | 5 | 23 |
| BIP_SOLLC | Luminal-binding protein precursor (BiP) | 303.5 (M:303.5) | 10.4 | 73.2 | 5 | 5 | 24 |
| HSP7E_SPIOL | Chloroplast envelope membrane 70 kDa heat shock-related | 296.3 (M:296.3) | 11.5 | 71.7 | 5.2 | 3 | 25 |
| IMGA\|Medtr5g096430.1 | Heat shock protein 90 (Fragment) | 278.6 (M:278.6) | 9.2 | 80.1 | 4.8 | 5 | 26 |
| EF1A_MAIZE | Elongation factor 1-alpha (EF-1-alpha) | 278.0 (M:278.0) | 13.6 | 49.2 | 9.8 | 6 | 27 |
| IMGA\|Medtr5g064580.1 | 14-3-3 protein (Fragment) | 261.0 (M:261.0) | 35 | 29.2 | 4.5 | 5 | 28 |
| CALR_NICPL | Calreticulin | 252.5 (M:252.5) | 13.5 | 47.5 | 4.3 | 3 | 29 |
| TBA4_GOSHI | Tubulin alpha-4 chain (Alpha-4 tubulin) | 249.6 (M:249.6) | 15.3 | 49.5 | 4.8 | 4 | 30 |
| BIP3_MAIZE | Luminal-binding protein 3 precursor (BiP3) | 242.4 (M:242.4) | 8.7 | 73.1 | 5 | 1 | 31 |
| IMGA\|Medtr5g018940.1 | 40S ribosomal protein S4 | 221.1 (M:221.1) | 19.6 | 29.9 | 10.8 | 4 | 32 |
| IMGA\|Medtr2g008050.1 | Actin | 220.9 (M:220.9) | 18.8 | 41.6 | 5.2 | 4 | 33 |
| IMGA\|Medtr1g025430.1 | Endoplasmin homolog | 218.5 (M:218.5) | 7.1 | 94.1 | 4.6 | 4 | 34 |
| IMGA\|Medtr5g069050.1 | Fructose-bisphosphate aldolase | 210.4 (M:210.4) | 7.5 | 78.3 | 5.7 | 4 | 35 |
| IMGA\|Medtr2g029730.1 | Peroxidase | 203.8 (M:203.8) | 4.3 | 37.4 | 6.2 | 2 | 36 |
| ACT2_SOLLC | Actin-51 | 199.8 (M:199.8) | 21.1 | 37.2 | 5.2 | 1 | 37 |
| IMGA\|Medtr7g009330.1 | Outer plastidial membrane protein porin | 199.8 (M:199.8) | 26.4 | 29.6 | 9.4 | 4 | 38 |
| IMGA\|Medtr3g018780.1 | Annexin-like protein RJ4 | 194.2 (M:194.2) | 10.5 | 36.2 | 8.7 | 4 | 39 |
| ENO_RICCO | Enolase (EC 4.2.1.11) | 193.0 (M:193.0) | 7.4 | 47.9 | 5.5 | 3 | 40 |
| IMGA\|Medtr4g124660.2 | Sucrose synthase | 189.0 (M:189.0) | 8.3 | 92.2 | 5.8 | 4 | 41 |
| RS254_ARATH | 40S ribosomal protein S25-4 | 183.4 (M:183.4) | 19.4 | 12 | 11.2 | 3 | 42 |
| IMGA\|Medtr6g021800.1 | Elongation factor 1-alpha | 181.0 (M:181.0) | 5.8 | 109.7 | 9.8 | 1 | 43 |
| IMGA\|Medtr5g077000.2 | UTP-glucose 1 phosphate uridylyltransferase | 180.6 (M:180.6) | 16.2 | 51.4 | 5.1 | 4 | 44 |
| IMGA\|Medtr7g086300.2 | Methionine synthase | 175.3 (M:175.3) | 9.4 | 83.1 | 5.8 | 3 | 45 |
| IMGA\|Medtr7g113650.1 | Glucose and ribitol dehydrogenase homolog 1 | 171.8 (M:171.8) | 22.5 | 31.8 | 7.7 | 4 | 46 |
| IMGA\|Medtr2g099560.1 | Lipoxygenase | 168.8 (M:168.8) | 5.6 | 96.9 | 5.8 | 3 | 47 |
| IMGA\|Medtr3g088970.1 | Argininosuccinate synthase | 161.3 (M:161.3) | 10.3 | 52.7 | 6.3 | 3 | 48 |
| IMGA\|Medtr1g079530.1 | GDSL esterase/lipase | 160.8 (M:160.8) | 11.1 | 39.6 | 9.4 | 3 | 49 |
| IMGA\|Medtr2g005570.1 | Elongation factor 1-gamma | 156.8 (M:156.8) | 12.7 | 47.7 | 6.5 | 3 | 50 |
| IMGA\|Medtr4g075340.1 | Translocon-associated protein subunit alpha | 153.3 (M:153.3) | 13.7 | 27.5 | 4.6 | 3 | 51 |
| ATPAM_BETVU | ATP synthase subunit alpha, mitochondrial | 152.4 (M:152.4) | 7.7 | 54.9 | 6 | 3 | 52 |
| IMGA\|Medtr5g074860.1 | Peroxidase | 151.0 (M:151.0) | 10.5 | 35.7 | 10.4 | 3 | 53 |
| PSBP_PEA | Oxygen-evolving enhancer protein 2 | 150.6 (M:150.6) | 18.5 | 28 | 9.1 | 3 | 54 |
| PSBO_PEA | Oxygen-evolving enhancer protein 1 | 142.7 (M:142.7) | 17.3 | 34.9 | 6.3 | 3 | 55 |
| CALM_MALDO | Calmodulin | 137.1 (M:137.1) | 30.9 | 16.8 | 4 | 3 | 56 |
| PDIA6_MEDSA | Probable protein disulfide-isomerase A6 precursor (EC 5.3.4.1) | 135.8 (M:135.8) | 12.6 | 40.5 | 5.3 | 3 | 57 |
| H2B2_SOLLC | Histone H2B.2 | 135.1 (M:135.1) | 10.7 | 15.4 | 10.6 | 2 | 58 |
| IMGA\|Medtr5g033090.1 | 60S ribosomal protein L27a-3 | 132.9 (M:132.9) | 14 | 18.5 | 11 | 2 | 59 |
| ENO_SOLLC | Enolase | 129.6 (M:129.6) | 9 | 47.8 | 5.6 | 1 | 60 |
| IMGA\|Medtr6g087990.1 | Peroxiredoxin | 126.2 (M:126.2) | 12.3 | 17.5 | 5.5 | 1 | 61 |
| IMGA\|Medtr1g061670.1 | 60S ribosomal protein L24 | 125.5 (M:125.5) | 14.7 | 18.4 | 11.4 | 2 | 62 |
| IMGA\|Medtr1g090130.1 | Chaperonin CPN60-2 | 123.3 (M:123.3) | 9 | 61.1 | 6.3 | 2 | 63 |
| PSBB_CUCSA | Photosystem II CP47 chlorophyll apoprotein | 122.8 (M:122.8) | 4.7 | 55.9 | 6.3 | 2 | 64 |
| IMGA\|Medtr8g038210.1 | Annexin-like protein RJ4 | 122.8 (M:122.8) | 10.3 | 38.6 | 7.1 | 2 | 65 |
| ENO_ORYSJ | Enolase | 122.5 (M:122.5) | 5.6 | 47.9 | 5.3 | 2 | 66 |
| IMGA\|Medtr2g025120.1 | 1-aminocyclopropane-1-carboxylate oxidase (Fragment) | 117.7 (M:117.7) | 10.4 | 36.1 | 4.9 | 2 | 67 |
| IMGA\|Medtr2g039680.1 | Nucleosome assembly protein 1-like 1 | 116.8 (M:116.8) | 11.7 | 41.8 | 4.1 | 2 | 68 |
| IMGA\|Medtr4g061140.1 | Cytosolic ascorbate peroxidase | 115.0 (M:115.0) | 16.4 | 27.1 | 5.5 | 2 | 69 |
| IMGA\|Medtr1g064060.1 | Adenosine kinase 2 | 114.1 (M:114.1) | 13.5 | 37.6 | 4.9 | 2 | 70 |
| IMGA\|Medtr2g033930.1 | GDSL esterase/lipase | 111.3 (M:111.3) | 8.3 | 40 | 7.6 | 2 | 71 |
| IMGA\|Medtr3g098420.1 | Staphylococcal nuclease domain-containing protein 1 | 108.4 (M:108.4) | 4.5 | 108.2 | 6.6 | 2 | 72 |
| IMGA\|Medtr4g079780.1 | Ketol-acid reductoisomerase | 108.3 (M:108.3) | 5.5 | 62.9 | 6.3 | 2 | 73 |
| LEG_CICAR | Legumin | 108.2 (M:108.2) | 2.4 | 56.2 | 6.2 | 2 | 74 |
| IMGA\|Medtr8g012330.3 | Ribosomal protein S8 | 107.1 (M:107.1) | 10.9 | 16 | 11.6 | 1 | 75 |
| RUBB_PEA | RuBisCO large subunit-binding protein subunit beta | 106.4 (M:106.4) | 8.2 | 62.9 | 5.8 | 2 | 76 |
| IMGA\|Medtr3g093110.1 | Ribosomal protein L9 (Fragment) | 104.0 (M:104.0) | 15 | 21.7 | 9.9 | 2 | 77 |
| IMGA\|Medtr2g069050.1 | Elongation factor EF-2 (Fragment) | 103.5 (M:103.5) | 3.3 | 94.1 | 5.7 | 2 | 78 |
| TBB9_GOSHI | Tubulin beta-9 chain (Beta-9 tubulin) | 102.0 (M:102.0) | 11.5 | 49.9 | 4.6 | 2 | 79 |
| IMGA\|Medtr5g088660.1 | Elongation factor 1-beta | 101.3 (M:101.3) | 18.4 | 24.2 | 4.5 | 2 | 80 |
| IMGA\|Medtr3g114850.1 | Plastocyanin | 100.9 (M:100.9) | 28.1 | 17.1 | 4.8 | 2 | 81 |

| | | | | | | | |
|---|---|---|---|---|---|---|---|
| IMGA\|Medtr4g070140.1 | Glycine-rich RNA binding protein 1 | 100.8 (M:100.8) | 16.8 | 18.4 | 5.1 | 2 | 82 |
| IMGA\|Medtr1g045410.1 | 60S ribosomal protein L4 | 100.2 (M:100.2) | 7.1 | 44.7 | 10.9 | 2 | 83 |
| IMGA\|Medtr2g014030.1 | 40S ribosomal protein S6 | 100.0 (M:100.0) | 6 | 28.2 | 11.5 | 1 | 84 |
| IMGA\|Medtr8g023310.2 | Pectinesterase | 99.1 (M:99.1) | 3.6 | 43.1 | 9.6 | 1 | 85 |
| CP18D_ARATH | Peptidyl-prolyl cis-trans isomerase CYP18-4 (EC 5.2.1.8) | 99.0 (M:99.0) | 8.7 | 18.4 | 9.8 | 2 | 86 |
| IMGA\|Medtr7g111590.3 | 60S ribosomal protein L13 | 97.3 (M:97.3) | 23 | 15.7 | 10.8 | 2 | 87 |
| IMGA\|Medtr8g105340.1 | 40S ribosomal protein S2 | 95.0 (M:95.0) | 11.5 | 30.4 | 11 | 2 | 88 |
| GBLPA_ORYSJ | Guanine nucleotide-binding protein subunit | 94.3 (M:94.3) | 8.1 | 36.2 | 6 | 1 | 89 |
| IMGA\|Medtr7g118060.1 | 60s acidic ribosomal protein P1 | 94.0 (M:94.0) | 11.5 | 11.4 | 4 | 1 | 90 |
| IMGA\|Medtr2g064660.2 | Photosystem II 10 kDa polypeptide | 93.7 (M:93.7) | 18.1 | 13.6 | 10.2 | 2 | 91 |
| TBB1_AVESA | Tubulin beta-1 chain (Beta-1 tubulin) | 92.7 (M:92.7) | 13.2 | 43.3 | 4.4 | 1 | 92 |
| IMGA\|Medtr8g067080.1 | ADP-ribosylation factor GTPase-activating protein AGD10 | 90.6 (M:90.6) | 6.6 | 43.9 | 9.7 | 1 | 93 |
| PSBQ_ONOVI | Oxygen-evolving enhancer protein 3 | 86.8 (M:86.8) | 5.6 | 24.8 | 10 | 1 | 94 |
| IMGA\|Medtr2g089860.1 | Subtilisin-like protease | 86.8 (M:86.8) | 5.5 | 81.8 | 8.7 | 2 | 95 |
| IMGA\|Medtr2g034720.1 | Beta xylosidase | 86.4 (M:86.4) | 3.5 | 82.6 | 9.6 | 2 | 96 |
| IMGA\|Medtr1g081410.1 | 40S ribosomal protein S24 | 84.7 (M:84.7) | 10.9 | 15.7 | 11.1 | 1 | 97 |
| IMGA\|Medtr3g092600.1 | 60S acidic ribosomal protein P1 | 84.3 (M:84.3) | 14.5 | 11.1 | 4.1 | 1 | 98 |
| RAN_POPEU | GTP-binding nuclear protein Ran (Fragments) | 84.2 (M:84.2) | 50 | 3.3 | 9.5 | 2 | 99 |
| IMGA\|Medtr4g063060.1 | 60S ribosomal protein L23a | 84.1 (M:84.1) | 8.6 | 17.1 | 10.7 | 1 | 100 |
| IMGA\|Medtr2g038250.1 | 60S ribosomal protein L7-4 | 83.8 (M:83.8) | 6.1 | 28.5 | 10.4 | 1 | 101 |
| CPNA1_ARATH | Chaperonin 60 subunit alpha 1 | 82.6 (M:82.6) | 5.1 | 62 | 4.9 | 2 | 102 |
| IMGA\|Medtr6g055020.1 | ATP synthase subunit beta, chloroplastic | 82.0 (M:82.0) | 8.9 | 30.1 | 6.2 | 1 | 103 |
| IMGA\|Medtr7g077880.1 | Unknown Protein | 81.4 (M:81.4) | 4.5 | 30.4 | 4.9 | 1 | 104 |
| IMGA\|Medtr8g081490.1 | Adenosylhomocysteinase | 81.2 (M:81.2) | 11.5 | 49.8 | 5.2 | 2 | 105 |
| SODM_PRUPE | Superoxide dismutase [Mn] | 79.9 (M:79.9) | 6.6 | 25.4 | 9.1 | 1 | 106 |
| IMGA\|Medtr4g085540.1 | Poly(A)-binding protein | 78.8 (M:78.8) | 2.6 | 71 | 9 | 1 | 107 |
| IMGA\|Medtr5g076080.3 | Cbs domain protein (Fragment) | 78.6 (M:78.6) | 18 | 22.4 | 9.8 | 2 | 108 |
| LEGJ_PEA | Legumin J precursor | 78.6 (M:78.6) | 5.4 | 56.9 | 5.6 | 1 | 109 |
| IMGA\|Medtr5g098060.1 | Fasciclin-like arabinogalactan protein 2 | 78.1 (M:78.1) | 6.9 | 27.8 | 5 | 1 | 110 |
| IMGA\|Medtr7g112880.2 | 60S ribosomal protein L18-2 | 74.8 (M:74.8) | 10.4 | 14.8 | 11.5 | 1 | 111 |
| IMGA\|Medtr3g088160.3 | Ascorbate peroxidase | 74.2 (M:74.2) | 11.9 | 42.1 | 9.3 | 2 | 112 |
| IMGA\|Medtr7g116350.1 | Class III acidic chitinase | 74.0 (M:74.0) | 5.4 | 94.4 | 9.1 | 1 | 113 |
| P2C66_ARATH | Probable protein phosphatase 2C | 73.5 (M:73.5) | 3.3 | 74.8 | 5.8 | 2 | 114 |
| IMGA\|Medtr4g059400.1 | 60S ribosomal protein L12 | 73.5 (M:73.5) | 9 | 17.8 | 9.7 | 1 | 115 |
| IMGA\|Medtr4g070080.2 | Glycine-rich RNA-binding protein | 72.5 (M:72.5) | 33.3 | 5.7 | 4.3 | 1 | 116 |
| G3PA_TOBAC | Glyceraldehyde-3-phosphate dehydrogenase A | 70.6 (M:70.6) | 6.4 | 41.8 | 6.7 | 1 | 117 |
| IMGA\|Medtr4g060780.2 | Allergen Gly m Bd (Fragment) | 67.8 (M:67.8) | 6.3 | 24.1 | 6.2 | 1 | 118 |
| IMGA\|Medtr4g059720.1 | Fasciclin-like arabinogalactan protein 12 | 67.4 (M:67.4) | 3.6 | 26.9 | 9.9 | 1 | 119 |
| IMGA\|Medtr8g091910.1 | 60S ribosomal protein L6 | 67.3 (M:67.3) | 6.4 | 24.4 | 10.5 | 1 | 120 |
| IMGA\|Medtr5g083170.1 | Ferritin-2 | 66.3 (M:66.3) | 5.6 | 27.9 | 5.7 | 1 | 121 |
| IMGA\|Medtr7g006560.1 | Transaldolase | 66.3 (M:66.3) | 4.1 | 48.5 | 6.2 | 1 | 122 |
| G3PP1_ARATH | Glyceraldehyde 3-phosphate dehydrogenase | 66.2 (M:66.2) | 4.7 | 44.8 | 9.4 | 1 | 123 |
| IMGA\|Medtr7g052690.1 | Early tobacco anther 1 | 65.9 (M:65.9) | 13.9 | 16.9 | 4.5 | 1 | 124 |
| IMGA\|Medtr7g092720.1 | 40S ribosomal protein S20-2 | 65.8 (M:65.8) | 9.8 | 13.7 | 10.2 | 1 | 125 |
| IMGA\|Medtr1g092670.1 | Copper chaperone | 65.3 (M:65.3) | 15.2 | 8.4 | 7.1 | 1 | 126 |
| RS271_ARATH | 40S ribosomal protein S27-1 | 64.4 (M:64.4) | 20.2 | 9.4 | 10.6 | 1 | 127 |
| NDK1_PSEMZ | Nucleoside diphosphate kinase 1 (Fragments) | 64.1 (M:64.1) | 50 | 1.9 | 4.4 | 1 | 128 |
| IMGA\|Medtr4g021310.1 | 40S ribosomal protein S9 | 63.6 (M:63.6) | 23.3 | 8 | 9.8 | 1 | 129 |
| IMGA\|AC146630_2.1 | 2-cys peroxiredoxin BAS1 | 63.3 (M:63.3) | 6.4 | 29 | 6.1 | 1 | 130 |
| IMGA\|Medtr7g110660.1 | Bifunctional aminoacyl-tRNA synthetase | 62.3 (M:62.3) | 2.6 | 60 | 6.1 | 1 | 131 |
| IMGA\|Medtr6g018300.1 | Ribulose bisphosphate carboxylase small chain (Fragment) | 61.8 (M:61.8) | 7.9 | 19.8 | 9.4 | 1 | 132 |
| RS5_NICPL | 40S ribosomal protein S5 (Fragment) | 61.4 (M:61.4) | 9.7 | 17.1 | 11.1 | 1 | 133 |
| IMGA\|Medtr6g005820.3 | ADP-ribosylation factor | 60.3 (M:60.3) | 12.8 | 18.7 | 5.5 | 1 | 134 |
| IMGA\|Medtr3g085210.1 | Non-specific lipid-transfer protein | 59.5 (M:59.5) | 7.8 | 19.9 | 9.4 | 1 | 135 |
| IMGA\|Medtr1g100960.3 | 60S ribosomal protein L36 | 59.4 (M:59.4) | 10.8 | 12.4 | 12 | 1 | 136 |
| IMGA\|Medtr5g062570.1 | Auxin-induced beta-glucosidase | 59.1 (M:59.1) | 11.5 | 21.1 | 9.7 | 1 | 137 |
| IMGA\|Medtr3g088040.1 | Photosystem II 22 kDa protein | 59.0 (M:59.0) | 5.9 | 28.9 | 7.3 | 1 | 138 |
| IMGA\|Medtr4g071000.1 | Nascent polypeptide-associated complex subunit beta | 58.1 (M:58.1) | 12.5 | 16.5 | 5.7 | 1 | 139 |
| IMGA\|Medtr4g078410.1 | Early nodulin-like protein 2 | 57.5 (M:57.5) | 8.6 | 20.6 | 9.2 | 1 | 140 |
| IMGA\|Medtr4g063550.1 | THO complex subunit 4 | 57.4 (M:57.4) | 9 | 40.8 | 9.9 | 1 | 141 |
| PSBC_PLAOC | Photosystem II CP43 chlorophyll apoprotein | 57.3 (M:57.3) | 3 | 50.2 | 6.2 | 1 | 142 |
| IMGA\|Medtr5g014960.1 | Branched-chain-amino-acid aminotransferase | 57.0 (M:57.0) | 5.9 | 44.2 | 7.5 | 1 | 143 |
| IMGA\|Medtr4g070190.1 | Glycine-rich RNA binding protein 1 | 56.5 (M:56.5) | 7.3 | 16.4 | 5.1 | 1 | 144 |
| IMGA\|Medtr7g101870.1 | Cell division control protein 48 homolog E | 56.5 (M:56.5) | 4.2 | 38.9 | 4.6 | 1 | 145 |
| IMGA\|Medtr6g021670.1 | 40S ribosomal protein S7-like protein | 55.4 (M:55.4) | 13.6 | 21.9 | 10.3 | 1 | 146 |
| IMGA\|AC235665_13.2 | 40S ribosomal protein S3a | 54.5 (M:54.5) | 8.8 | 18.4 | 10.1 | 1 | 147 |
| IMGA\|Medtr4g070600.1 | 40S ribosomal protein S25-2 | 54.5 (M:54.5) | 9.3 | 11.8 | 11.2 | 1 | 148 |
| IMGA\|Medtr1g094630.1 | 60S ribosomal protein L4 | 53.6 (M:53.6) | 1.7 | 130.9 | 6.5 | 1 | 149 |
| IMGA\|AC235753_1.1 | Cysteine proteinase | 53.4 (M:53.4) | 6.6 | 40.3 | 6.1 | 1 | 150 |
| ZB14_BRAJU | 14 kDa zinc-binding protein (Protein kinase C inhibitor) (PKCI) | 53.1 (M:53.1) | 15 | 12.6 | 6.6 | 1 | 151 |
| IMGA\|Medtr5g091120.2 | 60S ribosomal protein L18a | 51.4 (M:51.4) | 9.5 | 17.5 | 11 | 1 | 152 |
| IMGA\|Medtr5g045310.1 | Unknown Protein | 51.2 (M:51.2) | 21.2 | 10.1 | 9.7 | 1 | 153 |
| IMGA\|Medtr5g011850.1 | EF hand family protein | 50.6 (M:50.6) | 10.9 | 16.6 | 4.5 | 1 | 154 |
| IMGA\|Medtr3g057190.1 | (+)-neomenthol dehydrogenase | 50.6 (M:50.6) | 3.7 | 31.9 | 5.4 | 1 | 155 |
| IMGA\|Medtr4g116010.1 | Flavonol synthase/flavanone 3-hydroxylase | 50.5 (M:50.5) | 4.3 | 39.8 | 4.9 | 1 | 156 |
| IMGA\|Medtr7g075270.1 | Fasciclin-like arabinogalactan protein 13 | 50.3 (M:50.3) | 4.1 | 42.5 | 5.8 | 1 | 157 |
| IMGA\|Medtr4g130860.1 | Leucine aminopeptidase 2, chloroplastic | 50.2 (M:50.2) | 3.7 | 59.6 | 8.6 | 1 | 158 |
| CP19D_ARATH | Peptidyl-prolyl cis-trans isomerase CYP19-4 | 49.4 (M:49.4) | 5.5 | 21.5 | 9.6 | 1 | 159 |
| PP318_ARATH | Putative pentatricopeptide repeat-containing protein | 49.1 (M:49.1) | 4.8 | 52.8 | 9.7 | 1 | 160 |
| IMGA\|Medtr5g008210.1 | Unknown Protein | 48.5 (M:48.5) | 17.6 | 14 | 6.2 | 1 | 161 |
| IMGA\|Medtr7g021680.1 | Aldo-keto reductase yakc | 48.2 (M:48.2) | 4.1 | 37.4 | 5.7 | 1 | 162 |
| PORA_CUCSA | Protochlorophyllide reductase | 48.2 (M:48.2) | 4.5 | 43 | 9.8 | 1 | 163 |
| IMGA\|Medtr8g076770.1 | Protein transport protein Sec61 subunit alpha | 48.1 (M:48.1) | 1.9 | 52.2 | 9.7 | 1 | 164 |
| UGPA_PYRPY | UTP--glucose-1-phosphate uridylyltransferase (EC 2.7.7.9) | 48.0 (M:48.0) | 1.9 | 51.8 | 6 | 1 | 165 |

| | | | | | | | |
|---|---|---|---|---|---|---|---|
| PSA7_CICAR | Proteasome subunit alpha type 7 (EC 3.4.25.1) | 47.9 (M:47.9) | 10 | 27.1 | 7.7 | 1 | 166 |
| IMGA\|Medtr5g033920.1 | ATP-citrate synthase | 47.6 (M:47.6) | 5.7 | 46.6 | 5.5 | 1 | 167 |
| FKB15_VICFA | FK506-binding protein 2 | 47.2 (M:47.2) | 8.6 | 16.2 | 7.6 | 1 | 168 |
| IMGA\|Medtr2g098010.1 | Proteasome subunit beta type | 46.3 (M:46.3) | 11.3 | 25.4 | 6.3 | 1 | 169 |
| IMGA\|Medtr5g091930.1 | Citrate synthase | 46.1 (M:46.1) | 2.7 | 56.6 | 9.1 | 1 | 170 |
| IMGA\|Medtr7g055500.1 | Unknown Protein | 45.7 (M:45.7) | 1.2 | 56.5 | 6.1 | 1 | 171 |
| H2A3_VOLCA | Histone H2A-III | 45.6 (M:45.6) | 7 | 13.5 | 10.7 | 1 | 172 |
| IMGA\|Medtr1g106900.1 | 40S ribosomal protein S18 | 45.4 (M:45.4) | 13.2 | 17.6 | 11.3 | 1 | 173 |
| STIL2_ARATH | Protein STICHEL-like 2 | 44.9 (M:44.9) | 1.4 | 93.8 | 9 | 1 | 174 |
| IMGA\|Medtr1g110160.1 | Receptor-like protein kinase | 44.6 (M:44.6) | 2.6 | 65.5 | 9.1 | 1 | 175 |
| IMGA\|Medtr7g101580.1 | Cell division control protein 48 homolog E | 44.6 (M:44.6) | 9.5 | 26.8 | 10.3 | 1 | 176 |
| EF1B_ORYSJ | Elongation factor 1-beta | 44.6 (M:44.6) | 7.6 | 23.8 | 4.5 | 1 | 177 |
| IMGA\|Medtr2g096660.1 | UDP-glucuronic acid decarboxylase 3 | 44.5 (M:44.5) | 4 | 39.6 | 6.9 | 1 | 178 |
| IMGA\|Medtr6g072450.1 | Resistance-gene protein (Fragment) | 44.3 (M:44.3) | 1.3 | 128.1 | 6.4 | 1 | 179 |
| IMGA\|Medtr8g088370.1 | Chloroplast protein import component Toc159-like (Fragment) | 44.2 (M:44.2) | 1.5 | 149.3 | 4.1 | 1 | 180 |
| IMGA\|AC233651_1010.1 | Expressed protein (Fragment) | 43.9 (M:43.9) | 6.9 | 41.6 | 10.4 | 1 | 181 |
| IMGA\|Medtr4g132270.2 | Lactoylglutathione lyase | 43.8 (M:43.8) | 6.6 | 27 | 4.8 | 1 | 182 |
| IMGA\|Medtr8g075330.1 | Lysosomal alpha-mannosidase | 43.4 (M:43.4) | 1.5 | 114.1 | 6.9 | 1 | 183 |
| IMGA\|Medtr2g012110.1 | 60S ribosomal protein L26-1 | 43.0 (M:43.0) | 8.2 | 16.7 | 11.6 | 1 | 184 |
| LTL1_ARATH | GDSL esterase/lipase | 43.0 (M:43.0) | 6.8 | 40.1 | 5.5 | 1 | 185 |
| ATPA_ZYGCR | ATP synthase subunit alpha | 42.8 (M:42.8) | 3.2 | 54.8 | 5.2 | 1 | 186 |
| ADHX_PEA | Alcohol dehydrogenase class 3 (EC 1.1.1.1) | 42.8 (M:42.8) | 2.9 | 40.5 | 6.3 | 1 | 187 |
| IMGA\|Medtr7g113470.1 | T-complex protein 1 subunit beta | 42.5 (M:42.5) | 4.2 | 56.9 | 5.3 | 1 | 188 |
| RS172_ARATH | 40S ribosomal protein S17-2 | 42.4 (M:42.4) | 8.6 | 15.9 | 10.5 | 1 | 189 |
| IMGA\|Medtr5g084930.1 | Protein disulfide isomerase family | 42.2 (M:42.2) | 3.2 | 47.4 | 5.3 | 1 | 190 |
| IMGA\|Medtr8g072000.1 | Protein notum homolog | 42.1 (M:42.1) | 4.3 | 43.5 | 9.8 | 1 | 191 |
| FLA15_ARATH | Fasciclin-like arabinogalactan protein 15 | 42.0 (M:42.0) | 4.6 | 48 | 6.2 | 1 | 192 |
| IMGA\|Medtr6g047090.1 | Unknown Protein | 42.0 (M:42.0) | 6.2 | 31.4 | 11 | 1 | 193 |
| IMGA\|Medtr8g027080.1 | Cytochrome b5 | 41.6 (M:41.6) | 16.9 | 15.6 | 4.7 | 1 | 194 |
| IMGA\|Medtr7g109100.1 | Unknown Protein | 41.4 (M:41.4) | 17.2 | 16.5 | 11.2 | 1 | 195 |
| IMGA\|Medtr2g103730.1 | Phospholipase D alpha 1 | 41.2 (M:41.2) | 3 | 92 | 5.4 | 1 | 196 |
| PDI14_ARATH | Protein disulfide isomerase-like 1-4 | 41.2 (M:41.2) | 2.5 | 66.3 | 4.3 | 1 | 197 |
| RH53_ARATH | DEAD-box ATP-dependent RNA helicase 53 | 41.1 (M:41.1) | 3.2 | 65.3 | 9.9 | 1 | 198 |
| RSSA2_ARATH | 40S ribosomal protein Sa-2 | 41.0 (M:41.0) | 2.9 | 30.6 | 4.9 | 1 | 199 |
| IMGA\|Medtr7g098980.1 | Unknown Protein | 41.0 (M:41.0) | 4.9 | 39.7 | 10.1 | 1 | 200 |
| GSA_SOYBN | Glutamate-1-semialdehyde 2,1-aminomutase | 40.8 (M:40.8) | 4.5 | 49.6 | 5.6 | 1 | 201 |
| IMGA\|Medtr3g086150.2 | Myosin-like protein | 40.5 (M:40.5) | 0.8 | 201.8 | 4.5 | 1 | 202 |
| IMGA\|AC233577_36.1 | Coatomer subunit delta | 40.3 (M:40.3) | 2.5 | 58.2 | 5.4 | 1 | 203 |
| IMGA\|Medtr4g128840.2 | Xylose isomerase | 40.2 (M:40.2) | 5.6 | 23.7 | 4.9 | 1 | 204 |
| IMGA\|Medtr5g038160.1 | Unknown Protein | 40.1 (M:40.1) | 13.4 | 17.9 | 10.1 | 1 | 205 |
| COX2_ARATH | Cytochrome c oxidase subunit 2 | 39.9 (M:39.9) | 3.8 | 29.7 | 4.9 | 1 | 206 |
| IMGA\|Medtr3g087590.3 | L-myo inositol-1 phosphate synthase 1 | 39.8 (M:39.8) | 4.4 | 47.3 | 5.3 | 1 | 207 |
| IMGA\|Medtr7g080090.1 | 60S ribosomal protein L35 | 39.8 (M:39.8) | 10.6 | 14.3 | 11.4 | 1 | 208 |
| IMGA\|Medtr8g073730.1 | TMV resistance protein N | 39.6 (M:39.6) | 1.1 | 122.1 | 6.5 | 1 | 209 |
| IMGA\|Medtr6g084450.2 | Proteasome subunit beta type | 39.6 (M:39.6) | 6.8 | 23.3 | 9.5 | 1 | 210 |
| IMGA\|Medtr2g099950.1 | Beta-fructofuranosidase | 39.6 (M:39.6) | 1.7 | 64.8 | 9.5 | 1 | 211 |
| IF413_TOBAC | Eukaryotic initiation factor 4A-13 | 39.4 (M:39.4) | 4.5 | 40.2 | 5 | 1 | 212 |
| PROF_PRUPE | Profilin | 39.4 (M:39.4) | 7.6 | 14 | 4.6 | 1 | 213 |
| EF1D1_ARATH | Elongation factor 1-delta 1 | 39.3 (M:39.3) | 12.6 | 25.1 | 4.3 | 1 | 214 |
| IMGA\|Medtr4g024550.1 | 40S ribosomal protein S13 | 38.9 (M:38.9) | 9.4 | 15.8 | 10.8 | 1 | 215 |
| HIS6A_ARATH | Histidinol-phosphate aminotransferase 1 | 38.8 (M:38.8) | 5.8 | 46.6 | 5.7 | 1 | 216 |
| IMGA\|Medtr3g106580.1 | Protease inhibitor | 38.7 (M:38.7) | 2.5 | 103.5 | 9.6 | 1 | 217 |
| PP412_ARATH | Pentatricopeptide repeat-containing protein | 38.7 (M:38.7) | 4.4 | 59.9 | 9.6 | 1 | 218 |
| ILL1_ORYSJ | IAA-amino acid hydrolase ILR1-like 1 | 38.7 (M:38.7) | 4.8 | 47.1 | 5.6 | 1 | 219 |
| IMGA\|Medtr7g113420.1 | Protein AUXIN RESPONSE 4 | 38.7 (M:38.7) | 4.3 | 55.2 | 5.9 | 1 | 220 |
| UN03_PINPS | Unknown protein from 2D-PAGE of needles (Fragments) | 38.6 (M:38.6) | 34 | 5.8 | 4.2 | 1 | 221 |
| IMGA\|Medtr2g042330.2 | Aldehyde dehydrogenase family 7 member A1 | 38.3 (M:38.3) | 5.1 | 54.2 | 5.8 | 1 | 222 |
| IMGA\|Medtr2g064680.2 | Unknown Protein | 38.3 (M:38.3) | 15.4 | 10.5 | 5.7 | 1 | 223 |
| LOR1_ARATH | Protein LURP-one-related 1 | 38.2 (M:38.2) | 8.9 | 25.4 | 9.4 | 1 | 224 |
| Y4193_ARATH | Uncharacterized protein | 38.1 (M:38.1) | 2 | 108 | 5.6 | 1 | 225 |
| LEGU_CANEN | Legumin | 38.1 (M:38.1) | 4.2 | 52.7 | 5.8 | 1 | 226 |
| IMGA\|Medtr7g092970.1 | Metal tolerance protein | 37.6 (M:37.6) | 12.8 | 20.2 | 9.9 | 1 | 227 |
| IMGA\|Medtr2g099570.1 | Seed lipoxygenase-3 | 37.6 (M:37.6) | 1.7 | 97.3 | 6.1 | 1 | 228 |
| SPD1_PEA | Spermidine synthase 1 | 37.5 (M:37.5) | 3.9 | 36.7 | 4.8 | 1 | 229 |
| IMGA\|Medtr4g114770.1 | Methyl binding domain protein (Fragment) | 37.4 (M:37.4) | 4.2 | 38.9 | 4.5 | 1 | 230 |
| IMGA\|Medtr4g057240.1 | Superoxide dismutase | 37.3 (M:37.3) | 11.9 | 20.7 | 6 | 1 | 231 |
| TIC40_PEA | Protein TIC 40 | 37.2 (M:37.2) | 4.6 | 47.1 | 5.7 | 1 | 232 |
| IMGA\|Medtr4g087490.1 | Cysteine synthase | 37.0 (M:37.0) | 5.5 | 39 | 9.1 | 1 | 233 |
| IMGA\|Medtr7g061100.1 | Non-S F-box protein 1 | 37.0 (M:37.0) | 8.4 | 37.6 | 9.8 | 1 | 234 |
| EXS_ARATH | Leucine-rich repeat receptor protein kinase EXS precursor (EC 2.7.11.1) | 37.0 (M:37.0) | 1.7 | 129.7 | 5.5 | 1 | 235 |
| K125_TOBAC | 125 kDa kinesin-related protein | 36.8 (M:36.8) | 1.9 | 113.6 | 5.5 | 1 | 236 |
| IMGA\|Medtr6g082370.1 | Unknown Protein | 36.8 (M:36.8) | 6.3 | 28.4 | 10 | 1 | 237 |
| HSP7S_SPIOL | Stromal 70 kDa heat shock-related protein | 36.7 (M:36.7) | 3.2 | 64.9 | 4.7 | 1 | 238 |
| HKT4_ORYSJ | Cation transporter | 36.7 (M:36.7) | 3.6 | 61.8 | 9.8 | 1 | 239 |
| IMGA\|Medtr3g101230.1 | Gamma-tubulin complex component 4 | 36.6 (M:36.6) | 3 | 121.4 | 6.5 | 1 | 240 |
| CAPP3_SORBI | Phosphoenolpyruvate carboxylase 3 (EC 4.1.1.31) | 36.6 (M:36.6) | 0.9 | 108.3 | 5.9 | 1 | 241 |
| IMGA\|Medtr1g044370.1 | Unknown Protein | 36.6 (M:36.6) | 4.3 | 54.8 | 5.2 | 1 | 242 |
| IMGA\|Medtr1g024890.1 | GDSL esterase/lipase | 36.6 (M:36.6) | 3.8 | 40.7 | 6.2 | 1 | 243 |
| IMGA\|Medtr2g009330.1 | Pyruvate decarboxylase isozyme 2 | 36.6 (M:36.6) | 2.1 | 68 | 6 | 1 | 244 |
| IMGA\|Medtr4g076620.1 | Unknown Protein | 36.6 (M:36.6) | 2.4 | 67.4 | 9.6 | 1 | 245 |
| IMGA\|Medtr3g098430.1 | Calnexin homolog | 36.4 (M:36.4) | 2.2 | 61.7 | 4.6 | 1 | 246 |
| MATK_THECC | Maturase K (Intron maturase) | 36.4 (M:36.4) | 2.8 | 59.4 | 9.9 | 1 | 247 |
| IMGA\|Medtr1g098170.1 | 40S ribosomal protein S18 | 36.4 (M:36.4) | 13.2 | 17.6 | 11.3 | 1 | 248 |
| IMGA\|Medtr4g101650.1 | Monocopper oxidase-like protein SKU5 | 36.4 (M:36.4) | 2.7 | 66.2 | 9.4 | 1 | 249 |

| | | | | | | | |
|---|---|---|---|---|---|---|---|
| EXL3_ARATH | GDSL esterase/lipase | 36.3 (M:36.3) | 5.5 | 39.8 | 5.5 | 1 | 250 |
| IMGA\|Medtr5g057990.1 | Alpha-D-xylosidase | 36.2 (M:36.2) | 1.8 | 102.9 | 6.3 | 1 | 251 |
| IMGA\|Medtr3g094710.1 | Leucine-rich repeat receptor-like protein kinase (Fragment) | 36.2 (M:36.2) | 2.4 | 117 | 6.3 | 1 | 252 |
| CSK2D_ARATH | Casein kinase II subunit beta-3 | 36.2 (M:36.2) | 5.4 | 30.8 | 4.9 | 1 | 253 |
| IMGA\|Medtr4g036400.1 | Polyphenol oxidase | 36.2 (M:36.2) | 4 | 54.5 | 7.8 | 1 | 254 |
| IMGA\|Medtr1g088450.1 | 60S ribosomal protein L22-like | 36.2 (M:36.2) | 7.6 | 13.6 | 10 | 1 | 255 |
| ATPB_TROAR | ATP synthase subunit beta (EC 3.6.3.14) | 36.1 (M:36.1) | 4.4 | 53.6 | 5.1 | 1 | 256 |
| CAPP1_MAIZE | Phosphoenolpyruvate carboxylase 1 (EC 4.1.1.31) | 36.1 (M:36.1) | 2 | 109.2 | 5.7 | 1 | 257 |
| IMGA\|Medtr4g028010.1 | Expansin-B4 | 35.9 (M:35.9) | 32.1 | 9.3 | 11.5 | 1 | 258 |
| IMGA\|Medtr1g005300.1 | 26S proteasome subunit | 35.9 (M:35.9) | 18.4 | 9.7 | 10 | 1 | 259 |
| IMGA\|Medtr8g005980.1 | Malate dehydrogenase | 35.9 (M:35.9) | 3.9 | 45.9 | 6.2 | 1 | 260 |
| WNK8_ARATH | Serine/threonine-protein kinase | 35.8 (M:35.8) | 3 | 63.8 | 4.9 | 1 | 261 |
| MYO9_ARATH | Myosin-9 | 35.7 (M:35.7) | 1 | 174.5 | 9.8 | 1 | 262 |
| H4_ARATH | Histone H4 | 35.7 (M:35.7) | 6.8 | 11.4 | 12 | 1 | 263 |
| IMGA\|Medtr4g075290.1 | Peptidyl-prolyl cis-trans isomerase | 35.7 (M:35.7) | 15.7 | 18.2 | 9.5 | 1 | 264 |
| FBK69_ARATH | F-box/kelch-repeat protein | 35.6 (M:35.6) | 5.2 | 42.6 | 5.4 | 1 | 265 |
| IMGA\|Medtr3g025380.1 | E3 ubiquitin-protein ligase | 35.6 (M:35.6) | 5 | 16.5 | 9.6 | 1 | 266 |
| CB4A_ARATH | Chlorophyll a-b binding protein CP29.1 | 35.6 (M:35.6) | 5.2 | 31.1 | 5.7 | 1 | 267 |
| CAF1J_ARATH | Probable CCR4-associated factor 1 homolog 10 | 35.6 (M:35.6) | 2.2 | 31.5 | 4.8 | 1 | 268 |
| IMGA\|Medtr4g035480.1 | ATP-dependent DNA helicase PIF1 | 35.5 (M:35.5) | 19 | 13.2 | 10.7 | 1 | 269 |
| Y3216_ORYSJ | Putative B3 domain-containing protein | 35.5 (M:35.5) | 1.3 | 118.1 | 10.3 | 1 | 270 |
| CATA4_SOYBN | Catalase-4 (EC 1.11.1.6) | 35.4 (M:35.4) | 2.6 | 56.7 | 6.9 | 1 | 271 |
| IMGA\|Medtr2g094180.1 | Protein disulfide isomerase L-2 | 35.3 (M:35.3) | 2 | 63 | 4.5 | 1 | 272 |
| DYHG_CHLRE | Dynein gamma chain | 35.2 (M:35.2) | 0.6 | 512.5 | 6.2 | 1 | 273 |
| IMGA\|Medtr4g119230.2 | Serine/threonine-protein phosphatase 2A regulatory subunit B | 35.1 (M:35.1) | 3.6 | 42.2 | 4.6 | 1 | 274 |
| ATPB_SCHSP | ATP synthase subunit beta (EC 3.6.3.14) | 35.1 (M:35.1) | 4.6 | 53.6 | 5.1 | 1 | 275 |

**M. truncatula 14 d**

| Accession | Protein | Scores | S.Coverage | MW [kDa] | pI | Peptides | Rank |
|---|---|---|---|---|---|---|---|
| IMGA\|Medtr7g079820.1 | Convicilin (Fragment) | 1420.5 (M:1420.5) | 45.6 | 53.7 | 5.6 | 23 | 1 |
| IMGA\|Medtr7g079780.1 | Convicilin (Fragment) | 1408.7 (M:1408.7) | 44.2 | 53.2 | 5.5 | 8 | 2 |
| IMGA\|Medtr7g079740.1 | Convicilin (Fragment) | 1363.3 (M:1363.3) | 40.8 | 54.2 | 5.6 | 2 | 3 |
| IMGA\|Medtr7g079730.1 | Convicilin (Fragment) | 1172.4 (M:1172.4) | 36.9 | 53.1 | 5.3 | 1 | 4 |
| IMGA\|Medtr1g072600.1 | Legumin B (Fragment) | 1042.2 (M:1042.2) | 35.3 | 67.2 | 6.5 | 15 | 5 |
| IMGA\|Medtr7g079770.1 | Provicilin (Fragment) | 1008.5 (M:1008.5) | 37.4 | 53.1 | 5.3 | 2 | 6 |
| IMGA\|Medtr1g072610.2 | Legumin B (Fragment) | 867.3 (M:867.3) | 36.8 | 63.4 | 6.4 | 2 | 7 |
| IMGA\|Medtr1g072630.1 | Legumin B | 827.9 (M:827.9) | 30.4 | 65.3 | 6.1 | 2 | 8 |
| IMGA\|Medtr1g108770.1 | ATP synthase subunit beta | 491.8 (M:491.8) | 13.4 | 120.9 | 5.8 | 10 | 9 |
| IMGA\|Medtr4g103920.1 | Glyceraldehyde-3-phosphate dehydrogenase (Fragment) | 336.2 (M:336.2) | 31.0 | 36.6 | 6.7 | 6 | 10 |
| IMGA\|Medtr7g024390.1 | Heat shock protein 70 | 278.2 (M:278.2) | 12.3 | 70.9 | 4.9 | 5 | 11 |
| IMGA\|Medtr1g083960.1 | Calreticulin | 244.7 (M:244.7) | 17.5 | 48.4 | 4.3 | 4 | 12 |
| VCLC_PEA | Vicilin precursor | 230.6 (M:230.6) | 8.9 | 52.2 | 5.3 | 1 | 13 |
| EF1A_MAIZE | Elongation factor 1-alpha (EF-1-alpha) | 219.3 (M:219.3) | 19.9 | 49.2 | 9.8 | 5 | 14 |
| TBA_PRUDU | Tubulin alpha chain | 214.2 (M:214.2) | 15.8 | 49.5 | 4.8 | 4 | 15 |
| IMGA\|Medtr3g098420.1 | Staphylococcal nuclease domain-containing protein 1 | 201.6 (M:201.6) | 6.2 | 108.2 | 6.6 | 3 | 16 |
| BIP4_TOBAC | Luminal-binding protein 4 precursor (BiP 4) | 194.5 (M:194.5) | 7.3 | 73.5 | 4.9 | 2 | 17 |
| IMGA\|Medtr8g106790.1 | Guanine nucleotide-binding protein subunit beta-like protein | 191.4 (M:191.4) | 19.4 | 35.7 | 7.8 | 3 | 18 |
| ALF2_PEA | Fructose-bisphosphate aldolase, cytoplasmic isozyme 2 | 191.3 (M:191.3) | 12.5 | 38.5 | 6.9 | 4 | 19 |
| TBA1_ELEIN | Tubulin alpha-1 chain (Alpha-1 tubulin) | 189.4 (M:189.4) | 15.7 | 49.7 | 4.8 | 1 | 20 |
| IMGA\|Medtr6g055020.1 | ATP synthase subunit beta, chloroplastic | 182.5 (M:182.5) | 20.3 | 30.1 | 6.2 | 2 | 21 |
| IMGA\|Medtr4g019110.1 | Tubulin beta chain | 180.4 (M:180.4) | 10.7 | 50.5 | 4.6 | 3 | 22 |
| IMGA\|Medtr6g021800.1 | Elongation factor 1-alpha | 178.2 (M:178.2) | 8.6 | 109.7 | 9.8 | 1 | 23 |
| TBB1_LUPAL | Tubulin beta-1 chain (Beta-1 tubulin) | 177.1 (M:177.1) | 10.7 | 50.1 | 4.6 | 1 | 24 |
| IMGA\|Medtr2g099560.1 | Lipoxygenase | 176.8 (M:176.8) | 4.3 | 96.9 | 5.8 | 2 | 25 |
| PDI_MEDSA | Protein disulfide-isomerase precursor (EC 5.3.4.1) | 174.2 (M:174.2) | 12.5 | 57.1 | 4.8 | 4 | 26 |
| IMGA\|Medtr4g076100.1 | Aminotransferase | 166.6 (M:166.6) | 8.6 | 56.5 | 8.5 | 3 | 27 |
| VCL_VICFA | Vicilin precursor | 163.9 (M:163.9) | 6.9 | 52.7 | 5.7 | 1 | 28 |
| IMGA\|Medtr8g085980.1 | Alpha-tubulin | 162.0 (M:162.0) | 13.8 | 49.5 | 4.9 | 1 | 29 |
| IMGA\|Medtr2g066120.1 | Phosphoglycerate kinase | 157.7 (M:157.7) | 18.0 | 42.4 | 5.7 | 4 | 30 |
| PDIA6_MEDSA | Probable protein disulfide-isomerase A6 precursor (EC 5.3.4.1) (P5) | 151.6 (M:151.6) | 9.6 | 40.5 | 5.3 | 3 | 31 |
| IMGA\|Medtr4g075290.1 | Peptidyl-prolyl cis-trans isomerase | 149.2 (M:149.2) | 39.5 | 18.2 | 9.5 | 3 | 32 |
| IMGA\|Medtr3g100500.1 | Aspartic proteinase nepenthesin-1 | 140.0 (M:140.0) | 8.0 | 46.7 | 9.6 | 2 | 33 |
| ENO2_HEVBR | Enolase 2 (EC 4.2.1.11) | 139.0 (M:139.0) | 10.3 | 47.9 | 5.9 | 2 | 34 |
| IMGA\|Medtr3g108280.1 | 60S acidic ribosomal protein p0 | 137.1 (M:137.1) | 10.8 | 34.4 | 5.1 | 2 | 35 |
| ENO_RICCO | Enolase (EC 4.2.1.11) | 133.2 (M:133.2) | 13.9 | 47.9 | 5.5 | 2 | 36 |
| RUBB_SECCE | RuBisCO large subunit-binding protein subunit beta | 130.2 (M:130.2) | 8.4 | 53.4 | 4.7 | 2 | 37 |
| IMGA\|Medtr3g114420.1 | Subtilisin-type protease | 128.7 (M:128.7) | 8.6 | 82.4 | 8.4 | 3 | 38 |
| IMGA\|Medtr4g124660.2 | Sucrose synthase | 126.8 (M:126.8) | 7.0 | 92.2 | 5.8 | 3 | 39 |
| IMGA\|Medtr5g080450.1 | Ribulose-1 5-bisphosphate carboxylase/oxygenase activase 1 | 126.7 (M:126.7) | 7.4 | 52.3 | 6.1 | 2 | 40 |
| IMGA\|Medtr8g012330.3 | Ribosomal protein S8 | 125.5 (M:125.5) | 21.7 | 16.0 | 11.6 | 2 | 41 |
| RBL_LUPDE | Ribulose bisphosphate carboxylase large chain (EC 4.1.1.39) | 125.4 (M:125.4) | 4.8 | 50.2 | 6.3 | 2 | 42 |
| EF1A_MANES | Elongation factor 1-alpha (EF-1-alpha) | 124.8 (M:124.8) | 14.5 | 49.3 | 9.8 | 1 | 43 |
| BIP2_MAIZE | Luminal-binding protein 2 precursor (BiP2) | 120.9 (M:120.9) | 4.5 | 73.0 | 5.0 | 1 | 44 |
| IMGA\|Medtr7g086300.3 | Methionine synthase (Fragment) | 119.5 (M:119.5) | 10.2 | 71.0 | 9.1 | 3 | 45 |
| IMGA\|Medtr1g023120.1 | Beta-galactosidase | 117.8 (M:117.8) | 6.3 | 83.1 | 9.2 | 2 | 46 |
| IMGA\|Medtr2g033980.1 | Unknown Protein | 113.4 (M:113.4) | 11.6 | 28.7 | 10.1 | 1 | 47 |
| IF413_TOBAC | Eukaryotic initiation factor 4A-13 | 113.1 (M:113.1) | 8.2 | 40.2 | 5.0 | 2 | 48 |
| IMGA\|Medtr8g039540.1 | Aspartic proteinase nepenthesin-1 | 109.9 (M:109.9) | 6.9 | 46.8 | 10.5 | 2 | 49 |
| IMGA\|Medtr3g093110.1 | Ribosomal protein L9 (Fragment) | 106.6 (M:106.6) | 15.0 | 21.7 | 9.9 | 2 | 50 |
| IMGA\|Medtr1g043040.1 | Malate dehydrogenase | 105.4 (M:105.4) | 7.5 | 35.5 | 6.1 | 2 | 51 |
| IMGA\|Medtr6g009650.1 | Kunitz-type trypsin inhibitor-like 1 protein | 102.7 (M:102.7) | 26.7 | 22.5 | 4.8 | 2 | 52 |
| UBIQ_AVESA | Ubiquitin | 100.8 (M:100.8) | 32.9 | 8.5 | 7.6 | 2 | 53 |
| IMGA\|Medtr4g059720.1 | Fasciclin-like arabinogalactan protein 12 | 98.7 (M:98.7) | 13.2 | 26.9 | 9.9 | 2 | 54 |
| ILV5_ORYSJ | Ketol-acid reductoisomerase | 98.4 (M:98.4) | 6.7 | 62.3 | 6.0 | 2 | 55 |
| PSMD3_TOBAC | Probable 26S proteasome non-ATPase regulatory subunit 3 | 98.0 (M:98.0) | 7.0 | 55.5 | 9.5 | 2 | 56 |
| PROF_PRUPE | Profilin | 96.6 (M:96.6) | 17.6 | 14.0 | 4.6 | 2 | 57 |
| PSBB_DAUCA | Photosystem II CP47 | 95.9 (M:95.9) | 6.9 | 55.9 | 6.4 | 2 | 58 |
| IMGA\|Medtr1g025430.1 | Endoplasmin homolog | 95.2 (M:95.2) | 2.9 | 94.1 | 4.6 | 2 | 59 |
| IMGA\|Medtr2g095750.1 | Serine carboxypeptidase | 93.5 (M:93.5) | 7.6 | 57.3 | 5.2 | 2 | 60 |
| IMGA\|Medtr1g079530.1 | GDSL esterase/lipase | 93.1 (M:93.1) | 7.5 | 39.6 | 9.4 | 2 | 61 |
| HSP7S_PEA | Stromal 70 kDa heat shock-related protein | 92.8 (M:92.8) | 4.1 | 75.5 | 5.1 | 2 | 62 |
| IMGA\|Medtr5g062430.1 | Xylan 1 4-beta-xylosidase | 89.9 (M:89.9) | 4.5 | 87.9 | 9.3 | 2 | 63 |
| IMGA\|Medtr5g088660.1 | Elongation factor 1-beta 1 | 88.9 (M:88.9) | 17.0 | 24.2 | 4.5 | 2 | 64 |
| 14332_PSEMZ | 14-3-3-like protein 2 (Fragments) | 87.4 (M:87.4) | 28.8 | 7.7 | 4.1 | 1 | 65 |
| IMGA\|Medtr3g018780.1 | Annexin-like protein | 86.6 (M:86.6) | 4.1 | 36.2 | 8.7 | 1 | 66 |
| IMGA\|Medtr2g069050.1 | Elongation factor EF-2 (Fragment) | 86.6 (M:86.6) | 3.3 | 94.1 | 5.7 | 2 | 67 |
| IMGA\|Medtr2g083110.1 | Conglutin | 85.1 (M:85.1) | 12.1 | 16.7 | 7.8 | 2 | 68 |
| IMGA\|Medtr4g114770.1 | Methyl binding domain protein (Fragment) | 82.0 (M:82.0) | 13.5 | 38.9 | 4.5 | 2 | 69 |
| IMGA\|Medtr8g070780.1 | Defensin | 81.1 (M:81.1) | 31.2 | 8.3 | 10.8 | 1 | 70 |
| IMGA\|Medtr5g027670.1 | 40S ribosomal protein S18 | 80.1 (M:80.1) | 24.8 | 13.6 | 11.1 | 2 | 71 |
| PSBQ_ONOVI | Oxygen-evolving enhancer protein 3 | 77.4 (M:77.4) | 5.6 | 24.8 | 10.0 | 1 | 72 |
| IMGA\|Medtr3g117000.1 | Unknown Protein | 77.0 (M:77.0) | 17.7 | 13.4 | 5.5 | 1 | 73 |
| IMGA\|Medtr5g030940.1 | Oligopeptidase A | 75.7 (M:75.7) | 2.1 | 109.9 | 6.0 | 1 | 74 |
| ATPB_DICAN | ATP synthase subunit beta (EC 3.6.3.14) | 73.3 (M:73.3) | 2.3 | 50.9 | 4.8 | 1 | 75 |
| LEG_CICAR | Legumin OS | 72.4 (M:72.4) | 2.2 | 56.2 | 6.2 | 1 | 76 |
| IMGA\|Medtr2g029730.1 | Peroxidase | 72.2 (M:72.2) | 4.3 | 37.4 | 6.2 | 1 | 77 |
| H2B2_SOLLC | Histone H2B.2 | 72.1 (M:72.1) | 10.7 | 15.4 | 10.6 | 1 | 78 |
| IMGA\|Medtr4g112670.1 | Strictosidine synthase 1 | 71.7 (M:71.7) | 5.1 | 35.5 | 6.2 | 1 | 79 |
| IMGA\|AC146630_2.1 | 2-cys peroxiredoxin BAS1 | 68.1 (M:68.1) | 9.4 | 29.0 | 6.1 | 1 | 80 |
| ICDHC_ARATH | Cytosolic isocitrate dehydrogenase | 65.8 (M:65.8) | 4.1 | 45.7 | 6.1 | 1 | 81 |

| | | | | | | | |
|---|---|---|---|---|---|---|---|
| IMGA\|Medtr3g087170.1 | Ras-related protein Rab-6A | 64.6 (M:64.6) | 10.9 | 11.7 | 9.4 | 1 | 82 |
| IMGA\|Medtr6g023590.1 | Short-chain alcohol dehydrogenase | 64.6 (M:64.6) | 6.4 | 28.1 | 5.7 | 1 | 83 |
| IMGA\|Medtr3g092600.1 | 60S acidic ribosomal protein P1 | 63.6 (M:63.6) | 14.5 | 11.1 | 4.1 | 1 | 84 |
| IMGA\|Medtr2g103730.1 | Phospholipase D alpha 1 | 62.9 (M:62.9) | 3.0 | 92.0 | 5.4 | 1 | 85 |
| IMGA\|Medtr5g091120.2 | 60S ribosomal protein L18a | 62.2 (M:62.2) | 9.5 | 17.5 | 11.0 | 1 | 86 |
| IMGA\|Medtr7g088680.1 | Nascent polypeptide-associated complex subunit alpha-like protein | 61.9 (M:61.9) | 8.4 | 22.1 | 4.2 | 1 | 87 |
| RL24_HORVU | 60S ribosomal protein L24 | 61.9 (M:61.9) | 7.4 | 18.4 | 11.4 | 1 | 88 |
| IMGA\|Medtr5g033090.1 | 60S ribosomal protein L27a-3 | 61.5 (M:61.5) | 7.9 | 18.5 | 11.0 | 1 | 89 |
| IMGA\|Medtr2g098010.1 | Proteasome subunit beta type | 61.0 (M:61.0) | 11.3 | 25.4 | 6.3 | 1 | 90 |
| HS903_ARATH | Heat shock protein 90-3 | 59.9 (M:59.9) | 2.0 | 80.0 | 4.8 | 1 | 91 |
| IMGA\|Medtr2g089860.1 | Subtilisin-like protease | 59.8 (M:59.8) | 2.0 | 81.8 | 8.7 | 1 | 92 |
| IMGA\|Medtr4g103800.1 | Adenylate kinase B | 59.5 (M:59.5) | 6.2 | 26.7 | 8.8 | 1 | 93 |
| ALFC2_PEA | Fructose-bisphosphate aldolase 2 | 58.6 (M:58.6) | 8.0 | 37.8 | 5.4 | 1 | 94 |
| NDK1_PSEMZ | Nucleoside diphosphate kinase 1 (Fragments) | 58.1 (M:58.1) | 50.0 | 1.9 | 4.4 | 1 | 95 |
| IMGA\|Medtr7g021850.2 | Aldo/keto reductase | 56.5 (M:56.5) | 5.9 | 24.5 | 6.8 | 1 | 96 |
| IMGA\|Medtr7g077880.1 | Unknown Protein | 56.4 (M:56.4) | 4.5 | 30.4 | 4.9 | 1 | 97 |
| MSBP1_ARATH | Membrane steroid-binding protein 1 | 55.9 (M:55.9) | 6.8 | 24.4 | 4.5 | 1 | 98 |
| IMGA\|Medtr2g028190.1 | Aspartic proteinase nepenthesin-2 | 54.8 (M:54.8) | 4.6 | 48.6 | 9.7 | 1 | 99 |
| IMGA\|Medtr2g005570.2 | Elongation factor 1-gamma | 54.5 (M:54.5) | 4.5 | 47.7 | 7.7 | 1 | 100 |
| IMGA\|Medtr7g052690.1 | Early tobacco anther 1 | 53.6 (M:53.6) | 13.9 | 16.9 | 4.5 | 1 | 101 |
| IMGA\|Medtr1g064060.2 | Adenosine kinase 2 | 53.5 (M:53.5) | 3.5 | 35.1 | 5.6 | 1 | 102 |
| IMGA\|Medtr8g086470.1 | Cysteine proteinase 2 (Fragment) | 53.4 (M:53.4) | 6.0 | 40.4 | 5.0 | 1 | 103 |
| IMGA\|Medtr8g089110.1 | Basic blue protein | 53.3 (M:53.3) | 17.4 | 13.0 | 10.6 | 1 | 104 |
| IMGA\|Medtr7g118060.1 | 60s acidic ribosomal protein P1 | 53.1 (M:53.1) | 32.7 | 11.4 | 4.0 | 1 | 105 |
| IMGA\|Medtr3g115930.1 | Unknown Protein | 51.5 (M:51.5) | 9.5 | 17.9 | 10.2 | 1 | 106 |
| APX_PSEMZ | L-ascorbate peroxidase, cytosolic (Fragment) | 51.3 (M:51.3) | 100.0 | 1.1 | 4.1 | 1 | 107 |
| IMGA\|Medtr3g092090.1 | YSL transporter 3 | 51.2 (M:51.2) | 2.9 | 92.9 | 9.0 | 1 | 108 |
| IMGA\|Medtr2g102180.1 | 97 kDa heat shock protein | 50.6 (M:50.6) | 1.5 | 95.2 | 5.0 | 1 | 109 |
| IMGA\|Medtr8g105340.1 | 40S ribosomal protein S2 | 50.5 (M:50.5) | 5.7 | 30.4 | 11.0 | 1 | 110 |
| IMGA\|Medtr1g045410.1 | 60S ribosomal protein L4 | 50.4 (M:50.4) | 3.0 | 44.7 | 10.9 | 1 | 111 |
| IMGA\|Medtr1g094630.1 | 60S ribosomal protein L4 | 49.6 (M:49.6) | 1.7 | 130.9 | 6.5 | 1 | 112 |
| IMGA\|Medtr5g019780.1 | Vicilin-like antimicrobial peptides 2-3 (Fragment) | 49.3 (M:49.3) | 2.0 | 86.4 | 5.2 | 1 | 113 |
| IMGA\|Medtr3g087590.3 | L-myo inositol-1 phosphate synthase 1 | 49.0 (M:49.0) | 4.4 | 47.3 | 5.3 | 1 | 114 |
| IMGA\|Medtr3g088160.3 | Ascorbate peroxidase | 48.0 (M:48.0) | 6.5 | 42.1 | 9.3 | 1 | 115 |
| IMGA\|Medtr7g083790.1 | Phosphate carrier protein, mitochondrial | 47.4 (M:47.4) | 3.5 | 39.5 | 10.0 | 1 | 116 |
| IMGA\|Medtr8g072010.1 | Carboxylic ester hydrolase | 47.0 (M:47.0) | 4.4 | 45.6 | 4.9 | 1 | 117 |
| IMGA\|AC233663_14.1 | RuBisCO large subunit-binding protein subunit alpha | 46.7 (M:46.7) | 2.8 | 49.3 | 4.8 | 1 | 118 |
| HIBC8_ARATH | 3-hydroxyisobutyryl-CoA hydrolase-like protein 5 | 46.6 (M:46.6) | 2.3 | 43.2 | 5.3 | 1 | 119 |
| AATM_LUPAN | Aspartate aminotransferase-P2 | 46.5 (M:46.5) | 3.3 | 49.9 | 8.6 | 1 | 120 |
| RGP1_ARATH | UDP-arabinopyranose mutase 1 | 46.3 (M:46.3) | 5.6 | 40.6 | 5.5 | 1 | 121 |
| IMGA\|Medtr7g074570.2 | 2,3-bisphosphoglycerate-independent phosphoglycerate mutase | 46.0 (M:46.0) | 5.1 | 55.3 | 5.3 | 1 | 122 |
| IMGA\|Medtr8g081490.1 | Adenosylhomocysteinase | 46.0 (M:46.0) | 2.9 | 49.8 | 5.2 | 1 | 123 |
| IMGA\|Medtr2g064660.2 | Photosystem II 10 kDa polypeptide | 45.8 (M:45.8) | 11.8 | 13.6 | 10.2 | 1 | 124 |
| IMGA\|Medtr4g130860.1 | Leucine aminopeptidase 2 | 44.8 (M:44.8) | 3.0 | 59.6 | 8.6 | 1 | 125 |
| IMGA\|Medtr2g025640.1 | Fumarylacetoacetase | 44.6 (M:44.6) | 3.6 | 46.0 | 5.7 | 1 | 126 |
| IMGA\|Medtr7g009330.1 | Outer plastidial membrane protein porin | 44.3 (M:44.3) | 6.9 | 29.6 | 9.4 | 1 | 127 |
| IMGA\|Medtr1g094980.2 | Phosphoglucosamine mutase | 44.0 (M:44.0) | 5.7 | 58.0 | 5.5 | 1 | 128 |
| IMGA\|Medtr5g077000.2 | UTP-glucose 1 phosphate uridylyltransferase | 43.9 (M:43.9) | 3.8 | 51.4 | 5.1 | 1 | 129 |
| SAR1B_BRACM | GTP-binding protein | 43.4 (M:43.4) | 6.7 | 22.1 | 6.6 | 1 | 130 |
| IMGA\|Medtr3g088450.1 | Photosystem Q(B) protein | 43.4 (M:43.4) | 9.6 | 12.8 | 10.5 | 1 | 131 |
| IMGA\|Medtr5g011250.1 | Leucoanthocyanidin dioxygenase (Fragment) | 43.3 (M:43.3) | 3.9 | 40.4 | 5.9 | 1 | 132 |
| IMGA\|Medtr3g092900.1 | Caffeic acid O-methyltransferase (Fragment) | 43.2 (M:43.2) | 7.9 | 39.9 | 5.6 | 1 | 133 |
| IMGA\|Medtr1g006010.1 | ATP synthase subunit alpha | 43.1 (M:43.1) | 1.3 | 122.7 | 9.9 | 1 | 134 |
| NRL1_ARATH | Nitrilase 1 | 42.8 (M:42.8) | 2.0 | 38.1 | 5.8 | 1 | 135 |
| RAN_POPEU | GTP-binding nuclear protein Ran (Fragments) | 42.3 (M:42.3) | 50.0 | 3.3 | 9.5 | 1 | 136 |
| THIC_ARATH | Phosphomethylpyrimidine synthase | 42.3 (M:42.3) | 2.6 | 71.9 | 6.0 | 1 | 137 |
| IMGA\|Medtr1g031460.1 | Kinase R-like protein (Fragment) | 42.0 (M:42.0) | 1.2 | 73.4 | 7.0 | 1 | 138 |
| IMGA\|Medtr4g075340.1 | Translocon-associated protein subunit alpha | 41.8 (M:41.8) | 8.6 | 27.5 | 4.6 | 1 | 139 |
| IMGA\|Medtr6g071090.1 | Translationally-controlled tumor protein homolog | 41.7 (M:41.7) | 5.6 | 18.2 | 4.6 | 1 | 140 |
| IMGA\|Medtr4g083230.1 | Transcription factor Pur-alpha 1 | 41.7 (M:41.7) | 9.6 | 32.1 | 5.6 | 1 | 141 |
| EF1B_ORYSJ | Elongation factor 1-beta | 41.6 (M:41.6) | 7.6 | 23.8 | 4.5 | 1 | 142 |
| NU5C_VICFA | NAD(P)H-quinone oxidoreductase subunit 5 | 41.5 (M:41.5) | 2.9 | 84.7 | 9.6 | 1 | 143 |
| IMGA\|Medtr4g028370.1 | Non-specific lipid-transfer protein | 41.2 (M:41.2) | 3.7 | 58.4 | 10.8 | 1 | 144 |
| IMGA\|Medtr7g060560.1 | 60 kDa chaperonin (Fragment) | 41.1 (M:41.1) | 2.6 | 48.4 | 8.9 | 1 | 145 |
| IMGA\|Medtr3g098430.1 | Calnexin homolog | 40.9 (M:40.9) | 3.1 | 61.7 | 4.6 | 1 | 146 |
| PSBP1_ARATH | Oxygen-evolving enhancer protein 2-1 | 40.7 (M:40.7) | 5.3 | 28.1 | 7.7 | 1 | 147 |
| IMGA\|Medtr3g109190.1 | Oleosin | 40.5 (M:40.5) | 17.4 | 16.8 | 9.3 | 1 | 148 |
| IMGA\|Medtr8g106020.1 | 40S ribosomal protein S16 | 40.4 (M:40.4) | 7.1 | 16.1 | 11.0 | 1 | 149 |
| CY12_SOLTU | Cytochrome c1-2, heme protein, mitochondrial (Fragment) | 39.9 (M:39.9) | 9.2 | 28.6 | 5.2 | 1 | 150 |
| PAO_ARATH | Pheophorbide a oxygenase | 39.8 (M:39.8) | 4.1 | 60.7 | 7.9 | 1 | 151 |
| EFTU_PEA | Elongation factor Tu | 39.6 (M:39.6) | 3.3 | 53.0 | 6.7 | 1 | 152 |
| IMGA\|Medtr5g098770.1 | Chlorophyll a-b binding protein 8 | 39.5 (M:39.5) | 27.7 | 10.3 | 9.9 | 1 | 153 |
| CALM_MALDO | Calmodulin | 39.5 (M:39.5) | 11.4 | 16.8 | 4.0 | 1 | 154 |
| IMGA\|Medtr1g007370.1 | RNA polymerase sigma-B factor | 39.5 (M:39.5) | 3.1 | 62.7 | 9.9 | 1 | 155 |
| IMGA\|Medtr5g074860.2 | Peroxidase | 39.2 (M:39.2) | 3.1 | 31.4 | 10.4 | 1 | 156 |
| IMGA\|AC235678_12.1 | Fructose-bisphosphate aldolase | 38.9 (M:38.9) | 8.7 | 16.1 | 10.2 | 1 | 157 |
| RS271_ARATH | 40S ribosomal protein S27-1 | 38.8 (M:38.8) | 15.5 | 9.4 | 10.6 | 1 | 158 |
| IMGA\|Medtr3g021080.1 | Unknown Protein | 38.5 (M:38.5) | 10.5 | 20.3 | 4.9 | 1 | 159 |
| ACCC_POPEU | Biotin carboxylase (Fragments) | 38.4 (M:38.4) | 37.5 | 4.3 | 7.3 | 1 | 160 |
| IMGA\|AC157372_6.1 | Oxygen-evolving enhancer protein 1 (Fragment) | 38.0 (M:38.0) | 6.4 | 35.0 | 6.3 | 1 | 161 |
| IMGA\|Medtr3g100220.1 | Argininosuccinate lyase | 37.8 (M:37.8) | 3.8 | 52.4 | 5.6 | 1 | 162 |
| PGL1B_ARATH | PGR5-like protein 1B | 37.5 (M:37.5) | 6.4 | 34.9 | 4.9 | 1 | 163 |
| IMGA\|Medtr2g019860.1 | Acyl-CoA-binding domain-containing protein 4 | 37.4 (M:37.4) | 2.7 | 84.1 | 6.9 | 1 | 164 |
| CADH7_ARATH | Cinnamyl alcohol dehydrogenase 7 | 37.4 (M:37.4) | 7.0 | 38.2 | 6.9 | 1 | 165 |

| | | | | | | | |
|---|---|---|---|---|---|---|---|
| REV1_ARATH | DNA repair protein | 37.1 (M:37.1) | 1.7 | 122.2 | 6.3 | 1 | 166 |
| IMGA\|Medtr7g083130.1 | Acyltransferase-like protein | 37.0 (M:37.0) | 2.4 | 75.0 | 7.8 | 1 | 167 |
| IMGA\|Medtr7g078470.1 | Unknown Protein | 36.9 (M:36.9) | 31.4 | 7.6 | 11.4 | 1 | 168 |
| IMGA\|Medtr6g052140.1 | 40S ribosomal protein S3 | 36.9 (M:36.9) | 6.0 | 26.2 | 10.4 | 1 | 169 |
| NU2C_ANACO | NAD(P)H-quinone oxidoreductase subunit 2 | 36.7 (M:36.7) | 6.9 | 56.8 | 5.3 | 1 | 170 |
| IMGA\|Medtr5g042000.1 | Cell division cycle and apoptosis regulator protein 1 | 36.7 (M:36.7) | 0.9 | 172.2 | 9.0 | 1 | 171 |
| IMGA\|Medtr8g046140.2 | 60S ribosomal protein L11 | 36.6 (M:36.6) | 11.1 | 13.2 | 10.4 | 1 | 172 |
| IMGA\|Medtr5g062540.1 | Beta-glucosidase (Fragment) | 36.5 (M:36.5) | 4.9 | 39.5 | 6.0 | 1 | 173 |
| IMGA\|Medtr7g083560.1 | Translocon-associated protein subunit beta | 36.5 (M:36.5) | 8.2 | 21.0 | 9.9 | 1 | 174 |
| IMGA\|Medtr3g057190.1 | (+)-neomenthol dehydrogenase | 36.2 (M:36.2) | 3.7 | 31.9 | 5.4 | 1 | 175 |
| IMGA\|Medtr3g052490.1 | GDSL esterase/lipase | 36.1 (M:36.1) | 6.3 | 41.0 | 9.4 | 1 | 176 |
| MDHM_FRAAN | Malate dehydrogenase | 36.0 (M:36.0) | 3.5 | 35.6 | 9.4 | 1 | 177 |
| IMGA\|Medtr7g108580.1 | Bifunctional protein folD | 35.9 (M:35.9) | 5.8 | 31.5 | 7.9 | 1 | 178 |
| IMGA\|Medtr8g022820.1 | Ethylene-responsive transcription factor 5 | 35.9 (M:35.9) | 5.5 | 42.4 | 4.6 | 1 | 179 |
| RPOC2_PSINU | DNA-directed RNA polymerase beta'' chain (EC 2.7.7.6) (PEP) | 35.8 (M:35.8) | 0.8 | 161.7 | 10.3 | 1 | 180 |
| IMGA\|Medtr5g084140.1 | Nodule-specific glycine-rich protein 1E | 35.8 (M:35.8) | 9.1 | 41.0 | 10.0 | 1 | 181 |
| G3PA_TOBAC | Glyceraldehyde-3-phosphate dehydrogenase A | 35.5 (M:35.5) | 4.1 | 41.8 | 6.7 | 1 | 182 |
| CCX2_ARATH | Cation/calcium exchanger 2 | 35.5 (M:35.5) | 5.2 | 61.1 | 6.4 | 1 | 183 |
| IMGA\|Medtr8g106860.1 | Unknown Protein | 35.4 (M:35.4) | 7.4 | 13.0 | 7.8 | 1 | 184 |

**M. orbicularis 14 d**

| Accession | Protein | Scores | S.Coverage | MW [kDa] | pI | Peptides | Rank |
|---|---|---|---|---|---|---|---|
| IMGA\|Medtr4g103920.1 | Glyceraldehyde-3-phosphate dehydrogenase (Fragment) | 719.8 (M:719.8) | 45.7 | 36.6 | 6.7 | 10 | 1 |
| IMGA\|Medtr1g108770.1 | ATP synthase subunit beta plex, beta subunit | 705.2 (M:705.2) | 16.5 | 120.9 | 5.8 | 12 | 2 |
| IMGA\|Medtr1g025430.1 | Endoplasmin homolog | 608.0 (M:608.0) | 17.8 | 94.1 | 4.6 | 12 | 3 |
| IMGA\|Medtr3g085850.1 | Glyceraldehyde 3-phosphate dehydrogenase | 569.2 (M:569.2) | 33.2 | 37 | 7.7 | 3 | 4 |
| IMGA\|Medtr3g018780.1 | Annexin-like protein RJ4 | 495.7 (M:495.7) | 26.1 | 36.2 | 8.7 | 10 | 5 |
| IMGA\|Medtr8g038210.1 | Annexin-like protein RJ4 | 476.8 (M:476.8) | 19.8 | 38.6 | 7.1 | 8 | 6 |
| BIP_SOLLC | Luminal-binding protein precursor (BiP) | 436.8 (M:436.8) | 15.6 | 73.2 | 5 | 7 | 7 |
| IMGA\|Medtr7g079780.1 | Convicilin (Fragment) | 435.9 (M:435.9) | 20.5 | 53.2 | 5.5 | 7 | 8 |
| IMGA\|Medtr7g079740.1 | Convicilin (Fragment) | 431.1 (M:431.1) | 20.1 | 54.2 | 5.6 | 1 | 9 |
| IMGA\|Medtr2g066120.1 | Phosphoglycerate kinase | 413.9 (M:413.9) | 30.9 | 42.4 | 5.7 | 7 | 10 |
| IMGA\|Medtr7g079820.1 | Convicilin (Fragment) | 398.7 (M:398.7) | 16.3 | 53.7 | 5.6 | 1 | 11 |
| EF1A_MAIZE | Elongation factor 1-alpha | 385.5 (M:385.5) | 19.5 | 49.2 | 9.8 | 7 | 12 |
| IMGA\|Medtr7g079770.1 | Provicilin (Fragment) | 382.3 (M:382.3) | 17.1 | 53.1 | 5.3 | 1 | 13 |
| HSP7C_ARATH | Heat shock 70 kDa protein 3 | 367.3 (M:367.3) | 16.6 | 71.1 | 4.8 | 5 | 14 |
| PDIA6_MEDSA | Probable protein disulfide-isomerase A6 precursor (EC 5.3.4.1) | 347.5 (M:347.5) | 21.4 | 40.5 | 5.3 | 6 | 15 |
| IMGA\|Medtr5g096430.1 | Heat shock protein 90 (Fragment) | 344.6 (M:344.6) | 15.6 | 80.1 | 4.8 | 6 | 16 |
| IMGA\|Medtr7g086300.2 | Methionine synthase | 328.1 (M:328.1) | 14.9 | 83.1 | 5.8 | 7 | 17 |
| IMGA\|Medtr1g087520.1 | Protein disulfide-isomerase | 320.7 (M:320.7) | 22.8 | 41.3 | 5.4 | 1 | 18 |
| IMGA\|Medtr1g045410.1 | 60S ribosomal protein L4 | 312.4 (M:312.4) | 20.7 | 44.7 | 10.9 | 5 | 19 |
| IMGA\|Medtr8g038220.1 | Annexin A3 | 312.2 (M:312.2) | 13.7 | 42.3 | 9.3 | 1 | 20 |
| IMGA\|Medtr1g006010.1 | ATP synthase subunit alpha (Fragment) | 310.1 (M:310.1) | 6.1 | 122.7 | 9.9 | 5 | 21 |
| IMGA\|Medtr4g061140.1 | Cytosolic ascorbate peroxidase | 278.2 (M:278.2) | 31.2 | 27.1 | 5.5 | 5 | 22 |
| IMGA\|Medtr1g072630.3 | Legumin B | 277.5 (M:277.5) | 19.3 | 52.2 | 5.8 | 5 | 23 |
| IMGA\|Medtr1g083960.1 | Calreticulin | 273.9 (M:273.9) | 18 | 48.4 | 4.3 | 4 | 24 |
| IMGA\|Medtr1g072630.2 | Legumin B (Fragment) | 272.8 (M:272.8) | 16.2 | 63.5 | 6.4 | 1 | 25 |
| IMGA\|Medtr1g043040.1 | Malate dehydrogenase | 272.5 (M:272.5) | 19.6 | 35.5 | 6.1 | 4 | 26 |
| IMGA\|Medtr4g070080.1 | Glycine-rich RNA binding protein 1 | 263.4 (M:263.4) | 24.8 | 15.7 | 5.4 | 4 | 27 |
| BIP2_MAIZE | Luminal-binding protein 2 precursor (BiP2) | 255.9 (M:255.9) | 9 | 73 | 5 | 1 | 28 |
| PDI_MEDSA | Protein disulfide-isomerase precursor (EC 5.3.4.1) | 247.0 (M:247.0) | 11.9 | 57.1 | 4.8 | 4 | 29 |
| IMGA\|Medtr5g064580.1 | 14-3-3 protein (Fragment) | 244.8 (M:244.8) | 27.7 | 29.2 | 4.5 | 4 | 30 |
| ENO_RICCO | Enolase (EC 4.2.1.11) | 243.6 (M:243.6) | 13.9 | 47.9 | 5.5 | 3 | 31 |
| PGKH2_ARATH | Phosphoglycerate kinase 2 | 238.8 (M:238.8) | 13.8 | 49.9 | 9.1 | 1 | 32 |
| CALR_NICPL | Calreticulin | 237.6 (M:237.6) | 13.5 | 47.5 | 4.3 | 2 | 33 |
| IMGA\|Medtr8g012330.2 | Ribosomal protein S8 | 235.4 (M:235.4) | 24.7 | 24.6 | 10.9 | 4 | 34 |
| IMGA\|Medtr4g063240.1 | Histone H2B | 234.9 (M:234.9) | 16.3 | 16.1 | 10.5 | 4 | 35 |
| IMGA\|Medtr8g067080.1 | ADP-ribosylation factor GTPase-activating protein AGD10 | 226.2 (M:226.2) | 9.8 | 43.9 | 9.7 | 3 | 36 |
| IMGA\|Medtr8g106790.1 | Guanine nucleotide-binding protein subunit beta-like | 221.0 (M:221.0) | 24.3 | 35.7 | 7.8 | 4 | 37 |
| PSBP_PEA | Oxygen-evolving enhancer protein 2 | 220.6 (M:220.6) | 22.4 | 28 | 9.1 | 4 | 38 |
| IMGA\|Medtr1g023120.1 | Beta-galactosidase | 216.0 (M:216.0) | 10.5 | 83.1 | 9.2 | 4 | 39 |
| IMGA\|Medtr7g088680.1 | Nascent polypeptide-associated complex subunit alpha-like protein | 203.5 (M:203.5) | 23.6 | 22.1 | 4.2 | 3 | 40 |
| CALM_MALDO | Calmodulin | 202.5 (M:202.5) | 33.6 | 16.8 | 4 | 3 | 41 |
| IMGA\|Medtr5g025120.1 | 60S ribosomal protein L4-1 | 201.1 (M:201.1) | 12.5 | 44.9 | 10.9 | 1 | 42 |
| IMGA\|Medtr2g089860.1 | Subtilisin-like protease | 198.2 (M:198.2) | 8.2 | 81.8 | 8.7 | 4 | 43 |
| IMGA\|Medtr1g098170.1 | 40S ribosomal protein S18 | 192.9 (M:192.9) | 18.4 | 17.6 | 11.3 | 3 | 44 |
| IMGA\|Medtr2g014030.1 | 40S ribosomal protein S6 | 190.9 (M:190.9) | 19.4 | 28.2 | 11.5 | 3 | 45 |
| IMGA\|Medtr3g009050.1 | Receptor-like protein kinase 2 | 187.8 (M:187.8) | 9.6 | 52.9 | 9.6 | 4 | 46 |
| IMGA\|Medtr5g062430.1 | Xylan 1 4-beta-xylosidase | 185.5 (M:185.5) | 5.5 | 87.9 | 9.3 | 4 | 47 |
| IMGA\|Medtr5g018940.1 | 40S ribosomal protein S4 | 184.1 (M:184.1) | 17.4 | 29.9 | 10.8 | 4 | 48 |
| H4_CAPAN | Histone H4 | 181.2 (M:181.2) | 35.9 | 11.4 | 12 | 4 | 49 |
| IMGA\|Medtr5g033090.1 | 60S ribosomal protein L27a-3 | 180.9 (M:180.9) | 20.1 | 18.5 | 11 | 3 | 50 |
| NDK1_PEA | Nucleoside diphosphate kinase 1 (EC 2.7.4.6) | 171.8 (M:171.8) | 22.8 | 16.5 | 5.9 | 3 | 51 |
| IMGA\|Medtr5g088660.1 | Elongation factor 1-beta 1 | 164.8 (M:164.8) | 24.2 | 24.2 | 4.5 | 3 | 52 |
| IMGA\|Medtr2g029730.1 | Peroxidase | 164.1 (M:164.1) | 4.3 | 37.4 | 6.2 | 2 | 53 |
| IMGA\|Medtr5g062650.1 | Unknown Protein | 162.3 (M:162.3) | 8.5 | 86.3 | 9.1 | 2 | 54 |
| IMGA\|Medtr4g075290.1 | Peptidyl-prolyl cis-trans isomerase | 162.1 (M:162.1) | 20.3 | 18.2 | 9.5 | 3 | 55 |
| IMGA\|Medtr4g059400.1 | 60S ribosomal protein L12 | 161.3 (M:161.3) | 22.9 | 17.8 | 9.7 | 3 | 56 |
| IMGA\|Medtr3g092980.2 | Nascent polypeptide-associated complex alpha subunit-like protein | 150.5 (M:150.5) | 14.2 | 23.6 | 4.2 | 3 | 57 |
| IMGA\|Medtr8g085980.1 | Alpha-tubulin | 145.3 (M:145.3) | 12.2 | 49.5 | 4.9 | 3 | 58 |
| IMGA\|Medtr2g038250.1 | 60S ribosomal protein L7-4 | 144.8 (M:144.8) | 11.5 | 28.5 | 10.4 | 2 | 59 |
| IMGA\|Medtr3g098430.1 | Calnexin homolog | 143.8 (M:143.8) | 7.4 | 61.7 | 4.6 | 3 | 60 |
| IMGA\|Medtr1g090130.1 | Chaperonin CPN60-2 | 142.7 (M:142.7) | 8.7 | 61.1 | 6.3 | 3 | 61 |
| IMGA\|Medtr7g084800.1 | Glyceraldehyde-3-phosphate dehydrogenase A | 141.4 (M:141.4) | 13.4 | 43 | 9.3 | 3 | 62 |
| IMGA\|Medtr2g066110.1 | Phosphoglycerate kinase (Fragment) | 138.9 (M:138.9) | 13.6 | 50 | 6.8 | 1 | 63 |
| IMGA\|Medtr2g008050.1 | Actin | 137.7 (M:137.7) | 14.1 | 41.6 | 5.2 | 3 | 64 |
| ALF_CICAR | Fructose-bisphosphate aldolase | 137.0 (M:137.0) | 7.2 | 38.4 | 6.2 | 2 | 65 |
| TBB3_SOYBN | Tubulin beta chain (Beta tubulin) (Fragment) | 133.8 (M:133.8) | 11.8 | 45.7 | 5.6 | 3 | 66 |
| IMGA\|Medtr6g005820.3 | ADP-ribosylation factor | 133.0 (M:133.0) | 22 | 18.7 | 5.5 | 3 | 67 |
| COMT1_MEDSA | Caffeic acid 3-O-methyltransferase | 129.1 (M:129.1) | 13.2 | 39.9 | 5.6 | 2 | 68 |
| IMGA\|Medtr3g092970.1 | Nascent polypeptide-associated complex subunit alpha | 128.4 (M:128.4) | 13.7 | 24.7 | 4.1 | 1 | 69 |
| IMGA\|Medtr8g012380.1 | FK506-binding protein 2 | 127.7 (M:127.7) | 9.9 | 16.5 | 9 | 1 | 70 |
| IMGA\|Medtr8g058480.1 | Zinc finger CCCH domain-containing protein 51 | 126.3 (M:126.3) | 7 | 39 | 4.7 | 2 | 71 |
| ATPB_VANDA | ATP synthase subunit beta chloroplastic (Fragment) | 123.4 (M:123.4) | 9.6 | 51.6 | 4.7 | 2 | 72 |
| ATPA_PEA | ATP synthase subunit alpha, chloroplastic | 122.3 (M:122.3) | 7.8 | 54.6 | 5.6 | 3 | 73 |
| IF413_TOBAC | Eukaryotic initiation factor 4A-13 | 121.1 (M:121.1) | 9.3 | 40.2 | 5 | 2 | 74 |
| IMGA\|Medtr1g083460.1 | Ribosomal protein L18 | 119.9 (M:119.9) | 15 | 20.8 | 12 | 2 | 75 |
| IMGA\|Medtr4g095360.1 | Subtilisin-like protease | 119.8 (M:119.8) | 4.8 | 79.8 | 6.4 | 2 | 76 |
| IMGA\|Medtr3g093110.1 | Ribosomal protein L9 (Fragment) | 119.0 (M:119.0) | 23.8 | 21.7 | 9.9 | 3 | 77 |
| IMGA\|Medtr7g118060.1 | 60s acidic ribosomal protein P1 | 116.4 (M:116.4) | 44.2 | 11.4 | 4 | 2 | 78 |
| ATPB_GOSHI | ATP synthase subunit beta (EC 3.6.3.14) | 116.4 (M:116.4) | 8 | 53.6 | 5.1 | 1 | 79 |
| IMGA\|Medtr1g106900.1 | 40S ribosomal protein S18 | 116.2 (M:116.2) | 18.4 | 17.6 | 11.3 | 1 | 80 |
| IMGA\|Medtr7g101640.1 | Cell division cycle protein 48 homolog | 114.6 (M:114.6) | 5.1 | 72.1 | 4.8 | 2 | 81 |
| IMGA\|Medtr6g026810.1 | 14 kDa zinc-binding protein | 113.1 (M:113.1) | 23.5 | 16.8 | 6.7 | 2 | 82 |
| IMGA\|Medtr7g081660.1 | Transcription factor | 111.9 (M:111.9) | 3.7 | 68 | 5.8 | 2 | 83 |
| RUBB_SECCE | RuBisCO large subunit-binding protein subunit beta, chloroplastic | 111.1 (M:111.1) | 7.8 | 53.4 | 4.7 | 2 | 84 |
| IMGA\|Medtr2g005570.2 | Elongation factor 1-gamma | 110.5 (M:110.5) | 4.5 | 47.7 | 7.7 | 2 | 85 |
| IMGA\|Medtr2g083160.1 | Conglutin | 110.2 (M:110.2) | 24.3 | 16.8 | 6.3 | 2 | 86 |
| ENO_MESCR | Enolase (EC 4.2.1.11) | 109.7 (M:109.7) | 9.7 | 48.3 | 5.5 | 1 | 87 |
| IMGA\|Medtr2g100560.1 | IAA-amino acid hydrolase ILR1-like 4 | 107.5 (M:107.5) | 8.5 | 48.8 | 5.6 | 2 | 88 |

| | | | | | | | |
|---|---|---|---|---|---|---|---|
| RS155_ARATH | 40S ribosomal protein S15-5 | 107.0 (M:107.0) | 12.8 | 16.8 | 11 | 2 | 89 |
| IMGA\|Medtr5g077000.2 | UTP-glucose 1 phosphate uridylyltransferase | 106.4 (M:106.4) | 8.1 | 51.4 | 5.1 | 2 | 90 |
| IMGA\|Medtr1g064060.2 | Adenosine kinase 2 | 105.2 (M:105.2) | 10.4 | 35.1 | 5.6 | 2 | 91 |
| EF1B_ORYSJ | Elongation factor 1-beta | 104.7 (M:104.7) | 7.6 | 23.8 | 4.5 | 2 | 92 |
| IMGA\|Medtr8g092040.1 | Unknown Protein | 103.5 (M:103.5) | 13.9 | 27.7 | 8.8 | 2 | 93 |
| PORB_ARATH | Protochlorophyllide reductase B | 102.0 (M:102.0) | 9 | 43.3 | 9.8 | 2 | 94 |
| IMGA\|Medtr3g005430.1 | 40S ribosomal protein S9 | 101.6 (M:101.6) | 8.6 | 22.9 | 10.7 | 2 | 95 |
| IMGA\|Medtr5g010020.2 | 40S ribosomal protein S10-like | 100.4 (M:100.4) | 4 | 80.6 | 8.6 | 2 | 96 |
| IMGA\|Medtr4g078410.1 | Early nodulin-like protein 2 | 94.3 (M:94.3) | 8.6 | 20.6 | 9.2 | 1 | 97 |
| IMGA\|Medtr8g088370.1 | Chloroplast protein import component Toc159-like (Fragment) | 94.1 (M:94.1) | 3.2 | 149.3 | 4.1 | 2 | 98 |
| IMGA\|Medtr7g111590.3 | 60S ribosomal protein L13 | 93.8 (M:93.8) | 17.3 | 15.7 | 10.8 | 1 | 99 |
| IMGA\|Medtr3g008250.1 | Prohibitin | 92.7 (M:92.7) | 12 | 31 | 9 | 2 | 100 |
| IMGA\|Medtr4g024550.1 | 40S ribosomal protein S13 | 92.2 (M:92.2) | 9.4 | 15.8 | 10.8 | 2 | 101 |
| IMGA\|Medtr7g006560.1 | Transaldolase | 92.0 (M:92.0) | 4.1 | 48.5 | 6.2 | 1 | 102 |
| RL37A_GOSHI | 60S ribosomal protein L37a | 91.4 (M:91.4) | 17.4 | 10.2 | 11.3 | 1 | 103 |
| IMGA\|Medtr1g081410.1 | 40S ribosomal protein S24 | 91.4 (M:91.4) | 17.5 | 15.7 | 11.1 | 2 | 104 |
| IMGA\|Medtr7g099640.1 | 26S proteasome regulatory subunit 4 | 90.5 (M:90.5) | 9 | 49.5 | 5.8 | 2 | 105 |
| TBA_PRUDU | Tubulin alpha chain | 88.3 (M:88.3) | 7.8 | 49.5 | 4.8 | 1 | 106 |
| IMGA\|Medtr7g083560.1 | Translocon-associated protein subunit beta | 85.4 (M:85.4) | 13.9 | 21 | 9.9 | 2 | 107 |
| IMGA\|Medtr5g094210.1 | MtN5 protein | 85.0 (M:85.0) | 26.5 | 10.8 | 10.5 | 2 | 108 |
| RS271_ARATH | 40S ribosomal protein S27-1 | 84.6 (M:84.6) | 21.4 | 9.4 | 10.6 | 2 | 109 |
| IMGA\|Medtr1g019810.1 | Unknown Protein | 83.8 (M:83.8) | 15.4 | 25.7 | 5.7 | 2 | 110 |
| IMGA\|Medtr6g087990.1 | Peroxiredoxin | 83.2 (M:83.2) | 12.3 | 17.5 | 5.5 | 1 | 111 |
| MDHM_IMPCY | Malate dehydrogenase | 83.0 (M:83.0) | 61.3 | 3.1 | 7 | 1 | 112 |
| SODC5_MAIZE | Superoxide dismutase [Cu-Zn] | 82.1 (M:82.1) | 15.1 | 15.1 | 5.6 | 2 | 113 |
| IMGA\|Medtr3g043750.2 | Cysteine proteinase inhibitor | 82.1 (M:82.1) | 15.4 | 11.3 | 10.6 | 1 | 114 |
| IMGA\|Medtr1g092740.1 | GDSL esterase/lipase | 81.6 (M:81.6) | 3.7 | 39.1 | 9.8 | 1 | 115 |
| IMGA\|Medtr3g114850.1 | Plastocyanin | 79.8 (M:79.8) | 28.1 | 17.1 | 4.8 | 2 | 116 |
| IMGA\|Medtr2g010020.1 | Heat shock 70 kDa protein | 79.6 (M:79.6) | 4.1 | 72.4 | 5.4 | 2 | 117 |
| IMGA\|Medtr8g088270.1 | T-complex protein 1 subunit alpha | 78.6 (M:78.6) | 5.1 | 58.8 | 5.8 | 2 | 118 |
| ICDHC_ARATH | Cytosolic isocitrate dehydrogenase | 78.5 (M:78.5) | 4.1 | 45.7 | 6.1 | 1 | 119 |
| IMGA\|Medtr1g114050.1 | Histone H2A | 76.6 (M:76.6) | 14.5 | 14.2 | 10.6 | 2 | 120 |
| LEG_CICAR | Legumin | 76.4 (M:76.4) | 2.2 | 56.2 | 6.2 | 1 | 121 |
| IMGA\|AC235488_11.1 | 40S ribosomal protein SA | 75.6 (M:75.6) | 8.8 | 32.8 | 4.7 | 2 | 122 |
| PSBQ_ONOVI | Oxygen-evolving enhancer protein 3 | 74.7 (M:74.7) | 5.6 | 24.8 | 10 | 1 | 123 |
| IMGA\|Medtr8g081490.1 | Adenosylhomocysteinase | 74.3 (M:74.3) | 5.1 | 49.8 | 5.2 | 1 | 124 |
| IMGA\|Medtr8g091320.3 | Inositol-3-phosphate synthase | 73.7 (M:73.7) | 8.4 | 18.5 | 5.7 | 1 | 125 |
| IMGA\|AC235678_12.1 | Fructose-bisphosphate aldolase | 70.5 (M:70.5) | 9.4 | 16.1 | 10.2 | 1 | 126 |
| IMGA\|Medtr8g036880.1 | ADP,ATP carrier protein 2, mitochondrial | 70.3 (M:70.3) | 2.8 | 43.3 | 10.3 | 1 | 127 |
| IMGA\|AC146630_2.1 | 2-cys peroxiredoxin | 69.1 (M:69.1) | 9.4 | 29 | 6.1 | 1 | 128 |
| RUXG_MEDSA | Probable small nuclear ribonucleoprotein G | 68.4 (M:68.4) | 19.8 | 8.9 | 9.3 | 1 | 129 |
| IMGA\|Medtr3g109190.1 | | 67.7 (M:67.7) | 17.4 | 16.8 | 9.3 | 1 | 130 |
| IMGA\|Medtr7g083720.1 | V-type proton ATPase subunit C | 67.6 (M:67.6) | 4.2 | 42.8 | 5.5 | 1 | 131 |
| IMGA\|Medtr8g046140.2 | 60S ribosomal protein L11 | 67.6 (M:67.6) | 12 | 13.2 | 10.4 | 1 | 132 |
| IMGA\|Medtr3g117040.1 | 40S ribosomal protein S25 | 67.2 (M:67.2) | 14.9 | 10.5 | 10.8 | 1 | 133 |
| IMGA\|Medtr7g076940.1 | 60S ribosomal protein L2 | 66.9 (M:66.9) | 7.3 | 28 | 11.5 | 1 | 134 |
| IMGA\|Medtr2g069050.1 | Elongation factor EF-2 (Fragment) | 66.1 (M:66.1) | 1.9 | 94.1 | 5.7 | 1 | 135 |
| CP18C_ARATH | Peptidyl-prolyl cis-trans isomerase CYP18-3 (EC 5.2.1.8) | 65.8 (M:65.8) | 8.7 | 18.4 | 9 | 1 | 136 |
| IMGA\|Medtr1g094630.1 | 60S ribosomal protein L4 | 65.4 (M:65.4) | 1.7 | 130.9 | 6.5 | 1 | 137 |
| RS281_ARATH | 40S ribosomal protein S28-1 | 65.1 (M:65.1) | 18.8 | 7.4 | 11.3 | 1 | 138 |
| SAR1B_BRACM | GTP-binding protein | 64.2 (M:64.2) | 6.7 | 22.1 | 6.6 | 1 | 139 |
| IMGA\|Medtr4g050400.1 | Unknown Protein | 63.3 (M:63.3) | 13 | 16.4 | 9 | 1 | 140 |
| AAT1_MEDSA | Aspartate aminotransferase 1 (EC 2.6.1.1) | 63.2 (M:63.2) | 4.1 | 45.7 | 9.4 | 1 | 141 |
| IMGA\|Medtr3g114400.1 | Subtilisin-like protease | 63.0 (M:63.0) | 14.6 | 17.8 | 4.9 | 1 | 142 |
| IMGA\|Medtr1g088450.1 | 60S ribosomal protein L22-like | 62.3 (M:62.3) | 10.9 | 13.6 | 10 | 1 | 143 |
| CB28_PEA | Chlorophyll a-b binding protein 8 | 62.0 (M:62.0) | 4.9 | 28.5 | 5 | 1 | 144 |
| IMGA\|Medtr4g075340.1 | Translocon-associated protein subunit alpha | 62.0 (M:62.0) | 5.1 | 27.5 | 4.6 | 1 | 145 |
| IMGA\|Medtr2g006170.1 | 60S ribosomal protein L27 | 61.2 (M:61.2) | 8.1 | 15.6 | 10.8 | 1 | 146 |
| IMGA\|Medtr5g098060.1 | Fasciclin-like arabinogalactan protein 2 | 61.2 (M:61.2) | 6.9 | 27.8 | 5 | 1 | 147 |
| RS5_NICPL | 40S ribosomal protein S5 (Fragment) | 60.9 (M:60.9) | 9.7 | 17.1 | 11.1 | 1 | 148 |
| IMGA\|Medtr4g070600.1 | 40S ribosomal protein S25-2 | 59.9 (M:59.9) | 9.3 | 11.8 | 11.2 | 1 | 149 |
| RBL_PINPS | Ribulose bisphosphate carboxylase large chain | 59.8 (M:59.8) | 18.8 | 5.1 | 3.9 | 1 | 150 |
| IMGA\|Medtr7g092720.1 | 40S ribosomal protein S20-2 | 59.3 (M:59.3) | 9.8 | 13.7 | 10.2 | 1 | 151 |
| IMGA\|Medtr4g101650.1 | Monocopper oxidase-like protein | 59.0 (M:59.0) | 2.7 | 66.2 | 9.4 | 1 | 152 |
| RS30_ARATH | 40S ribosomal protein S30 | 58.3 (M:58.3) | 16.1 | 6.9 | 12.7 | 1 | 153 |
| TCTP_PEA | Translationally-controlled tumor protein homolog | 58.0 (M:58.0) | 12.6 | 18.9 | 4.6 | 1 | 154 |
| PSBO_PEA | Oxygen-evolving enhancer protein 1 | 57.7 (M:57.7) | 7.3 | 34.9 | 6.3 | 1 | 155 |
| IMGA\|Medtr3g013640.2 | 40S ribosomal protein S19 | 56.7 (M:56.7) | 7 | 16 | 10.6 | 1 | 156 |
| IMGA\|Medtr5g008210.1 | Unknown Protein | 56.6 (M:56.6) | 17.6 | 14 | 6.2 | 1 | 157 |
| IMGA\|Medtr3g082200.1 | Subtilisin-like protease | 56.4 (M:56.4) | 1.7 | 83 | 9.3 | 1 | 158 |
| IMGA\|Medtr2g033920.2 | GDSL esterase/lipase | 56.2 (M:56.2) | 6.5 | 23.8 | 9.4 | 1 | 159 |
| IMGA\|Medtr3g079210.1 | Non-specific lipid-transfer protein | 55.8 (M:55.8) | 9.2 | 20.4 | 4.5 | 1 | 160 |
| RL7A_ORYSJ | 60S ribosomal protein L7a | 55.7 (M:55.7) | 4.3 | 29.3 | 11 | 1 | 161 |
| IMGA\|Medtr2g014220.1 | Ribosomal protein L15 | 55.3 (M:55.3) | 5.6 | 29.7 | 12.1 | 1 | 162 |
| ILV5_ORYSJ | Ketol-acid reductoisomerase | 54.8 (M:54.8) | 3.3 | 62.3 | 6 | 1 | 163 |
| IMGA\|Medtr4g128840.2 | Xylose isomerase | 54.8 (M:54.8) | 5.6 | 23.7 | 4.9 | 1 | 164 |
| IMGA\|Medtr1g075320.1 | N-carbamoyl-L-amino acid hydrolase | 54.4 (M:54.4) | 2.6 | 54.1 | 5.6 | 1 | 165 |
| IMGA\|Medtr4g115970.2 | Vacuolar proton-inorganic pyrophosphatase | 53.9 (M:53.9) | 3.1 | 75.6 | 4.9 | 1 | 166 |
| IMGA\|Medtr5g081710.3 | 60S ribosomal protein L35a | 53.9 (M:53.9) | 13.8 | 15.1 | 11.2 | 1 | 167 |
| IMGA\|Medtr3g085210.1 | Non-specific lipid-transfer protein | 53.2 (M:53.2) | 7.8 | 19.9 | 9.4 | 1 | 168 |
| IMGA\|Medtr4g130860.1 | Leucine aminopeptidase 2 | 52.9 (M:52.9) | 3 | 59.6 | 8.6 | 1 | 169 |
| IMGA\|Medtr7g101580.1 | Cell division control protein 48 homolog E | 52.5 (M:52.5) | 9.5 | 26.8 | 10.3 | 1 | 170 |
| IMGA\|AC235665_13.2 | 40S ribosomal protein S3a | 52.3 (M:52.3) | 8.8 | 18.4 | 10.1 | 1 | 171 |
| ALFC2_PEA | Fructose-bisphosphate aldolase 2 | 52.2 (M:52.2) | 8 | 37.8 | 5.4 | 1 | 172 |
| IMGA\|Medtr4g057240.1 | Superoxide dismutase | 51.6 (M:51.6) | 11.9 | 20.7 | 6 | 1 | 173 |
| IMGA\|Medtr4g124660.4 | Sucrose synthase | 50.8 (M:50.8) | 4.4 | 54.2 | 6 | 1 | 174 |
| IMGA\|Medtr5g091120.2 | 60S ribosomal protein L18a | 50.6 (M:50.6) | 12.2 | 17.5 | 11 | 1 | 175 |
| IMGA\|Medtr1g105200.1 | 40S ribosomal protein S15a | 50.5 (M:50.5) | 8.7 | 11.8 | 11.5 | 1 | 176 |
| DUT_ARATH | Deoxyuridine 5'-triphosphate nucleotidohydrolase | 50.4 (M:50.4) | 10.8 | 17.5 | 5.2 | 1 | 177 |
| IMGA\|Medtr3g104280.1 | Far upstream element-binding protein 1 | 50.2 (M:50.2) | 2 | 63 | 5.3 | 1 | 178 |
| IMGA\|Medtr5g097200.1 | 40S ribosomal protein S26 | 50.2 (M:50.2) | 6.5 | 26.1 | 11.5 | 1 | 179 |

| | | | | | | | |
|---|---|---|---|---|---|---|---|
| IMGA\|Medtr7g080090.1 | 60S ribosomal protein L35 | 49.5 (M:49.5) | 10.6 | 14.3 | 11.4 | 1 | 180 |
| IMGA\|Medtr2g098010.1 | Proteasome subunit beta type | 49.0 (M:49.0) | 11.3 | 25.4 | 6.3 | 1 | 181 |
| IMGA\|Medtr8g083520.1 | Aldehyde dehydrogenase | 49.0 (M:49.0) | 2.5 | 52.3 | 5.8 | 1 | 182 |
| IMGA\|Medtr8g027080.1 | Cytochrome b5 | 47.9 (M:47.9) | 16.9 | 15.6 | 4.7 | 1 | 183 |
| IMGA\|Medtr8g079230.1 | Ubiquitin | 47.9 (M:47.9) | 30.8 | 5.8 | 4.9 | 1 | 184 |
| IMGA\|Medtr1g102230.1 | Alpha-L-fucosidase 1 | 47.1 (M:47.1) | 3.5 | 56.1 | 6.2 | 1 | 185 |
| IMGA\|Medtr3g108280.1 | 60S acidic ribosomal protein p0 | 47.0 (M:47.0) | 6.8 | 34.4 | 5.1 | 1 | 186 |
| IMGA\|Medtr2g085330.2 | Unknown Protein | 46.6 (M:46.6) | 3.2 | 67.5 | 9.3 | 1 | 187 |
| IMGA\|Medtr7g098290.2 | 60S ribosomal protein L23 | 46.3 (M:46.3) | 16 | 13.4 | 10.9 | 1 | 188 |
| RL5_HELAN | 60S ribosomal protein L5 | 46.2 (M:46.2) | 6.1 | 33.9 | 10.3 | 1 | 189 |
| RL7A2_ARATH | 60S ribosomal protein L7a-2 | 46.1 (M:46.1) | 4.7 | 29 | 10.7 | 1 | 190 |
| IMGA\|Medtr8g072000.4 | Unknown Protein | 45.7 (M:45.7) | 22.1 | 9.8 | 7.7 | 1 | 191 |
| PSBP1_ARATH | Oxygen-evolving enhancer protein 2-1 | 45.7 (M:45.7) | 5.3 | 28.1 | 7.7 | 1 | 192 |
| IMGA\|Medtr7g092260.1 | Adenylosuccinate synthetase | 45.6 (M:45.6) | 3.9 | 52.9 | 7.9 | 1 | 193 |
| IMGA\|Medtr4g070140.1 | Glycine-rich RNA binding protein 1 | 45.5 (M:45.5) | 5.8 | 18.4 | 5.1 | 1 | 194 |
| IMGA\|Medtr7g009330.1 | Outer plastidial membrane protein porin | 45.5 (M:45.5) | 6.9 | 29.6 | 9.4 | 1 | 195 |
| FLA15_ARATH | Fasciclin-like arabinogalactan protein 15 | 44.5 (M:44.5) | 4.6 | 48 | 6.2 | 1 | 196 |
| ODPB_PEA | Pyruvate dehydrogenase E1 component subunit beta | 44.3 (M:44.3) | 3.1 | 38.8 | 5.9 | 1 | 197 |
| IVD_SOLTU | Isovaleryl-CoA dehydrogenase | 44.2 (M:44.2) | 4.4 | 45.2 | 8.9 | 1 | 198 |
| IMGA\|Medtr5g037960.1 | Unknown Protein | 43.7 (M:43.7) | 3.9 | 47.1 | 5 | 1 | 199 |
| IMGA\|Medtr5g006340.3 | Cysteine synthase | 43.6 (M:43.6) | 9.3 | 19.2 | 5.4 | 1 | 200 |
| RS14_LUPLU | 40S ribosomal protein S14 | 43.5 (M:43.5) | 14 | 16.3 | 11.5 | 1 | 201 |
| RGP1_ARATH | UDP-arabinopyranose mutase 1 | 43.4 (M:43.4) | 5.6 | 40.6 | 5.5 | 1 | 202 |
| IMGA\|Medtr4g071000.1 | Nascent polypeptide-associated complex subunit beta | 43.1 (M:43.1) | 12.5 | 16.5 | 5.7 | 1 | 203 |
| IMGA\|AC233663_14.1 | RuBisCO large subunit-binding protein subunit alpha | 43.1 (M:43.1) | 3.4 | 49.3 | 4.8 | 1 | 204 |
| IMGA\|Medtr1g075790.1 | Os12g0236050 protein (Fragment) | 43.0 (M:43.0) | 1.9 | 111 | 5 | 1 | 205 |
| IMGA\|Medtr8g105340.1 | 40S ribosomal protein S2 | 42.5 (M:42.5) | 5.7 | 30.4 | 11 | 1 | 206 |
| PAPA1_CARPA | Papain precursor (EC 3.4.22.2) | 42.4 (M:42.4) | 4.1 | 38.9 | 7.5 | 1 | 207 |
| IMGA\|Medtr7g013660.1 | Copper transport protein | 42.2 (M:42.2) | 11.3 | 13.4 | 4.4 | 1 | 208 |
| IMGA\|Medtr8g093770.1 | 40S ribosomal protein S12 | 42.2 (M:42.2) | 11.9 | 15.2 | 5.4 | 1 | 209 |
| IMGA\|Medtr8g077810.1 | Cytochrome P450 enzyme | 42.2 (M:42.2) | 1.7 | 46.8 | 9.9 | 1 | 210 |
| SKI24_ARATH | F-box protein SKIP24 | 42.0 (M:42.0) | 2.4 | 34.3 | 10.2 | 1 | 211 |
| IMGA\|Medtr5g091690.1 | Unknown Protein | 41.4 (M:41.4) | 13.7 | 16.7 | 5.8 | 1 | 212 |
| IMGA\|Medtr2g042130.1 | Subtilisin-like protease | 41.4 (M:41.4) | 2.3 | 83 | 9.4 | 1 | 213 |
| IMGA\|Medtr8g102230.1 | Mitochondrial-processing peptidase subunit alpha | 41.4 (M:41.4) | 5.1 | 54.9 | 5.7 | 1 | 214 |
| IMGA\|Medtr4g114770.1 | Methyl binding domain protein (Fragment) | 41.3 (M:41.3) | 4.2 | 38.9 | 4.5 | 1 | 215 |
| IMGA\|Medtr2g094180.1 | Protein disulfide isomerase L-2 | 41.3 (M:41.3) | 2 | 63 | 4.5 | 1 | 216 |
| GATP_ARATH | Gamma-aminobutyrate transaminase | 41.1 (M:41.1) | 2.8 | 55.2 | 8.8 | 1 | 217 |
| IMGA\|Medtr2g029800.3 | Peroxidase | 41.0 (M:41.0) | 6.2 | 15.6 | 5.8 | 1 | 218 |
| IMGA\|Medtr5g011850.1 | EF hand family protein | 40.9 (M:40.9) | 10.9 | 16.6 | 4.5 | 1 | 219 |
| ASSY_ARATH | Argininosuccinate synthase | 40.6 (M:40.6) | 4.5 | 53.8 | 6.3 | 1 | 220 |
| IMGA\|Medtr3g008580.1 | Monocopper oxidase-like protein SKS1 | 40.6 (M:40.6) | 4.6 | 65.7 | 5.6 | 1 | 221 |
| IMGA\|Medtr8g091910.1 | 60S ribosomal protein L6 | 40.4 (M:40.4) | 6.4 | 24.4 | 10.5 | 1 | 222 |
| IMGA\|AC174330_30.1 | Beta-glucosidase 01 | 40.1 (M:40.1) | 6.2 | 38.6 | 9.7 | 1 | 223 |
| IMGA\|Medtr7g069390.1 | Proliferation-associated protein 2G4 | 39.8 (M:39.8) | 4.3 | 43.5 | 6.1 | 1 | 224 |
| IMGA\|Medtr1g061670.1 | 60S ribosomal protein L24 | 39.7 (M:39.7) | 7.4 | 18.4 | 11.4 | 1 | 225 |
| 1433B_VICFA | 14-3-3-like protein B | 39.5 (M:39.5) | 7.7 | 29.5 | 4.6 | 1 | 226 |
| IMGA\|Medtr2g100410.1 | 60S ribosomal protein L28-1 | 39.5 (M:39.5) | 8.9 | 16.4 | 11.4 | 1 | 227 |
| IMGA\|Medtr3g117740.1 | Translocon-associated protein subunit alpha | 39.4 (M:39.4) | 8.5 | 28.1 | 4.8 | 1 | 228 |
| IMGA\|AC235753_1.1 | Cysteine proteinase | 39.4 (M:39.4) | 6.6 | 40.3 | 6.1 | 1 | 229 |
| PSA5A_ARATH | Proteasome subunit alpha type-5-A | 39.2 (M:39.2) | 5.5 | 25.9 | 4.6 | 1 | 230 |
| PDX11_ORYSJ | Probable pyridoxal biosynthesis protein PDX1.1 | 39.1 (M:39.1) | 8.5 | 33.7 | 6.5 | 1 | 231 |
| IMGA\|Medtr3g083280.1 | F-box/LRR-repeat protein 2 | 39.1 (M:39.1) | 1.2 | 73.4 | 9.9 | 1 | 232 |
| IMGA\|Medtr4g063060.1 | 60S ribosomal protein L23a | 38.9 (M:38.9) | 8.6 | 17.1 | 10.7 | 1 | 233 |
| IMGA\|Medtr5g038110.1 | Uncharacterized PI3/PI4-kinase family protein | 38.9 (M:38.9) | 4.2 | 48.7 | 6 | 1 | 234 |
| SYAP_ARATH | Probable alanine--tRNA ligase | 38.6 (M:38.6) | 0.8 | 107.8 | 5.3 | 1 | 235 |
| PSBQ1_ARATH | Oxygen-evolving enhancer protein 3-1 | 38.4 (M:38.4) | 5.4 | 23.9 | 10 | 1 | 236 |
| IMGA\|Medtr7g091960.1 | Calcium-dependent protein kinase 8 | 38.2 (M:38.2) | 21.1 | 10.6 | 9.4 | 1 | 237 |
| IMGA\|Medtr1g023140.1 | Thioredoxin H-type | 38.2 (M:38.2) | 9.2 | 13.3 | 5.1 | 1 | 238 |
| IMGA\|Medtr3g084310.1 | Serine hydroxymethyltransferase | 38.1 (M:38.1) | 3.4 | 51.6 | 7 | 1 | 239 |
| NU5C_AMBTC | NAD(P)H-quinone oxidoreductase subunit 5 | 38.0 (M:38.0) | 2.9 | 83.6 | 8.4 | 1 | 240 |
| IMGA\|Medtr7g114900.1 | Chloroplast ribose-5-phosphate isomerase | 37.8 (M:37.8) | 7.9 | 29.9 | 5.3 | 1 | 241 |
| IMGA\|Medtr7g028670.1 | Unknown Protein | 37.6 (M:37.6) | 6.2 | 39 | 9.8 | 1 | 242 |
| IMGA\|Medtr3g101640.1 | Laccase-15 | 37.4 (M:37.4) | 1.8 | 125.4 | 9.8 | 1 | 243 |
| IMGA\|Medtr5g088250.1 | F-box/LRR-repeat protein 7 | 37.4 (M:37.4) | 3.1 | 61.9 | 8 | 1 | 244 |
| FB49_ARATH | F-box protein | 37.3 (M:37.3) | 4 | 55.2 | 6 | 1 | 245 |
| IMGA\|Medtr5g030940.1 | Oligopeptidase A | 37.3 (M:37.3) | 2.1 | 109.9 | 6 | 1 | 246 |
| IMGA\|Medtr2g039680.1 | Nucleosome assembly protein 1-like | 37.2 (M:37.2) | 4.9 | 41.8 | 4.1 | 1 | 247 |
| IMGA\|Medtr8g088210.1 | Actin depolymerizing factor-like protein | 37.1 (M:37.1) | 15.1 | 16 | 7.6 | 1 | 248 |
| IMGA\|Medtr7g085240.1 | Unknown Protein | 37.0 (M:37.0) | 4.6 | 39.7 | 6.5 | 1 | 249 |
| IMGA\|Medtr5g042880.1 | Argininosuccinate synthase | 37.0 (M:37.0) | 4.6 | 53.1 | 6.5 | 1 | 250 |
| IMGA\|Medtr4g128090.1 | Haloacid dehalogenase-like hydrolase domain | 37.0 (M:37.0) | 7.6 | 33.6 | 6.1 | 1 | 251 |
| IMGA\|Medtr8g038280.1 | Aspartic proteinase nepenthesin-1 | 37.0 (M:37.0) | 3.4 | 47.7 | 4.8 | 1 | 252 |
| IMGA\|AC233577_36.1 | Coatomer subunit delta | 36.7 (M:36.7) | 2.5 | 58.2 | 5.4 | 1 | 253 |
| BRE1A_ARATH | E3 ubiquitin-protein ligase | 36.6 (M:36.6) | 1.5 | 99.7 | 6.5 | 1 | 254 |
| IMGA\|Medtr3g070660.1 | ATP-dependent DNA helicase PIF1 | 36.5 (M:36.5) | 6.6 | 30.2 | 10 | 1 | 255 |
| IMGA\|Medtr1g079530.1 | GDSL esterase/lipase | 36.4 (M:36.4) | 3.6 | 39.6 | 9.4 | 1 | 256 |
| IMGA\|Medtr4g122890.1 | Pectinesterase inhibitor 2 | 36.4 (M:36.4) | 9.7 | 18.9 | 9.1 | 1 | 257 |
| PSA2A_ARATH | Proteasome subunit alpha type-2-A | 36.4 (M:36.4) | 6 | 25.7 | 5.4 | 1 | 258 |
| IMGA\|Medtr4g059450.1 | Protein disulfide isomerase L-2 | 36.4 (M:36.4) | 2.4 | 65.1 | 4.5 | 1 | 259 |
| IMGA\|Medtr5g029270.1 | Unknown Protein | 36.3 (M:36.3) | 38.6 | 7.6 | 5 | 1 | 260 |
| RPOC1_NASOF | DNA-directed RNA polymerase subunit beta | 36.3 (M:36.3) | 1.6 | 78.5 | 9.5 | 1 | 261 |
| IMGA\|Medtr4g124910.2 | 20 kDa chaperonin, chloroplastic | 36.3 (M:36.3) | 13 | 21.7 | 5.7 | 1 | 262 |
| IMGA\|Medtr4g006350.1 | Coatomer subunit beta'-1 | 36.2 (M:36.2) | 1.4 | 105.7 | 5.1 | 1 | 263 |
| IMGA\|Medtr7g101310.1 | Unknown Protein | 36.0 (M:36.0) | 9.7 | 10.5 | 6.1 | 1 | 264 |
| IMGA\|Medtr1g061690.1 | Serine/threonine-protein kinase | 36.0 (M:36.0) | 3.7 | 61 | 6.8 | 1 | 265 |
| IMGA\|Medtr6g052140.1 | 40S ribosomal protein S3 | 36.0 (M:36.0) | 3.4 | 26.2 | 10.4 | 1 | 266 |
| HMT2_MAIZE | Homocysteine S-methyltransferase 2 | 36.0 (M:36.0) | 5.9 | 36.9 | 5 | 1 | 267 |
| GDL51_ARATH | GDSL esterase/lipase | 35.8 (M:35.8) | 6.1 | 40.2 | 6.4 | 1 | 268 |
| IAA31_ORYSJ | Auxin-responsive protein IAA31 | 35.8 (M:35.8) | 13.2 | 20.9 | -1.1 | 1 | 269 |
| RR4_LOPCO | 30S ribosomal protein S4 | 35.8 (M:35.8) | 13.8 | 23.6 | 10.9 | 1 | 270 |

| | | | | | | | |
|---|---|---|---|---|---|---|---|
| IMGA\|Medtr7g083480.1 | Palmitoyltransferase | 35.7 (M:35.7) | 5.5 | 48.8 | 9.6 | 1 | 271 |
| FTSH9_ARATH | Cell division protease | 35.7 (M:35.7) | 2.9 | 87.8 | 8.5 | 1 | 272 |
| IMGA\|Medtr2g020440.1 | Mediator of DNA damage checkpoint | 35.6 (M:35.6) | 2.5 | 129.2 | 6.2 | 1 | 273 |
| IMGA\|Medtr4g107940.1 | Delta-1-pyrroline-5-carboxylate dehydrogenase 1 protein | 35.5 (M:35.5) | 2.4 | 61.3 | 6.4 | 1 | 274 |
| IMGA\|Medtr2g060830.1 | Proteasome subunit alpha type | 35.4 (M:35.4) | 6.1 | 27.2 | 5.6 | 1 | 275 |
| IMGA\|Medtr4g043630.1 | NBS-LRR resistance-like protein 4G (Fragment) | 35.4 (M:35.4) | 0.9 | 143.1 | 6.1 | 1 | 276 |
| IMGA\|Medtr4g108140.2 | Protein IQ-DOMAIN 31 | 35.3 (M:35.3) | 4.9 | 53.9 | 10.4 | 1 | 277 |
| IMGA\|Medtr4g060440.1 | Unknown Protein | 35.1 (M:35.1) | 41.5 | 5.9 | 7.6 | 1 | 278 |

**M. truncatula 12 d**

| Accession | Protein | Scores | S.Coverage | MW [kDa] | pI | Peptides | Rank |
|---|---|---|---|---|---|---|---|
| IMGA\|Medtr7g079780.1 | Convicilin (Fragment) | 613.3 (M:613.3) | 37.3 | 53.2 | 5.5 | 11 | 1 |
| IMGA\|Medtr7g079740.1 | Convicilin (Fragment) | 537.8 (M:537.8) | 30 | 54.2 | 5.6 | 1 | 2 |
| IMGA\|Medtr7g079730.1 | Convicilin (Fragment) | 525.3 (M:525.3) | 25.9 | 53.1 | 5.3 | 3 | 3 |
| IMGA\|Medtr7g079820.1 | Convicilin (Fragment) | 517.3 (M:517.3) | 28.5 | 53.7 | 5.6 | 1 | 4 |
| IMGA\|Medtr1g083960.1 | Calreticulin | 513.0 (M:513.0) | 25.5 | 48.4 | 4.3 | 7 | 5 |
| IMGA\|Medtr4g103920.1 | Glyceraldehyde-3-phosphate dehydrogenase (Fragment) | 425.5 (M:425.5) | 31.6 | 36.6 | 6.7 | 7 | 6 |
| EF1A_MAIZE | Elongation factor 1-alpha | 401.3 (M:401.3) | 19.7 | 49.2 | 9.8 | 7 | 7 |
| MD37D_ARATH | Probable mediator of RNA polymerase II transcription subunit 37c | 390.2 (M:390.2) | 13.6 | 71.3 | 4.9 | 6 | 8 |
| BIP_SPIOL | Luminal-binding protein precursor (BiP) | 385.8 (M:385.8) | 15.7 | 73.5 | 4.9 | 6 | 9 |
| MD37A_ARATH | Mediator of RNA polymerase II transcription subunit 37a | 375.8 (M:375.8) | 15.2 | 73.6 | 4.9 | 1 | 10 |
| IMGA\|Medtr7g024390.1 | Heat shock protein 70 | 372.0 (M:372.0) | 13.7 | 70.9 | 4.9 | 1 | 11 |
| IMGA\|Medtr7g099680.1 | Heat shock protein 70 | 362.2 (M:362.2) | 14.7 | 79.3 | 5.5 | 1 | 12 |
| IMGA\|Medtr6g021800.1 | Elongation factor 1-alpha | 349.4 (M:349.4) | 8.5 | 109.7 | 9.8 | 1 | 13 |
| IMGA\|Medtr3g018780.1 | Annexin-like protein | 323.8 (M:323.8) | 15.9 | 36.2 | 8.7 | 6 | 14 |
| IMGA\|Medtr7g086300.2 | Methionine synthase | 315.0 (M:315.0) | 14.5 | 83.1 | 5.8 | 6 | 15 |
| IMGA\|Medtr2g069050.1 | Elongation factor EF-2 (Fragment) | 311.1 (M:311.1) | 10.6 | 94.1 | 5.7 | 5 | 16 |
| ATPAM_PEA | ATP synthase subunit alpha, mitochondrial | 294.8 (M:294.8) | 13.2 | 55 | 6 | 4 | 17 |
| PDI_MEDSA | Protein disulfide-isomerase precursor (EC 5.3.4.1) | 293.2 (M:293.2) | 20.5 | 57.1 | 4.8 | 6 | 18 |
| IMGA\|Medtr8g085980.1 | Alpha-tubulin | 266.2 (M:266.2) | 17.1 | 49.5 | 4.9 | 4 | 19 |
| IMGA\|Medtr2g066120.1 | Phosphoglycerate kinase | 261.5 (M:261.5) | 22.9 | 42.4 | 5.7 | 5 | 20 |
| IMGA\|Medtr1g108770.2 | ATP synthase subunit beta | 255.8 (M:255.8) | 13.1 | 59.9 | 5.8 | 4 | 21 |
| IMGA\|Medtr1g025430.1 | Endoplasmin homolog | 244.1 (M:244.1) | 8.7 | 94.1 | 4.6 | 5 | 22 |
| IMGA\|Medtr7g052690.1 | Early tobacco anther 1 | 243.9 (M:243.9) | 43 | 16.9 | 4.5 | 4 | 23 |
| BIP2_MAIZE | Luminal-binding protein 2 precursor (BiP2) | 232.2 (M:232.2) | 8.1 | 73 | 5 | 1 | 24 |
| TBA6_ARATH | Tubulin alpha-6 chain | 231.6 (M:231.6) | 14.2 | 49.5 | 4.8 | 1 | 25 |
| G3PC_TAXBA | Glyceraldehyde-3-phosphate dehydrogenase | 223.0 (M:223.0) | 11.5 | 36.6 | 6.5 | 1 | 26 |
| IMGA\|Medtr5g062430.1 | Xylan 1 4-beta-xylosidase | 221.2 (M:221.2) | 8.5 | 87.9 | 9.3 | 4 | 27 |
| IMGA\|AC233663_14.1 | RuBisCO large subunit-binding protein subunit alpha | 220.9 (M:220.9) | 14.3 | 49.3 | 4.8 | 4 | 28 |
| IMGA\|Medtr5g069050.1 | Fructose-bisphosphate aldolase | 220.9 (M:220.9) | 9.6 | 78.3 | 5.7 | 4 | 29 |
| IMGA\|Medtr5g077000.1 | UTP-glucose 1 phosphate uridylyltransferase | 220.4 (M:220.4) | 18.6 | 52.8 | 5.3 | 4 | 30 |
| IMGA\|Medtr5g062650.1 | Unknown Protein | 220.3 (M:220.3) | 8.5 | 86.3 | 9.1 | 4 | 31 |
| IMGA\|Medtr1g064060.2 | Adenosine kinase 2 | 219.2 (M:219.2) | 15.7 | 35.1 | 5.6 | 3 | 32 |
| PSBO_PEA | Oxygen-evolving enhancer protein 1 | 217.7 (M:217.7) | 17.9 | 34.9 | 6.3 | 5 | 33 |
| RUBB_PEA | RuBisCO large subunit-binding protein subunit beta | 213.8 (M:213.8) | 11.8 | 62.9 | 5.8 | 4 | 34 |
| PGKH2_ARATH | Phosphoglycerate kinase 2 | 197.3 (M:197.3) | 13.8 | 49.9 | 9.1 | 1 | 35 |
| HSP7S_PEA | Stromal 70 kDa heat shock-related protein | 196.5 (M:196.5) | 5.2 | 75.5 | 5.1 | 3 | 36 |
| IMGA\|AC146630_2.1 | 2-cys peroxiredoxin BAS1 | 193.8 (M:193.8) | 26 | 29 | 6.1 | 3 | 37 |
| IMGA\|Medtr4g124660.2 | Sucrose synthase | 189.6 (M:189.6) | 8 | 92.2 | 5.8 | 4 | 38 |
| 1433_PEA | 14-3-3-like protein | 189.5 (M:189.5) | 22.7 | 29.3 | 4.6 | 3 | 39 |
| IMGA\|Medtr6g009650.1 | Kunitz-type trypsin inhibitor-like 1 protein | 181.7 (M:181.7) | 26.7 | 22.5 | 4.8 | 4 | 40 |
| MDHC_MEDSA | Malate dehydrogenase, cytoplasmic (EC 1.1.1.37) | 176.3 (M:176.3) | 9.6 | 35.5 | 6.4 | 3 | 41 |
| ENO_RICCO | Enolase (EC 4.2.1.11) | 168.2 (M:168.2) | 12.4 | 47.9 | 5.5 | 3 | 42 |
| ENO2_HEVBR | Enolase 2 (EC 4.2.1.11) | 162.8 (M:162.8) | 8.8 | 47.9 | 5.9 | 1 | 43 |
| IMGA\|Medtr6g021670.1 | 40S ribosomal protein S7-like protein | 162.6 (M:162.6) | 27.2 | 21.9 | 10.3 | 3 | 44 |
| PDIA6_MEDSA | Probable protein disulfide-isomerase A6 precursor (EC 5.3.4.1) | 160.1 (M:160.1) | 14.6 | 40.5 | 5.3 | 3 | 45 |
| IF4A2_NICPL | Eukaryotic initiation factor 4A-2 | 157.7 (M:157.7) | 6.3 | 46.8 | 5.3 | 3 | 46 |
| IMGA\|Medtr3g093110.1 | Ribosomal protein L9 (Fragment) | 153.1 (M:153.1) | 22.8 | 21.7 | 9.9 | 3 | 47 |
| IMGA\|Medtr2g010020.1 | Heat shock 70 kDa protein, mitochondrial | 151.0 (M:151.0) | 5.5 | 72.4 | 5.4 | 2 | 48 |
| IMGA\|Medtr8g072000.1 | Protein notum homolog | 146.0 (M:146.0) | 14.1 | 43.5 | 9.8 | 3 | 49 |
| IMGA\|Medtr4g078410.1 | Early nodulin-like protein 2 | 145.5 (M:145.5) | 20.3 | 20.6 | 9.2 | 3 | 50 |
| IMGA\|Medtr2g095730.1 | Serine carboxypeptidase | 144.1 (M:144.1) | 7.1 | 57.3 | 5.6 | 3 | 51 |
| IMGA\|Medtr7g074570.2 | 2,3-bisphosphoglycerate-independent phosphoglycerate mutase | 141.6 (M:141.6) | 10.8 | 55.3 | 5.3 | 3 | 52 |
| IMGA\|Medtr1g023120.1 | Beta-galactosidase | 137.9 (M:137.9) | 6.7 | 83.1 | 9.2 | 2 | 53 |
| IMGA\|Medtr5g006440.1 | 40S ribosomal protein S3a-like protein | 137.8 (M:137.8) | 7.3 | 29.8 | 10.4 | 2 | 54 |
| IMGA\|Medtr8g086470.1 | Cysteine proteinase 2 (Fragment) | 137.0 (M:137.0) | 11.5 | 40.4 | 5 | 3 | 55 |
| IMGA\|Medtr1g072630.3 | Legumin B | 136.6 (M:136.6) | 10.9 | 52.2 | 5.8 | 3 | 56 |
| IMGA\|Medtr2g038250.1 | 60S ribosomal protein L7-4 | 136.0 (M:136.0) | 11.5 | 28.5 | 10.4 | 2 | 57 |
| IMGA\|Medtr4g070080.1 | Glycine-rich RNA binding protein 1 | 135.5 (M:135.5) | 24.8 | 15.7 | 5.4 | 3 | 58 |
| IMGA\|Medtr3g114420.1 | Subtilisin-type protease | 133.3 (M:133.3) | 5.7 | 82.4 | 8.4 | 2 | 59 |
| IMGA\|Medtr8g106790.1 | Guanine nucleotide-binding protein subunit beta-like protein | 132.1 (M:132.1) | 13.5 | 35.7 | 7.8 | 2 | 60 |
| IMGA\|Medtr8g038210.1 | Annexin-like protein RJ4 | 132.0 (M:132.0) | 10 | 38.6 | 7.1 | 3 | 61 |
| IMGA\|Medtr4g075290.1 | Peptidyl-prolyl cis-trans isomerase | 131.6 (M:131.6) | 32.6 | 18.2 | 9.5 | 3 | 62 |
| TBB3_SOYBN | Tubulin beta chain (Beta tubulin) (Fragment) | 130.1 (M:130.1) | 10.8 | 45.7 | 5.6 | 2 | 63 |
| PORB_ARATH | Protochlorophyllide reductase B | 128.2 (M:128.2) | 6 | 43.3 | 9.8 | 2 | 64 |
| IMGA\|Medtr4g130860.1 | Leucine aminopeptidase 2 | 127.0 (M:127.0) | 10.2 | 59.6 | 8.6 | 3 | 65 |
| IMGA\|Medtr5g088660.1 | Elongation factor 1-beta 1 | 125.5 (M:125.5) | 18.4 | 24.2 | 4.5 | 2 | 66 |
| IMGA\|Medtr7g101640.1 | Cell division cycle protein 48 homolog | 125.1 (M:125.1) | 4.6 | 72.1 | 4.8 | 2 | 67 |
| IMGA\|Medtr7g118060.1 | 60s acidic ribosomal protein P1 | 124.3 (M:124.3) | 44.2 | 11.4 | 4 | 2 | 68 |
| RS24_ARATH | 40S ribosomal protein S2-4 | 120.2 (M:120.2) | 5.8 | 30.1 | 10.9 | 2 | 69 |
| IMGA\|Medtr2g039680.1 | Nucleosome assembly protein 1-like 1 | 118.7 (M:118.7) | 11.7 | 41.8 | 4.1 | 2 | 70 |
| IMGA\|Medtr8g081490.1 | Adenosylhomocysteinase | 117.6 (M:117.6) | 14.3 | 49.8 | 5.2 | 3 | 71 |
| IMGA\|Medtr1g044660.1 | 40S ribosomal protein S3 | 115.2 (M:115.2) | 11.3 | 26.6 | 10.2 | 2 | 72 |
| IMGA\|Medtr1g045410.1 | 60S ribosomal protein L4 | 114.2 (M:114.2) | 7.1 | 44.7 | 10.9 | 2 | 73 |
| IMGA\|Medtr2g098950.1 | Fructokinase | 112.6 (M:112.6) | 6.1 | 35.2 | 5.1 | 1 | 74 |
| PSBB_SOYBN | Photosystem II CP47 chlorophyll apoprotein | 109.5 (M:109.5) | 5.9 | 56 | 6.2 | 2 | 75 |
| IMGA\|Medtr7g083560.1 | Translocon-associated protein subunit beta | 106.8 (M:106.8) | 13.4 | 21 | 9.9 | 2 | 76 |
| IMGA\|Medtr1g061630.2 | Pyruvate kinase | 105.5 (M:105.5) | 7.2 | 56 | 6.5 | 2 | 77 |
| IMGA\|Medtr7g026030.1 | Heat shock protein 70 (Fragment) | 104.9 (M:104.9) | 3.8 | 69.6 | 5.3 | 1 | 78 |
| IMGA\|Medtr4g095360.1 | Subtilisin-like protease | 103.9 (M:103.9) | 5.5 | 79.8 | 6.4 | 2 | 79 |
| HS903_ARATH | Heat shock protein 90-3 | 103.8 (M:103.8) | 5 | 80 | 4.8 | 2 | 80 |
| IMGA\|Medtr6g087990.1 | Peroxiredoxin | 103.5 (M:103.5) | 12.3 | 17.5 | 5.5 | 1 | 81 |
| IMGA\|AC235753_1.1 | Cysteine proteinase | 102.5 (M:102.5) | 10.5 | 40.3 | 6.1 | 2 | 82 |
| IMGA\|Medtr3g088970.1 | Argininosuccinate synthase | 101.5 (M:101.5) | 8 | 52.7 | 6.3 | 2 | 83 |
| CALM_MALDO | Calmodulin | 100.9 (M:100.9) | 20.1 | 16.8 | 4 | 2 | 84 |
| IMGA\|Medtr5g082900.1 | Clathrin heavy chain | 100.1 (M:100.1) | 2.3 | 193.2 | 5.2 | 2 | 85 |
| IMGA\|Medtr1g023210.1 | Cysteine proteinase | 100.1 (M:100.1) | 5.8 | 39.9 | 6.6 | 2 | 86 |
| IMGA\|Medtr2g008050.1 | Actin | 100.0 (M:100.0) | 9 | 41.6 | 5.2 | 2 | 87 |
| CB21_CUCSA | Chlorophyll a-b binding protein of LHCII type I | 97.9 (M:97.9) | 17.6 | 27.2 | 5 | 2 | 88 |

| | | | | | | | |
|---|---|---|---|---|---|---|---|
| IMGA\|Medtr8g075890.1 | Naringenin,2-oxoglutarate 3-dioxygenase (Fragment) | 95.8 (M:95.8) | 11.2 | 41.3 | 5.5 | 2 | 89 |
| IMGA\|Medtr1g090130.1 | Chaperonin CPN60-2 | 95.4 (M:95.4) | 9 | 61.1 | 6.3 | 2 | 90 |
| TBB1_AVESA | Tubulin beta-1 chain (Beta-1 tubulin) (Fragment) | 94.8 (M:94.8) | 11.4 | 43.3 | 4.4 | 1 | 91 |
| IMGA\|Medtr2g094180.1 | Protein disulfide isomerase L-2 | 94.5 (M:94.5) | 6.2 | 63 | 4.5 | 2 | 92 |
| IMGA\|Medtr4g069920.1 | Coatomer subunit gamma | 94.4 (M:94.4) | 2.3 | 98.2 | 4.9 | 1 | 93 |
| IMGA\|Medtr8g012330.3 | Ribosomal protein S8 | 94.1 (M:94.1) | 10.9 | 16 | 11.6 | 1 | 94 |
| H2B2_SOLLC | Histone H2B.2 | 92.3 (M:92.3) | 10.7 | 15.4 | 10.6 | 1 | 95 |
| IMGA\|Medtr6g014480.2 | Phosphorylase | 92.2 (M:92.2) | 5 | 83.9 | 5.2 | 2 | 96 |
| IMGA\|Medtr4g070140.1 | Glycine-rich RNA binding protein 1 | 91.8 (M:91.8) | 16.8 | 18.4 | 5.1 | 2 | 97 |
| FENR1_ORYSJ | Ferredoxin--NADP reductase, leaf isozyme | 91.0 (M:91.0) | 4.4 | 40 | 9.4 | 1 | 98 |
| CB21_GOSHI | Chlorophyll a-b binding protein 151 | 89.8 (M:89.8) | 16.2 | 28.4 | 5.1 | 1 | 99 |
| IMGA\|Medtr4g085540.1 | Poly(A)-binding protein | 89.0 (M:89.0) | 5 | 71 | 9 | 2 | 100 |
| IMGA\|Medtr3g092090.1 | YSL transporter 3 | 87.6 (M:87.6) | 4.6 | 92.9 | 9 | 2 | 101 |
| IMGA\|Medtr7g025060.1 | Unknown Protein | 86.7 (M:86.7) | 15.7 | 22.8 | 6.1 | 2 | 102 |
| IMGA\|Medtr4g095450.1 | Peroxidase | 86.7 (M:86.7) | 7.1 | 35.4 | 10.2 | 2 | 103 |
| IMGA\|Medtr8g014650.1 | Stem 28 kDa glycoprotein | 86.7 (M:86.7) | 7.7 | 29.9 | 6.7 | 2 | 104 |
| IMGA\|Medtr5g018940.1 | 40S ribosomal protein S4 | 86.6 (M:86.6) | 7.2 | 29.9 | 10.8 | 1 | 105 |
| TKTC_CRAPL | Transketolase | 86.5 (M:86.5) | 6.4 | 56.2 | 5.8 | 2 | 106 |
| IMGA\|Medtr8g023140.1 | Alanine aminotransferase 1 | 85.9 (M:85.9) | 4 | 58 | 6.1 | 2 | 107 |
| IMGA\|Medtr8g039540.1 | Aspartic proteinase nepenthesin-1 | 85.9 (M:85.9) | 9.2 | 46.8 | 10.5 | 2 | 108 |
| IMGA\|Medtr3g005430.1 | 40S ribosomal protein S9 | 83.1 (M:83.1) | 13.2 | 22.9 | 10.7 | 2 | 109 |
| IMGA\|Medtr3g092600.1 | 60S acidic ribosomal protein P1 | 82.8 (M:82.8) | 35.5 | 11.1 | 4.1 | 1 | 110 |
| IMGA\|Medtr4g059400.1 | 60S ribosomal protein L12 | 82.0 (M:82.0) | 18.7 | 17.8 | 9.7 | 2 | 111 |
| RL5_HELAN | 60S ribosomal protein L5 | 81.1 (M:81.1) | 6.1 | 33.9 | 10.3 | 1 | 112 |
| ACT2_SOLLC | Actin-51 | 79.8 (M:79.8) | 10.1 | 37.2 | 5.2 | 1 | 113 |
| IMGA\|Medtr2g034720.1 | Beta xylosidase | 79.4 (M:79.4) | 5.2 | 82.6 | 9.6 | 2 | 114 |
| PORA_CUCSA | Protochlorophyllide reductase | 79.4 (M:79.4) | 4.5 | 43 | 9.8 | 1 | 115 |
| IMGA\|Medtr8g038280.1 | Aspartic proteinase nepenthesin-1 | 79.3 (M:79.3) | 3.4 | 47.7 | 4.8 | 1 | 116 |
| IMGA\|Medtr4g114770.1 | Methyl binding domain protein (Fragment) | 78.5 (M:78.5) | 12.4 | 38.9 | 4.5 | 2 | 117 |
| IMGA\|Medtr8g089110.1 | Basic blue protein | 78.2 (M:78.2) | 17.4 | 13 | 10.6 | 1 | 118 |
| IMGA\|Medtr2g005570.2 | Elongation factor 1-gamma | 75.5 (M:75.5) | 4.5 | 47.7 | 7.7 | 1 | 119 |
| IMGA\|Medtr7g076940.1 | 60S ribosomal protein L2 | 74.6 (M:74.6) | 7.3 | 28 | 11.5 | 1 | 120 |
| IMGA\|Medtr7g088680.1 | Nascent polypeptide-associated complex subunit alpha-like | 74.2 (M:74.2) | 7.4 | 22.1 | 4.2 | 1 | 121 |
| IMGA\|Medtr4g131970.1 | RNA-binding protein 42 | 74.0 (M:74.0) | 3.2 | 45.7 | 6.3 | 1 | 122 |
| IMGA\|Medtr5g097320.2 | Heat-shock protein | 73.4 (M:73.4) | 1.8 | 90 | 5.2 | 1 | 123 |
| IMGA\|Medtr6g055020.1 | ATP synthase subunit beta | 72.9 (M:72.9) | 8.9 | 30.1 | 6.2 | 1 | 124 |
| G3PA_TOBAC | Glyceraldehyde-3-phosphate dehydrogenase A | 70.7 (M:70.7) | 6.4 | 41.8 | 6.7 | 1 | 125 |
| UGDH1_ORYSJ | UDP-glucose 6-dehydrogenase 1 | 70.2 (M:70.2) | 2.5 | 52.3 | 5.7 | 1 | 126 |
| IMGA\|Medtr8g058480.1 | Zinc finger CCCH domain-containing protein 51 | 69.3 (M:69.3) | 7 | 39 | 4.7 | 1 | 127 |
| IMGA\|Medtr2g020360.1 | Acyl carrier protein 1, chloroplastic | 69.0 (M:69.0) | 4.3 | 37.9 | 5.3 | 1 | 128 |
| ILV5_ORYSJ | Ketol-acid reductoisomerase | 67.9 (M:67.9) | 3.5 | 62.3 | 6 | 1 | 129 |
| IMGA\|Medtr4g122890.1 | Pectinesterase inhibitor 2 | 67.3 (M:67.3) | 10.3 | 18.9 | 9.1 | 1 | 130 |
| IMGA\|Medtr8g092040.1 | Unknown Protein | 67.1 (M:67.1) | 7.4 | 27.7 | 8.8 | 1 | 131 |
| IMGA\|Medtr1g035230.1 | Pyrophosphate--fructose 6-phosphate 1-phosphotransferase alpha subunit | 66.6 (M:66.6) | 2.5 | 65.1 | 6.2 | 1 | 132 |
| IMGA\|Medtr7g111590.3 | 60S ribosomal protein L13 | 66.6 (M:66.6) | 17.3 | 15.7 | 10.8 | 1 | 133 |
| IMGA\|Medtr3g108280.1 | 60S acidic ribosomal protein p0 | 66.0 (M:66.0) | 6.8 | 34.4 | 5.1 | 1 | 134 |
| IMGA\|Medtr3g085740.1 | Beta-oxoacyl-ACP reductase (Fragment) | 65.8 (M:65.8) | 7.2 | 33.5 | 9.9 | 1 | 135 |
| IMGA\|Medtr4g074930.2 | RNA-binding protein 34 | 65.6 (M:65.6) | 1.9 | 65.7 | 4.8 | 1 | 136 |
| IMGA\|Medtr3g008580.1 | Monocopper oxidase-like protein | 64.2 (M:64.2) | 2.7 | 65.7 | 5.6 | 1 | 137 |
| IMGA\|Medtr7g113470.1 | T-complex protein 1 subunit beta | 62.6 (M:62.6) | 4.7 | 56.9 | 5.3 | 1 | 138 |
| RS281_ARATH | 40S ribosomal protein S28-1 | 62.1 (M:62.1) | 18.8 | 7.4 | 11.3 | 1 | 139 |
| IMGA\|Medtr8g088370.1 | Chloroplast protein import component Toc159-like (Fragment) | 61.6 (M:61.6) | 1 | 149.3 | 4.1 | 1 | 140 |
| IMGA\|Medtr4g061140.1 | Cytosolic ascorbate peroxidase | 61.5 (M:61.5) | 7.2 | 27.1 | 5.5 | 1 | 141 |
| IMGA\|Medtr8g086070.1 | Mitochondrial 2-oxoglutarate/malate carrier protein | 61.4 (M:61.4) | 4 | 32.2 | 0 | 1 | 142 |
| IMGA\|Medtr2g098010.1 | Proteasome subunit beta type | 61.0 (M:61.0) | 11.3 | 25.4 | 6.3 | 1 | 143 |
| IMGA\|Medtr4g078780.2 | 26S proteasome non-ATPase regulatory subunit 2 | 60.4 (M:60.4) | 1.3 | 84.1 | 4.9 | 1 | 144 |
| IMGA\|Medtr7g081660.1 | Transcription factor | 59.9 (M:59.9) | 3.7 | 68 | 5.8 | 1 | 145 |
| IMGA\|Medtr1g094630.1 | 60S ribosomal protein L4 | 59.6 (M:59.6) | 1.7 | 130.9 | 6.5 | 1 | 146 |
| IMGA\|Medtr3g092980.2 | Nascent polypeptide-associated complex alpha subunit-like protein | 59.1 (M:59.1) | 7.8 | 23.6 | 4.2 | 1 | 147 |
| IMGA\|Medtr8g046140.2 | 60S ribosomal protein L11 | 58.9 (M:58.9) | 12 | 13.2 | 10.4 | 1 | 148 |
| IMGA\|Medtr1g106900.1 | 40S ribosomal protein S18 | 58.7 (M:58.7) | 13.2 | 17.6 | 11.3 | 1 | 149 |
| IMGA\|Medtr3g092900.1 | Caffeic acid O-methyltransferase (Fragment) | 58.0 (M:58.0) | 3.3 | 39.9 | 5.6 | 1 | 150 |
| IMGA\|Medtr5g098770.1 | Chlorophyll a-b binding protein 8 | 57.7 (M:57.7) | 27.7 | 10.3 | 9.9 | 1 | 151 |
| ENO_ORYSJ | Enolase | 56.7 (M:56.7) | 5.6 | 47.9 | 5.3 | 1 | 152 |
| RS271_ARATH | 40S ribosomal protein S27-1 | 56.6 (M:56.6) | 21.4 | 9.4 | 10.6 | 1 | 153 |
| IMGA\|Medtr8g068120.1 | Rhamnogalacturonate lyase | 56.2 (M:56.2) | 3.3 | 79.5 | 6.7 | 1 | 154 |
| IMGA\|Medtr5g024680.1 | Unknown Protein | 55.8 (M:55.8) | 9.6 | 17.6 | 9.6 | 1 | 155 |
| IMGA\|Medtr2g025020.1 | Pyrophosphate--fructose 6-phosphate 1-phosphotransferase subunit | 55.4 (M:55.4) | 4.4 | 61.5 | 5.8 | 1 | 156 |
| IMGA\|AC233657_22.1 | Chlorophyll a-b binding protein | 55.4 (M:55.4) | 9.7 | 28.8 | 6.5 | 1 | 157 |
| RCA_ARAHY | Ribulose bisphosphate carboxylase/oxygenase activase | 55.3 (M:55.3) | 13.6 | 15 | 4.2 | 1 | 158 |
| IMGA\|Medtr8g101330.1 | Biotin carboxylase | 55.3 (M:55.3) | 3.9 | 59 | 8.9 | 1 | 159 |
| IMGA\|Medtr7g069390.1 | Proliferation-associated protein 2G4 | 55.1 (M:55.1) | 4.3 | 43.5 | 6.1 | 1 | 160 |
| IMGA\|Medtr5g031300.1 | Cinnamyl alcohol dehydrogenase | 54.5 (M:54.5) | 3.3 | 84.5 | 6.3 | 1 | 161 |
| IMGA\|Medtr7g081700.1 | Ras-like protein (Fragment) | 53.3 (M:53.3) | 7.9 | 22.5 | 4.8 | 1 | 162 |
| VATB2_HORVU | Vacuolar ATP synthase subunit B isoform 2 (EC 3.6.3.14) | 53.1 (M:53.1) | 3.1 | 53.7 | 5 | 1 | 163 |
| IMGA\|Medtr6g071090.1 | Translationally-controlled tumor protein homolog | 51.8 (M:51.8) | 5.6 | 18.2 | 4.6 | 1 | 164 |
| IMGA\|Medtr4g078200.1 | Prohibitin 1-like protein | 51.1 (M:51.1) | 8.5 | 32.3 | 9.7 | 1 | 165 |
| PGMC_POPTN | Phosphoglucomutase | 51.1 (M:51.1) | 4 | 63.1 | 5.4 | 1 | 166 |
| IMGA\|Medtr4g050400.1 | Unknown Protein | 51.1 (M:51.1) | 13 | 16.4 | 9 | 1 | 167 |
| IMGA\|Medtr1g095830.2 | ATP synthase subunit d, mitochondrial | 49.3 (M:49.3) | 8.3 | 16.8 | 5.5 | 1 | 168 |
| RL171_HORVU | 60S ribosomal protein L17-1 | 49.2 (M:49.2) | 5.3 | 19.5 | 10.9 | 1 | 169 |
| IMGA\|Medtr2g103730.1 | Phospholipase D alpha 1 | 48.7 (M:48.7) | 3 | 92 | 5.4 | 1 | 170 |
| H2A3_VOLCA | Histone H2A-III | 48.4 (M:48.4) | 7 | 13.5 | 10.7 | 1 | 171 |
| RAN_POPEU | GTP-binding nuclear protein Ran (Fragments) | 48.1 (M:48.1) | 50 | 3.3 | 9.5 | 1 | 172 |
| RS14_LUPLU | 40S ribosomal protein S14 | 48.0 (M:48.0) | 8.7 | 16.3 | 11.5 | 1 | 173 |
| IMGA\|Medtr3g114850.1 | Plastocyanin | 47.9 (M:47.9) | 14.4 | 17.1 | 4.8 | 1 | 174 |
| IMGA\|Medtr3g098430.1 | Calnexin homolog | 47.9 (M:47.9) | 3.1 | 61.7 | 4.6 | 1 | 175 |
| IMGA\|Medtr3g084410.1 | Selenium binding protein | 47.7 (M:47.7) | 4.9 | 54.2 | 5.6 | 1 | 176 |
| IMGA\|Medtr4g092620.1 | LL-diaminopimelate aminotransferase | 47.6 (M:47.6) | 5 | 49.9 | 7.7 | 1 | 177 |
| 1433B_VICFA | 14-3-3-like protein B | 47.3 (M:47.3) | 7.7 | 29.5 | 4.6 | 1 | 178 |
| IMGA\|Medtr4g132360.2 | Aminomethyltransferase | 47.0 (M:47.0) | 6.4 | 38.5 | 8.7 | 1 | 179 |

| | | | | | | | |
|---|---|---|---|---|---|---|---|
| IMGA\|Medtr5g098310.2 | Acetyl-CoA acetyltransferase | 46.7 (M:46.7) | 6.3 | 39 | 6.2 | 1 | 180 |
| IMGA\|Medtr5g098060.1 | Fasciclin-like arabinogalactan protein 2 | 46.2 (M:46.2) | 6.9 | 27.8 | 5 | 1 | 181 |
| SODM_PRUPE | Superoxide dismutase [Mn] | 45.9 (M:45.9) | 6.6 | 25.4 | 9.1 | 1 | 182 |
| IMGA\|Medtr5g078200.1 | Glucan endo-1,3-beta-glucosidase 2 | 45.8 (M:45.8) | 3.6 | 69.2 | 4.7 | 1 | 183 |
| IMGA\|Medtr1g098170.1 | 40S ribosomal protein S18 | 45.5 (M:45.5) | 13.2 | 17.6 | 11.3 | 1 | 184 |
| RR4_TAKLE | 30S ribosomal protein S4, chloroplastic | 45.2 (M:45.2) | 3.5 | 23.4 | 11.1 | 1 | 185 |
| ICDHC_ARATH | Cytosolic isocitrate dehydrogenase | 44.9 (M:44.9) | 4.1 | 45.7 | 6.1 | 1 | 186 |
| IMGA\|Medtr8g032300.1 | Elicitor-responsive protein 3 | 44.9 (M:44.9) | 13.5 | 16.8 | 4.3 | 1 | 187 |
| PSBP_PEA | Oxygen-evolving enhancer protein 2 | 44.7 (M:44.7) | 8.1 | 28 | 9.1 | 1 | 188 |
| SODC5_MAIZE | Superoxide dismutase [Cu-Zn] | 44.6 (M:44.6) | 6.6 | 15.1 | 5.6 | 1 | 189 |
| IMGA\|Medtr5g083170.1 | Ferritin-2, chloroplastic | 44.2 (M:44.2) | 9.6 | 27.9 | 5.7 | 1 | 190 |
| IMGA\|Medtr1g075320.1 | N-carbamoyl-L-amino acid hydrolase | 43.9 (M:43.9) | 2.6 | 54.1 | 5.6 | 1 | 191 |
| IMGA\|Medtr4g057240.1 | Superoxide dismutase | 43.8 (M:43.8) | 11.9 | 20.7 | 6 | 1 | 192 |
| IMGA\|Medtr7g084150.1 | Ubiquinol-cytochrome c reductase iron-sulfur subunit | 43.6 (M:43.6) | 7 | 30.2 | 9.7 | 1 | 193 |
| IMGA\|Medtr4g115970.2 | Vacuolar proton-inorganic pyrophosphatase | 43.4 (M:43.4) | 3.1 | 75.6 | 4.9 | 1 | 194 |
| IMGA\|Medtr5g079460.1 | Fructokinase-2 | 42.8 (M:42.8) | 5.7 | 41.4 | 5.1 | 1 | 195 |
| IMGA\|AC225507_16.1 | Eukaryotic translation initiation factor 3 subunit L | 42.5 (M:42.5) | 4.3 | 60.2 | 5.8 | 1 | 196 |
| IMGA\|Medtr6g005820.3 | ADP-ribosylation factor | 42.4 (M:42.4) | 8.5 | 18.7 | 5.5 | 1 | 197 |
| IMGA\|Medtr2g026060.1 | Inositol monophosphatase 3 | 42.1 (M:42.1) | 7.4 | 28.9 | 5.2 | 1 | 198 |
| IMGA\|Medtr1g075790.1 | Os12g0236050 protein (Fragment) | 42.1 (M:42.1) | 1.9 | 111 | 5 | 1 | 199 |
| RL37A_GOSHI | 60S ribosomal protein L37a | 41.6 (M:41.6) | 17.4 | 10.2 | 11.3 | 1 | 200 |
| MATK_LYOLU | Maturase K | 41.6 (M:41.6) | 4.3 | 60.8 | 9.9 | 1 | 201 |
| FBL9_ARATH | Putative F-box/LRR-repeat protein 9 - | 41.5 (M:41.5) | 7.7 | 27.4 | 9.3 | 1 | 202 |
| IMGA\|Medtr3g084310.1 | Serine hydroxymethyltransferase | 41.5 (M:41.5) | 3.4 | 51.6 | 7 | 1 | 203 |
| ALFC3_ARATH | Probable fructose-bisphosphate aldolase 3 | 41.4 (M:41.4) | 7.2 | 42.3 | 9 | 1 | 204 |
| RS172_ARATH | 40S ribosomal protein S17-2 | 41.2 (M:41.2) | 8.6 | 15.9 | 10.5 | 1 | 205 |
| IMGA\|Medtr4g112670.1 | Strictosidine synthase 1 | 41.2 (M:41.2) | 5.1 | 35.5 | 6.2 | 1 | 206 |
| IMGA\|Medtr6g026810.1 | 14 kDa zinc-binding protein (Fragment) | 41.0 (M:41.0) | 12.4 | 16.8 | 6.7 | 1 | 207 |
| AB20I_ARATH | ABC transporter I family member 20 | 40.9 (M:40.9) | 3.4 | 36.9 | 9.4 | 1 | 208 |
| IMGA\|Medtr3g104720.1 | Puromycin-sensitive aminopeptidase | 40.8 (M:40.8) | 1.4 | 98.9 | 5.4 | 1 | 209 |
| IMGA\|Medtr7g074360.1 | Unknown Protein | 40.7 (M:40.7) | 11.1 | 17.7 | 9.8 | 1 | 210 |
| IMGA\|Medtr4g103800.1 | Adenylate kinase B | 40.7 (M:40.7) | 6.2 | 26.7 | 8.8 | 1 | 211 |
| IMGA\|Medtr2g096660.1 | UDP-glucuronic acid decarboxylase 3 | 40.3 (M:40.3) | 4 | 39.6 | 6.9 | 1 | 212 |
| IMGA\|Medtr8g074010.1 | Phosphoserine aminotransferase | 40.3 (M:40.3) | 4.2 | 47.2 | 9.1 | 1 | 213 |
| GL3_ARATH | Transcription factor GLABRA 3 | 40.0 (M:40.0) | 4.7 | 70.5 | 5.7 | 1 | 214 |
| IMGA\|AC225481_15.1 | Subtilisin-like protease | 39.9 (M:39.9) | 3.6 | 79.6 | 9.6 | 1 | 215 |
| IMGA\|Medtr5g087360.1 | Receptor protein kinase-like protein | 39.6 (M:39.6) | 1.5 | 185.6 | 6.4 | 1 | 216 |
| KAD1_ARATH | Adenylate kinase 1 | 39.3 (M:39.3) | 2.8 | 31 | 6.3 | 1 | 217 |
| IMGA\|Medtr6g088250.1 | Elongation factor Ts | 39.3 (M:39.3) | 0.9 | 277.1 | 5.6 | 1 | 218 |
| IMGA\|Medtr1g115880.1 | Chalcone--flavonone isomerase 2 | 39.3 (M:39.3) | 23.6 | 9.3 | 5.2 | 1 | 219 |
| IMGA\|Medtr2g086140.1 | Histone transcription regulator HIRA | 39.2 (M:39.2) | 2.3 | 108.2 | 9 | 1 | 220 |
| RSSA2_ARATH | 40S ribosomal protein Sa-2 | 38.7 (M:38.7) | 2.9 | 30.6 | 4.9 | 1 | 221 |
| IMGA\|Medtr2g076590.1 | At1g78850-like protein (Fragment) | 38.6 (M:38.6) | 4.3 | 49.4 | 5.1 | 1 | 222 |
| IMGA\|Medtr5g033920.1 | ATP-citrate synthase | 38.5 (M:38.5) | 5.7 | 46.6 | 5.5 | 1 | 223 |
| RAVL3_ARATH | AP2/ERF and B3 domain-containing transcription factor | 38.4 (M:38.4) | 7.7 | 40.3 | 6.9 | 1 | 224 |
| IMGA\|Medtr2g102180.1 | 97 kDa heat shock protein | 38.4 (M:38.4) | 1.5 | 95.2 | 5 | 1 | 225 |
| ADHX_PEA | Alcohol dehydrogenase class 3 (EC 1.1.1.1) | 38.4 (M:38.4) | 2.9 | 40.5 | 6.3 | 1 | 226 |
| RS5_NICPL | 40S ribosomal protein S5 (Fragment) | 38.4 (M:38.4) | 9.7 | 17.1 | 11.1 | 1 | 227 |
| AGO9_ARATH | Protein argonaute 9 | 38.3 (M:38.3) | 1.2 | 100.5 | 9.8 | 1 | 228 |
| IMGA\|Medtr2g065470.3 | Glyceraldehyde-3-phosphate dehydrogenase | 38.1 (M:38.1) | 6.1 | 41.7 | 9.6 | 1 | 229 |
| IMGA\|Medtr2g104370.1 | Ribonuclease S3 (Fragment) | 38.0 (M:38.0) | 4.5 | 23.6 | 9.8 | 1 | 230 |
| IMGA\|Medtr3g065330.1 | Nudix hydrolase 18, mitochondrial | 37.9 (M:37.9) | 11.3 | 22.5 | 9.6 | 1 | 231 |
| IMGA\|Medtr2g063000.1 | Serine carboxypeptidase II-2 (Fragment) | 37.9 (M:37.9) | 4.2 | 53.4 | 5.6 | 1 | 232 |
| H4_ARATH | Histone H4 | 37.7 (M:37.7) | 11.7 | 11.4 | 12 | 1 | 233 |
| IMGA\|Medtr2g089860.1 | Subtilisin-like protease | 37.5 (M:37.5) | 3.5 | 81.8 | 8.7 | 1 | 234 |
| IMGA\|Medtr4g012920.1 | At1g15240-like protein (Fragment) | 37.4 (M:37.4) | 1.7 | 137.3 | 8.9 | 1 | 235 |
| CLPA_POPEU | ATP-dependent Clp protease ATP-binding subunit clpA homolog | 37.2 (M:37.2) | 13.9 | 7.9 | 4.1 | 1 | 236 |
| 4CL1_ARATH | 4-coumarate--CoA ligase 1 (EC 6.2.1.12) | 37.2 (M:37.2) | 2.5 | 61 | 5.1 | 1 | 237 |
| ARFS_ORYSJ | Auxin response factor 19 | 37.0 (M:37.0) | 1.6 | 128.2 | 6.1 | 1 | 238 |
| IMGA\|Medtr3g087590.3 | L-myo inositol-1 phosphate synthase 1 | 36.8 (M:36.8) | 3.3 | 47.3 | 5.3 | 1 | 239 |
| Y5600_ARATH | BTB/POZ domain-containing protein | 36.7 (M:36.7) | 1.4 | 56.9 | 6.1 | 1 | 240 |
| IMGA\|Medtr4g132110.2 | Peroxidase | 36.6 (M:36.6) | 4.2 | 37.9 | 9.5 | 1 | 241 |
| IMGA\|Medtr3g055530.1 | Unknown Protein | 36.5 (M:36.5) | 27.6 | 10.2 | 9.6 | 1 | 242 |
| IMGA\|Medtr5g011250.1 | Leucoanthocyanidin dioxygenase (Fragment) | 36.4 (M:36.4) | 4.2 | 40.4 | 5.9 | 1 | 243 |
| IMGA\|Medtr4g031040.1 | Peroxidase 7 | 36.4 (M:36.4) | 7.4 | 15.1 | 4.2 | 1 | 244 |
| IMGA\|Medtr2g094570.1 | Auxin response factor 3 | 36.4 (M:36.4) | 3.4 | 84 | 9.5 | 1 | 245 |
| IMGA\|Medtr4g015570.1 | Chlorophyll a-b binding protein | 36.3 (M:36.3) | 4 | 38 | 8.8 | 1 | 246 |
| IMGA\|Medtr5g092030.1 | Pentatricopeptide repeat-containing protein | 36.2 (M:36.2) | 3 | 81.7 | 6.8 | 1 | 247 |
| IMGA\|Medtr8g079230.1 | Ubiquitin | 36.2 (M:36.2) | 30.8 | 5.8 | 4.9 | 1 | 248 |
| RL15_PETHY | 60S ribosomal protein L15 | 36.2 (M:36.2) | 7.8 | 24.1 | 12 | 1 | 249 |
| IMGA\|Medtr5g046030.1 | Alpha-1 4-glucan-protein synthase | 36.0 (M:36.0) | 3.3 | 41.2 | 5.7 | 1 | 250 |
| IMGA\|Medtr8g039320.1 | Unknown Protein | 36.0 (M:36.0) | 11.1 | 22.6 | 10.2 | 1 | 251 |
| IMGA\|Medtr5g029550.1 | Shikimate kinase-like protein | 35.9 (M:35.9) | 4.5 | 41.3 | 5.2 | 1 | 252 |
| IMGA\|AC235005_4.1 | Cysteine-rich receptor-like protein kinase 26 | 35.9 (M:35.9) | 4.3 | 60.5 | 7.7 | 1 | 253 |
| CHLI2_ARATH | Magnesium-chelatase subunit | 35.8 (M:35.8) | 3.8 | 46.1 | 5.2 | 1 | 254 |
| IMGA\|Medtr4g071130.1 | Coatomer alpha subunit-like protein | 35.7 (M:35.7) | 1.3 | 135.4 | 6.5 | 1 | 255 |
| RL24_HORVU | 60S ribosomal protein L24 | 35.7 (M:35.7) | 7.4 | 18.4 | 11.4 | 1 | 256 |
| PROF1_MAIZE | Profilin-1 | 35.7 (M:35.7) | 19.8 | 14.1 | 4.8 | 1 | 257 |
| SAC4_ARATH | Phosphoinositide phosphatase | 35.5 (M:35.5) | 1.4 | 94 | 6.5 | 1 | 258 |
| IMGA\|Medtr2g018730.1 | Unknown Protein | 35.5 (M:35.5) | 9.8 | 21.8 | 10 | 1 | 259 |
| IMGA\|Medtr1g086540.1 | Pectinesterase (Fragment) | 35.4 (M:35.4) | 4.3 | 38.8 | 9.6 | 1 | 260 |
| IMGA\|Medtr3g098420.1 | Staphylococcal nuclease domain-containing protein | 35.3 (M:35.3) | 2.8 | 108.2 | 6.6 | 1 | 261 |
| IMGA\|Medtr7g113640.1 | Uncharacterized GPI-anchored protein | 35.3 (M:35.3) | 9.7 | 21.4 | 5.4 | 1 | 262 |
| IF5_PHAVU | Eukaryotic translation initiation factor 5 (eIF-5) | 35.0 (M:35.0) | 5.9 | 48.9 | 8.5 | 1 | 263 |

**M. orbicularis 12 d**

| Accession | Protein | Scores | S.Coverage | MW [kDa] | pI | Peptides | Rank |
|---|---|---|---|---|---|---|---|
| IMGA\|Medtr6g021800.1 | Elongation factor 1-alpha | 626.4 (M:626.4) | 14.1 | 109.7 | 9.8 | 12 | 1 |
| IMGA\|Medtr4g103920.1 | Glyceraldehyde-3-phosphate dehydrogenase (Fragment) | 624.4 (M:624.4) | 38.9 | 36.6 | 6.7 | 9 | 2 |
| EF1A_MAIZE | Elongation factor 1-alpha (EF-1-alpha) | 596.0 (M:596.0) | 23.3 | 49.2 | 9.8 | 3 | 3 |
| IMGA\|Medtr1g108770.2 | ATP synthase subunit beta | 510.7 (M:510.7) | 29.2 | 59.9 | 5.8 | 10 | 4 |
| IMGA\|Medtr5g096430.1 | Heat shock protein 90 (Fragment) | 447.4 (M:447.4) | 18.5 | 80.1 | 4.8 | 8 | 5 |
| IMGA\|Medtr3g085850.1 | Glyceraldehyde 3-phosphate dehydrogenase | 443.9 (M:443.9) | 41.8 | 37 | 7.7 | 3 | 6 |
| IMGA\|Medtr2g066120.1 | Phosphoglycerate kinase | 415.6 (M:415.6) | 26.7 | 42.4 | 5.7 | 7 | 7 |
| BIP_SPIOL | Luminal-binding protein precursor (BiP) | 380.4 (M:380.4) | 11.5 | 73.5 | 4.9 | 6 | 8 |
| IMGA\|Medtr2g069310.1 | Elongation factor EF-2 (Fragment) | 375.1 (M:375.1) | 12.1 | 94.1 | 5.7 | 7 | 9 |
| EF1A_MANES | Elongation factor 1-alpha (EF-1-alpha) | 366.2 (M:366.2) | 23.2 | 49.3 | 9.8 | 1 | 10 |
| IMGA\|Medtr1g025430.1 | Endoplasmin homolog | 364.8 (M:364.8) | 12.5 | 94.1 | 4.6 | 6 | 11 |
| IMGA\|Medtr5g077000.1 | UTP-glucose 1 phosphate uridylyltransferase | 361.8 (M:361.8) | 23 | 52.8 | 5.3 | 6 | 12 |
| PDIA6_MEDSA | Probable protein disulfide-isomerase A6 precursor (EC 5.3.4.1) | 354.7 (M:354.7) | 21.4 | 40.5 | 5.3 | 5 | 13 |
| IMGA\|Medtr7g024580.1 | Heat shock protein 70 | 349.6 (M:349.6) | 14 | 71 | 4.9 | 6 | 14 |
| IMGA\|Medtr7g086300.2 | Methionine synthase | 346.1 (M:346.1) | 12.6 | 83.1 | 5.8 | 5 | 15 |
| MD37D_ARATH | Probable mediator of RNA polymerase II transcription subunit 37c | 329.6 (M:329.6) | 13.9 | 71.3 | 4.9 | 1 | 16 |
| IMGA\|Medtr1g064060.2 | Adenosine kinase 2 | 317.9 (M:317.9) | 20.4 | 35.1 | 5.6 | 5 | 17 |
| IMGA\|Medtr7g099680.1 | Heat shock protein 70 | 316.2 (M:316.2) | 12.8 | 79.3 | 5.5 | 1 | 18 |
| IMGA\|Medtr1g087520.1 | Protein disulfide-isomerase | 310.6 (M:310.6) | 22.8 | 41.3 | 5.4 | 1 | 19 |
| IMGA\|Medtr3g018780.1 | Annexin-like protein RJ4 | 308.2 (M:308.2) | 14.6 | 36.2 | 8.7 | 5 | 20 |
| IMGA\|Medtr5g062540.1 | Beta-glucosidase | 297.3 (M:297.3) | 9.3 | 39.5 | 6 | 4 | 21 |
| CALR_NICPL | Calreticulin | 294.3 (M:294.3) | 13.5 | 47.5 | 4.3 | 5 | 22 |
| CALR_RICCO | Calreticulin | 292.1 (M:292.1) | 14.5 | 47.5 | 4.2 | 1 | 23 |
| ENO_RICCO | Enolase (EC 4.2.1.11) | 291.4 (M:291.4) | 18.9 | 47.9 | 5.5 | 4 | 24 |
| IMGA\|Medtr8g081490.1 | Adenosylhomocysteinase | 277.2 (M:277.2) | 23.6 | 49.8 | 5.2 | 5 | 25 |
| H2B_PEA | Histone H2B (Fragments) | 275.4 (M:275.4) | 12.5 | 13.5 | 10.7 | 3 | 26 |
| PDI_MEDSA | Protein disulfide-isomerase precursor (EC 5.3.4.1) | 274.9 (M:274.9) | 12.5 | 57.1 | 4.8 | 5 | 27 |
| IMGA\|Medtr1g083960.1 | Calreticulin | 271.2 (M:271.2) | 18 | 48.4 | 4.3 | 1 | 28 |
| IMGA\|Medtr5g069050.1 | Fructose-bisphosphate aldolase | 268.1 (M:268.1) | 9.2 | 78.3 | 5.7 | 4 | 29 |
| IMGA\|Medtr1g043040.1 | Malate dehydrogenase | 258.0 (M:258.0) | 19.3 | 35.5 | 6.1 | 5 | 30 |
| IMGA\|Medtr3g087590.3 | L-myo inositol-1 phosphate synthase 1 | 250.6 (M:250.6) | 16.1 | 47.3 | 5.3 | 5 | 31 |
| IMGA\|Medtr2g094180.1 | Protein disulfide isomerase L-2 | 249.1 (M:249.1) | 11.9 | 63 | 4.5 | 5 | 32 |
| IMGA\|Medtr5g062430.1 | Xylan 1 4-beta-xylosidase | 238.9 (M:238.9) | 6.6 | 87.9 | 9.3 | 3 | 33 |
| IMGA\|Medtr4g095360.1 | Subtilisin-like protease | 233.4 (M:233.4) | 10 | 79.8 | 6.4 | 4 | 34 |
| IMGA\|Medtr4g019110.1 | Tubulin beta chain | 233.2 (M:233.2) | 15.1 | 50.5 | 4.6 | 4 | 35 |
| IMGA\|Medtr4g130860.1 | Leucine aminopeptidase 2, chloroplastic | 228.2 (M:228.2) | 9 | 59.6 | 8.6 | 3 | 36 |
| IMGA\|Medtr7g101640.1 | Cell division cycle protein 48 homolog | 225.6 (M:225.6) | 10 | 72.1 | 4.8 | 4 | 37 |
| PGKH2_ARATH | Phosphoglycerate kinase 2 | 218.6 (M:218.6) | 14.2 | 49.9 | 9.1 | 1 | 38 |
| IMGA\|Medtr5g064580.1 | 14-3-3 protein (Fragment) | 217.2 (M:217.2) | 16.5 | 29.2 | 4.5 | 3 | 39 |
| TBA_PRUDU | Tubulin alpha chain | 215.8 (M:215.8) | 10.9 | 49.5 | 4.8 | 3 | 40 |
| IMGA\|Medtr1g023120.1 | Beta-galactosidase | 195.1 (M:195.1) | 10.7 | 83.1 | 9.2 | 4 | 41 |
| IMGA\|Medtr8g106790.1 | Guanine nucleotide-binding protein subunit beta-like protein | 180.9 (M:180.9) | 20.9 | 35.7 | 7.8 | 3 | 42 |
| BIP3_MAIZE | Luminal-binding protein 3 precursor (BiP3) | 176.5 (M:176.5) | 3.9 | 73.1 | 5 | 1 | 43 |
| IMGA\|Medtr8g085980.1 | Alpha-tubulin | 176.4 (M:176.4) | 9.4 | 49.5 | 4.9 | 1 | 44 |
| IMGA\|Medtr1g098170.1 | 40S ribosomal protein S18 | 176.1 (M:176.1) | 18.4 | 17.6 | 11.3 | 3 | 45 |
| IMGA\|Medtr5g033090.1 | 60S ribosomal protein L27a-3 | 174.0 (M:174.0) | 21.3 | 18.5 | 11 | 3 | 46 |
| IMGA\|Medtr8g038210.1 | Annexin-like protein RJ4 | 162.8 (M:162.8) | 7.1 | 38.6 | 7.1 | 2 | 47 |
| IMGA\|Medtr4g107940.1 | Delta-1-pyrroline-5-carboxylate dehydrogenase 1 protein | 156.1 (M:156.1) | 6.1 | 61.3 | 6.4 | 3 | 48 |
| IMGA\|Medtr2g102180.1 | 97 kDa heat shock protein | 152.2 (M:152.2) | 2.8 | 95.2 | 5 | 2 | 49 |
| IMGA\|Medtr1g090130.1 | Chaperonin CPN60-2 | 149.5 (M:149.5) | 6.8 | 61.1 | 6.3 | 2 | 50 |
| IMGA\|AC146630_2.1 | 2-cys peroxiredoxin | 146.3 (M:146.3) | 19.6 | 29 | 6.1 | 2 | 51 |
| ATPAM_PEA | ATP synthase subunit alpha, mitochondrial | 145.9 (M:145.9) | 6.3 | 55 | 6 | 2 | 52 |
| IMGA\|Medtr7g076940.1 | 60S ribosomal protein L2 | 140.6 (M:140.6) | 12 | 28 | 11.5 | 2 | 53 |
| IMGA\|Medtr7g089120.2 | Tubulin beta chain | 139.6 (M:139.6) | 11.4 | 50.5 | 4.6 | 1 | 54 |
| IMGA\|Medtr3g095530.2 | Actin | 138.4 (M:138.4) | 14.7 | 40 | 5.6 | 3 | 55 |
| IMGA\|Medtr7g101580.1 | Cell division control protein 48 homolog E | 136.6 (M:136.6) | 9.5 | 26.8 | 10.3 | 2 | 56 |
| IMGA\|Medtr2g005690.1 | Heat shock protein 70 (Fragment) | 133.2 (M:133.2) | 6.8 | 75.7 | 5.1 | 3 | 57 |
| IMGA\|Medtr8g012330.3 | Ribosomal protein S8 | 132.4 (M:132.4) | 21.7 | 16 | 11.6 | 2 | 58 |
| IMGA\|Medtr3g093110.1 | Ribosomal protein L9 (Fragment) | 128.2 (M:128.2) | 15 | 21.7 | 9.9 | 2 | 59 |
| IMGA\|Medtr2g100560.1 | IAA-amino acid hydrolase ILR1-like 4 | 126.4 (M:126.4) | 7.8 | 48.8 | 5.6 | 2 | 60 |
| IMGA\|Medtr8g105340.1 | 40S ribosomal protein S2 | 125.8 (M:125.8) | 11.1 | 30.4 | 11 | 2 | 61 |
| ENO_ORYSJ | Enolase | 122.4 (M:122.4) | 12.1 | 47.9 | 5.3 | 1 | 62 |
| IMGA\|Medtr3g100500.1 | Aspartic proteinase nepenthesin-1 | 122.2 (M:122.2) | 4.1 | 46.7 | 9.6 | 2 | 63 |
| IMGA\|Medtr7g084800.1 | Glyceraldehyde-3-phosphate dehydrogenase A | 121.7 (M:121.7) | 9.4 | 43 | 9.3 | 2 | 64 |
| IMGA\|Medtr4g059680.1 | Beta-galactosidase | 120.3 (M:120.3) | 4.1 | 91.3 | 6.3 | 2 | 65 |
| IMGA\|Medtr3g118030.1 | 60S ribosomal protein L5 | 118.0 (M:118.0) | 8.2 | 37.5 | 9.6 | 2 | 66 |
| IMGA\|AC233663_14.1 | RuBisCO large subunit-binding protein subunit alpha | 115.2 (M:115.2) | 9.9 | 49.3 | 4.8 | 2 | 67 |
| IMGA\|Medtr1g106900.1 | 40S ribosomal protein S18 | 114.4 (M:114.4) | 18.4 | 17.6 | 11.3 | 1 | 68 |
| IMGA\|Medtr3g098430.1 | Calnexin homolog | 111.1 (M:111.1) | 5.5 | 61.7 | 4.6 | 2 | 69 |
| IMGA\|Medtr7g074570.3 | 2,3-bisphosphoglycerate-independent phosphoglycerate mutase | 109.1 (M:109.1) | 9.6 | 42.6 | 5.4 | 2 | 70 |
| IMGA\|Medtr3g104720.1 | Puromycin-sensitive aminopeptidase | 108.5 (M:108.5) | 3.8 | 98.9 | 5.4 | 2 | 71 |
| IMGA\|AC235488_11.1 | 40S ribosomal protein | 108.1 (M:108.1) | 6.1 | 32.8 | 4.7 | 2 | 72 |
| IMGA\|Medtr2g008050.1 | Actin | 105.8 (M:105.8) | 9.8 | 41.6 | 5.2 | 1 | 73 |
| IMGA\|Medtr4g131970.1 | RNA-binding protein 42 | 104.8 (M:104.8) | 7.1 | 45.7 | 6.3 | 2 | 74 |
| IMGA\|Medtr3g114400.1 | Subtilisin-like protease | 104.6 (M:104.6) | 14.6 | 17.8 | 4.9 | 1 | 75 |
| IMGA\|Medtr2g010020.1 | Heat shock 70 kDa protein, mitochondrial | 104.6 (M:104.6) | 4.1 | 72.4 | 5.4 | 1 | 76 |
| IMGA\|Medtr7g118060.1 | 60s acidic ribosomal protein P1 | 103.4 (M:103.4) | 44.2 | 11.4 | 4 | 2 | 77 |
| IMGA\|Medtr7g110310.1 | S-adenosylmethionine synthetase | 103.0 (M:103.0) | 7.6 | 43.3 | 5.5 | 2 | 78 |
| IMGA\|Medtr5g084930.1 | Protein disulfide isomerase family | 102.9 (M:102.9) | 6 | 47.4 | 5.3 | 2 | 79 |
| PSBB_DAUCA | Photosystem II CP47 chlorophyll apoprotein | 102.4 (M:102.4) | 6.7 | 55.9 | 6.4 | 2 | 80 |
| VATA_CITUN | Vacuolar ATP synthase catalytic subunit A (EC 3.6.3.14) | 100.6 (M:100.6) | 5 | 68.6 | 5.2 | 2 | 81 |

| | | | | | | |
|---|---|---|---|---|---|---|
| PSBO_PEA | Oxygen-evolving enhancer protein 1 | 99.5 (M:99.5) | 7.3 | 34.9 | 6.3 | 2 | 82 |
| IMGA\|Medtr3g005430.1 | 40S ribosomal protein S9 | 98.3 (M:98.3) | 12.7 | 22.9 | 10.7 | 2 | 83 |
| IMGA\|Medtr4g023630.1 | Aspartic proteinase Asp1 | 98.2 (M:98.2) | 8.6 | 45.3 | 9.6 | 2 | 84 |
| IMGA\|Medtr2g089860.1 | Subtilisin-like protease | 97.9 (M:97.9) | 3.5 | 81.8 | 8.7 | 2 | 85 |
| IMGA\|Medtr4g070080.1 | Glycine-rich RNA binding protein 1 | 97.0 (M:97.0) | 17.4 | 15.7 | 5.4 | 2 | 86 |
| CP18C_ARATH | Peptidyl-prolyl cis-trans isomerase CYP18-3 (EC 5.2.1.8) | 96.9 (M:96.9) | 8.7 | 18.4 | 9 | 2 | 87 |
| RUBB_SECCE | RuBisCO large subunit-binding protein subunit beta | 96.2 (M:96.2) | 7.8 | 53.4 | 4.7 | 2 | 88 |
| IMGA\|Medtr8g058480.1 | Zinc finger CCCH domain-containing protein 51 | 94.7 (M:94.7) | 7 | 39 | 4.7 | 1 | 89 |
| IMGA\|Medtr7g088680.1 | Nascent polypeptide-associated complex subunit alpha-like protein | 94.2 (M:94.2) | 8.4 | 22.1 | 4.2 | 1 | 90 |
| IMGA\|Medtr4g075290.1 | Peptidyl-prolyl cis-trans isomerase | 93.9 (M:93.9) | 20.3 | 18.2 | 9.5 | 2 | 91 |
| IMGA\|Medtr7g052690.1 | Early tobacco anther 1 | 92.7 (M:92.7) | 26.1 | 16.9 | 4.5 | 2 | 92 |
| IMGA\|Medtr3g108280.1 | 60S acidic ribosomal protein p0 | 92.6 (M:92.6) | 10.8 | 34.4 | 5.1 | 2 | 93 |
| IMGA\|Medtr4g115970.2 | Vacuolar proton-inorganic pyrophosphatase | 92.5 (M:92.5) | 5.3 | 75.6 | 4.9 | 2 | 94 |
| IMGA\|Medtr2g019780.1 | Auxin-binding protein ABP19a | 92.1 (M:92.1) | 17.5 | 22.7 | 9.8 | 2 | 95 |
| IMGA\|Medtr5g022940.1 | Aconitate hydratase (Fragment) | 91.5 (M:91.5) | 4 | 98.3 | 6.1 | 2 | 96 |
| IMGA\|Medtr8g104540.2 | Phosphoglucomutase | 91.4 (M:91.4) | 6.2 | 63.2 | 5.4 | 2 | 97 |
| IMGA\|Medtr3g008580.1 | Monocopper oxidase-like protein SKS1 | 89.5 (M:89.5) | 7.3 | 65.7 | 5.6 | 2 | 98 |
| IMGA\|Medtr1g035230.1 | Pyrophosphate--fructose 6-phosphate 1-phosphofructokinase | 88.1 (M:88.1) | 5.2 | 65.1 | 6.2 | 2 | 99 |
| MDHM_IMPCY | Malate dehydrogenase | 88.0 (M:88.0) | 100 | 3.1 | 7 | 2 | 100 |
| IMGA\|Medtr1g045410.1 | 60S ribosomal protein L4 | 87.7 (M:87.7) | 4.7 | 44.7 | 10.9 | 1 | 101 |
| IMGA\|Medtr2g095730.1 | Serine carboxypeptidase | 85.1 (M:85.1) | 7.1 | 57.3 | 5.6 | 2 | 102 |
| IMGA\|Medtr1g116120.1 | Transketolase 1 | 84.4 (M:84.4) | 4.9 | 79.7 | 6 | 2 | 103 |
| IMGA\|Medtr5g016590.1 | Proteasome subunit alpha type | 84.4 (M:84.4) | 6.4 | 27.3 | 7.4 | 1 | 104 |
| IMGA\|Medtr3g092900.1 | Caffeic acid O-methyltransferase (Fragment) | 84.1 (M:84.1) | 5.2 | 39.9 | 5.6 | 1 | 105 |
| IMGA\|Medtr8g092040.1 | Unknown Protein | 83.7 (M:83.7) | 7.4 | 27.7 | 8.8 | 1 | 106 |
| IMGA\|Medtr5g018940.1 | 40S ribosomal protein S4 | 83.6 (M:83.6) | 8.3 | 29.9 | 10.8 | 2 | 107 |
| RS281_ARATH | 40S ribosomal protein S28-1 OS=Arabidopsis thaliana | 83.2 (M:83.2) | 18.8 | 7.4 | 11.3 | 1 | 108 |
| IMGA\|Medtr6g021670.1 | 40S ribosomal protein S7-like protein | 83.1 (M:83.1) | 13.6 | 21.9 | 10.3 | 1 | 109 |
| IMGA\|AC235753_1.1 | Cysteine proteinase | 80.8 (M:80.8) | 10.5 | 40.3 | 6.1 | 2 | 110 |
| IMGA\|Medtr2g098010.1 | Proteasome subunit beta type | 80.0 (M:80.0) | 11.3 | 25.4 | 6.3 | 1 | 111 |
| IMGA\|Medtr2g014030.1 | 40S ribosomal protein S6 | 76.4 (M:76.4) | 6.5 | 28.2 | 11.5 | 1 | 112 |
| G3PB_TOBAC | Glyceraldehyde-3-phosphate dehydrogenase B | 75.8 (M:75.8) | 3.9 | 47.4 | 9.8 | 1 | 113 |
| IMGA\|Medtr7g081700.1 | Ras-like protein (Fragment) | 75.0 (M:75.0) | 13.4 | 22.5 | 4.8 | 2 | 114 |
| RL37A_GOSHI | 60S ribosomal protein L37a | 74.1 (M:74.1) | 17.4 | 10.2 | 11.3 | 1 | 115 |
| IMGA\|Medtr4g061140.1 | Cytosolic ascorbate peroxidase | 74.0 (M:74.0) | 7.2 | 27.1 | 5.5 | 1 | 116 |
| IMGA\|Medtr2g076960.2 | 14-3-3-like protein | 73.9 (M:73.9) | 9.1 | 30.1 | 4.6 | 1 | 117 |
| IMGA\|Medtr2g039250.1 | Nucleosome/chromatin assembly factor group (Fragment) | 73.4 (M:73.4) | 8.4 | 55.6 | 6.1 | 2 | 118 |
| IMGA\|Medtr6g009650.1 | Kunitz-type trypsin inhibitor-like 1 protein | 71.6 (M:71.6) | 16.2 | 22.5 | 4.8 | 1 | 119 |
| IMGA\|Medtr1g061630.2 | Pyruvate kinase | 71.2 (M:71.2) | 4.9 | 56 | 6.5 | 1 | 120 |
| IMGA\|Medtr1g116230.1 | Leukotriene-A4 hydrolase-like protein | 68.2 (M:68.2) | 2 | 68.6 | 5.1 | 1 | 121 |
| IMGA\|Medtr8g085440.1 | Glucan endo-1 3-beta-glucosidase 4 | 67.0 (M:67.0) | 22.4 | 12.3 | 9.1 | 1 | 122 |
| RS5_NICPL | 40S ribosomal protein S5 (Fragment) | 66.2 (M:66.2) | 9.7 | 17.1 | 11.1 | 1 | 123 |
| IMGA\|Medtr4g059400.1 | 60S ribosomal protein L12 | 62.1 (M:62.1) | 9 | 17.8 | 9.7 | 1 | 124 |
| RS271_ARATH | 40S ribosomal protein S27-1 | 60.9 (M:60.9) | 20.2 | 9.4 | 10.6 | 1 | 125 |
| IMGA\|Medtr2g039680.1 | Nucleosome assembly protein 1-like 1 | 60.4 (M:60.4) | 6.8 | 41.8 | 4.1 | 1 | 126 |
| IMGA\|Medtr2g005570.2 | Elongation factor 1-gamma | 59.2 (M:59.2) | 4.5 | 47.7 | 7.7 | 1 | 127 |
| IMGA\|Medtr1g094630.1 | 60S ribosomal protein L4 | 58.6 (M:58.6) | 1.7 | 130.9 | 6.5 | 1 | 128 |
| IMGA\|Medtr5g078200.1 | Glucan endo-1,3-beta-glucosidase 2 | 57.2 (M:57.2) | 3.6 | 69.2 | 4.7 | 1 | 129 |
| IMGA\|Medtr5g098060.1 | Fasciclin-like arabinogalactan protein 2 | 56.9 (M:56.9) | 6.9 | 27.8 | 5 | 1 | 130 |
| IMGA\|Medtr2g065470.3 | Glyceraldehyde-3-phosphate dehydrogenase | 56.7 (M:56.7) | 3.6 | 41.7 | 9.6 | 1 | 131 |
| IMGA\|Medtr3g113740.1 | DNA repair and recombination protein PIF1 | 56.0 (M:56.0) | 12.5 | 13.8 | 7.8 | 1 | 132 |
| UBIQ_AVESA | Ubiquitin | 55.3 (M:55.3) | 11.8 | 8.5 | 7.6 | 1 | 133 |
| SODC5_MAIZE | Superoxide dismutase [Cu-Zn] | 54.8 (M:54.8) | 6.6 | 15.1 | 5.6 | 1 | 134 |
| IMGA\|AC235665_13.2 | 40S ribosomal protein S3a | 54.1 (M:54.1) | 8.8 | 18.4 | 10.1 | 1 | 135 |
| IMGA\|Medtr7g080180.1 | Serine carboxypeptidase-like 20 | 54.0 (M:54.0) | 4 | 55.5 | 7.1 | 1 | 136 |
| EF1D1_ARATH | Elongation factor 1-delta 1 | 53.4 (M:53.4) | 10.4 | 25.1 | 4.3 | 1 | 137 |
| IMGA\|Medtr7g084150.1 | Ubiquinol-cytochrome c reductase iron-sulfur subunit | 53.3 (M:53.3) | 7 | 30.2 | 9.7 | 1 | 138 |
| IMGA\|Medtr4g124660.4 | Sucrose synthase | 53.3 (M:53.3) | 4.4 | 54.2 | 6 | 1 | 139 |
| RH6_ORYSJ | DEAD-box ATP-dependent RNA helicase 6 | 53.2 (M:53.2) | 5 | 56.7 | 9.6 | 1 | 140 |
| IMGA\|Medtr7g115070.1 | Unknown Protein | 52.6 (M:52.6) | 4.5 | 36.9 | 4.2 | 1 | 141 |
| IMGA\|Medtr3g086330.1 | T-complex protein 1 subunit epsilon | 52.3 (M:52.3) | 4.9 | 59.2 | 5.4 | 1 | 142 |
| IMGA\|Medtr3g084310.1 | Serine hydroxymethyltransferase | 52.1 (M:52.1) | 3.4 | 51.6 | 7 | 1 | 143 |
| IMGA\|Medtr4g024550.1 | 40S ribosomal protein S13 | 51.7 (M:51.7) | 8.7 | 15.8 | 10.8 | 1 | 144 |
| IMGA\|Medtr7g069390.1 | Proliferation-associated protein 2G4 | 51.1 (M:51.1) | 4.3 | 43.5 | 6.1 | 1 | 145 |
| ALFC3_ARATH | Probable fructose-bisphosphate aldolase 3 | 50.8 (M:50.8) | 7.2 | 42.3 | 9 | 1 | 146 |
| EF1B_ORYSJ | Elongation factor 1-beta | 49.9 (M:49.9) | 7.6 | 23.8 | 4.5 | 1 | 147 |
| IMGA\|Medtr5g082900.1 | Clathrin heavy chain | 49.8 (M:49.8) | 0.7 | 193.2 | 5.2 | 1 | 148 |
| IMGA\|Medtr4g005880.1 | Phosphoglycerate dehydrogenase | 49.7 (M:49.7) | 4 | 66.6 | 6.8 | 1 | 149 |
| IMGA\|Medtr2g063000.1 | Serine carboxypeptidase II-2 (Fragment) | 49.6 (M:49.6) | 4.2 | 53.4 | 5.6 | 1 | 150 |
| IMGA\|Medtr5g014960.1 | Branched-chain-amino-acid aminotransferase | 49.1 (M:49.1) | 5.9 | 44.2 | 7.5 | 1 | 151 |
| IMGA\|Medtr5g088660.1 | Elongation factor 1-beta 1 | 49.0 (M:49.0) | 10.8 | 24.2 | 4.5 | 1 | 152 |
| IMGA\|Medtr5g037960.1 | Unknown Protein | 48.6 (M:48.6) | 3.9 | 47.1 | 5 | 1 | 153 |
| IMGA\|Medtr6g055020.1 | ATP synthase subunit beta, chloroplastic | 48.5 (M:48.5) | 8.9 | 30.1 | 6.2 | 1 | 154 |
| CB24_PETSP | Chlorophyll a-b binding protein 25 | 48.4 (M:48.4) | 13.2 | 28.1 | 5.3 | 1 | 155 |
| IMGA\|Medtr3g088450.1 | Photosystem Q(B) protein | 48.3 (M:48.3) | 9.6 | 12.8 | 10.5 | 1 | 156 |
| IMGA\|Medtr8g027080.1 | Cytochrome b5 | 48.3 (M:48.3) | 16.9 | 15.6 | 4.7 | 1 | 157 |
| CPNA1_ARATH | Chaperonin 60 subunit alpha 1 | 48.2 (M:48.2) | 2.9 | 62 | 4.9 | 1 | 158 |
| IMGA\|Medtr5g088850.2 | Unknown Protein | 48.0 (M:48.0) | 14.3 | 12.2 | 4.3 | 1 | 159 |
| IF413_TOBAC | Eukaryotic initiation factor 4A-13 | 47.9 (M:47.9) | 4.5 | 40.2 | 5 | 1 | 160 |
| IMGA\|Medtr3g114850.1 | Plastocyanin | 47.8 (M:47.8) | 14.4 | 17.1 | 4.8 | 1 | 161 |
| IMGA\|Medtr7g083560.1 | Translocon-associated protein subunit beta | 47.8 (M:47.8) | 13.4 | 21 | 9.9 | 1 | 162 |
| IMGA\|Medtr4g039740.1 | Unknown Protein | 47.5 (M:47.5) | 2.5 | 40.2 | 10 | 1 | 163 |
| AB22B_ARATH | ABC transporter B family member | 47.4 (M:47.4) | 1.1 | 134.9 | 9.4 | 1 | 164 |
| IMGA\|Medtr3g092980.2 | Nascent polypeptide-associated complex alpha subunit | 46.8 (M:46.8) | 7.8 | 23.6 | 4.2 | 1 | 165 |

| | | | | | | | |
|---|---|---|---|---|---|---|---|
| IMGA\|Medtr2g060830.1 | Proteasome subunit alpha type | 46.7 (M:46.7) | 4.1 | 27.2 | 5.6 | 1 | 166 |
| IMGA\|Medtr4g074670.2 | Alanyl-tRNA synthetase | 46.6 (M:46.6) | 3.1 | 42.7 | 9.8 | 1 | 167 |
| RL7A2_ARATH | 60S ribosomal protein L7a-2 | 46.4 (M:46.4) | 4.7 | 29 | 10.7 | 1 | 168 |
| PSBP_PEA | Oxygen-evolving enhancer protein 2 | 46.4 (M:46.4) | 3.9 | 28 | 9.1 | 1 | 169 |
| IMGA\|Medtr4g112670.1 | Strictosidine synthase 1 | 46.1 (M:46.1) | 5.1 | 35.5 | 6.2 | 1 | 170 |
| IMGA\|Medtr4g095450.1 | Peroxidase | 46.0 (M:46.0) | 7.1 | 35.4 | 10.2 | 1 | 171 |
| IMGA\|Medtr7g110660.1 | Bifunctional aminoacyl-tRNA synthetase | 46.0 (M:46.0) | 4.9 | 60 | 6.1 | 1 | 172 |
| IMGA\|Medtr5g057990.1 | Alpha-D-xylosidase | 46.0 (M:46.0) | 1.8 | 102.9 | 6.3 | 1 | 173 |
| IMGA\|Medtr8g106020.1 | 40S ribosomal protein S16 | 45.9 (M:45.9) | 7.1 | 16.1 | 11 | 1 | 174 |
| IMGA\|Medtr6g071940.1 | Resistance protein (Fragment) | 45.8 (M:45.8) | 2.1 | 136.9 | 6.9 | 1 | 175 |
| IMGA\|Medtr4g132110.3 | Peroxidase | 45.6 (M:45.6) | 7.6 | 22.3 | 9.8 | 1 | 176 |
| IMGA\|Medtr8g086070.1 | Mitochondrial 2-oxoglutarate/malate carrier protein | 45.6 (M:45.6) | 4 | 32.2 | 0 | 1 | 177 |
| ALFC2_PEA | Fructose-bisphosphate aldolase 2 | 45.5 (M:45.5) | 8 | 37.8 | 5.4 | 1 | 178 |
| ADHX_PEA | Alcohol dehydrogenase class 3 (EC 1.1.1.1) | 45.4 (M:45.4) | 2.9 | 40.5 | 6.3 | 1 | 179 |
| RS172_ARATH | 40S ribosomal protein S17-2 | 45.4 (M:45.4) | 8.6 | 15.9 | 10.5 | 1 | 180 |
| IMGA\|Medtr1g045750.1 | 3-isopropylmalate dehydratase | 45.3 (M:45.3) | 5 | 55 | 7.1 | 1 | 181 |
| IMGA\|Medtr2g008160.3 | Peroxidase 43 | 45.3 (M:45.3) | 6.5 | 18.7 | 9.1 | 1 | 182 |
| SSG1_PEA | Granule-bound starch synthase 1 | 45.2 (M:45.2) | 3.5 | 66.3 | 6.7 | 1 | 183 |
| IMGA\|Medtr5g093530.1 | Leucine zipper protein | 44.9 (M:44.9) | 4 | 66.5 | 9 | 1 | 184 |
| IMGA\|Medtr3g092600.1 | 60S acidic ribosomal protein P1 | 44.5 (M:44.5) | 14.5 | 11.1 | 4.1 | 1 | 185 |
| G3PP1_ARATH | Glyceraldehyde 3-phosphate dehydrogenase | 44.1 (M:44.1) | 4.7 | 44.8 | 9.4 | 1 | 186 |
| H2A3_VOLCA | Histone H2A-III | 44.1 (M:44.1) | 7 | 13.5 | 10.7 | 1 | 187 |
| RGP3_ARATH | UDP-arabinopyranose mutase 3 | 44.0 (M:44.0) | 7.2 | 41.3 | 5.3 | 1 | 188 |
| IMGA\|Medtr1g012540.1 | N-acetyl-gamma-glutamyl-phosphate reductase | 43.8 (M:43.8) | 5.5 | 42.2 | 7.9 | 1 | 189 |
| VATB2_GOSHI | Vacuolar ATP synthase subunit B isoform 2 (EC 3.6.3.14) | 43.8 (M:43.8) | 5.7 | 43.2 | 4.9 | 1 | 190 |
| AI5L6_ARATH | ABSCISIC ACID-INSENSITIVE 5-like protein 6 | 43.8 (M:43.8) | 3.5 | 49.6 | 9.4 | 1 | 191 |
| IMGA\|Medtr2g005610.1 | Eukaryotic translation initiation factor 3 subunit C | 43.6 (M:43.6) | 1.4 | 110.9 | 5.6 | 1 | 192 |
| IMGA\|Medtr5g098420.1 | Fasciclin-like arabinogalactan protein 2 | 43.6 (M:43.6) | 4.8 | 45.1 | 5.9 | 1 | 193 |
| IMGA\|Medtr6g087990.1 | Peroxiredoxin | 43.0 (M:43.0) | 12.3 | 17.5 | 5.5 | 1 | 194 |
| IMGA\|Medtr2g014220.1 | Ribosomal protein L15 | 42.5 (M:42.5) | 4 | 29.7 | 12.1 | 1 | 195 |
| IMGA\|Medtr5g086090.1 | Receptor-like protein kinase | 42.5 (M:42.5) | 1.6 | 68.6 | 5.4 | 1 | 196 |
| ZB14_BRAJU | 14 kDa zinc-binding protein (Protein kinase C inhibitor) | 42.5 (M:42.5) | 15 | 12.6 | 6.6 | 1 | 197 |
| IMGA\|Medtr8g093770.1 | 40S ribosomal protein S12 | 42.2 (M:42.2) | 11.9 | 15.2 | 5.4 | 1 | 198 |
| SAR2_SOLLC | GTP-binding protein | 41.6 (M:41.6) | 6.7 | 21.9 | 6.5 | 1 | 199 |
| RS14_LUPLU | 40S ribosomal protein S14 | 41.5 (M:41.5) | 8.7 | 16.3 | 11.5 | 1 | 200 |
| RBL_VITSX | Ribulose bisphosphate carboxylase large chain | 41.4 (M:41.4) | 18.2 | 6.1 | 6.5 | 1 | 201 |
| PSA5A_ARATH | Proteasome subunit alpha type-5 | 41.2 (M:41.2) | 8 | 25.9 | 4.6 | 1 | 202 |
| MDAR_PEA | Monodehydroascorbate reductase | 41.0 (M:41.0) | 3 | 47.3 | 5.7 | 1 | 203 |
| TLP6_ORYSJ | Tubby-like F-box protein 6 | 40.9 (M:40.9) | 4.5 | 44.9 | 10.3 | 1 | 204 |
| PSBD_PSEAK | Photosystem II D2 protein | 40.8 (M:40.8) | 2.6 | 39.3 | 6.1 | 1 | 205 |
| IMGA\|Medtr1g018840.3 | Cysteine proteinase 3 (Fragment) | 40.7 (M:40.7) | 7.4 | 33.3 | 4.9 | 1 | 206 |
| FKB15_VICFA | FK506-binding protein 2 | 40.2 (M:40.2) | 8.6 | 16.2 | 7.6 | 1 | 207 |
| IMGA\|Medtr6g077790.1 | Unknown Protein | 40.0 (M:40.0) | 19 | 6.9 | 11.9 | 1 | 208 |
| IMGA\|Medtr7g116650.1 | Wall-associated receptor kinase-like 8 | 40.0 (M:40.0) | 2.2 | 60.3 | 8.9 | 1 | 209 |
| IMGA\|AC233657_22.1 | Chlorophyll a-b binding protein | 40.0 (M:40.0) | 9.7 | 28.8 | 6.5 | 1 | 210 |
| IMGA\|Medtr5g033920.1 | ATP-citrate synthase | 40.0 (M:40.0) | 5.7 | 46.6 | 5.5 | 1 | 211 |
| NDK1_SOYBN | Nucleoside diphosphate kinase 1 (EC 2.7.4.6) | 39.7 (M:39.7) | 10.1 | 16.4 | 5.9 | 1 | 212 |
| FB254_ARATH | F-box protein | 39.6 (M:39.6) | 5.5 | 53 | 9.3 | 1 | 213 |
| IMGA\|Medtr5g034820.1 | Threonyl-tRNA synthetase | 39.5 (M:39.5) | 3.2 | 82 | 6.6 | 1 | 214 |
| IMGA\|AC233659_13.1 | Monothiol glutaredoxin-S17 | 39.3 (M:39.3) | 5.9 | 53.9 | 5.1 | 1 | 215 |
| IMGA\|Medtr6g005550.2 | DEAD-box ATP-dependent RNA helicase 46 | 39.1 (M:39.1) | 4 | 66.4 | 10.1 | 1 | 216 |
| IMGA\|Medtr2g029800.3 | Peroxidase | 38.8 (M:38.8) | 6.2 | 15.6 | 5.8 | 1 | 217 |
| Y5600_ARATH | BTB/POZ domain-containing protein | 38.6 (M:38.6) | 2.2 | 56.9 | 6.1 | 1 | 218 |
| FH7_ARATH | Formin-like protein 7 | 38.4 (M:38.4) | 1.4 | 98.5 | 9.2 | 1 | 219 |
| IMGA\|Medtr1g095830.2 | ATP synthase subunit d, mitochondrial | 38.4 (M:38.4) | 9.7 | 16.8 | 5.5 | 1 | 220 |
| STR7_ARATH | Rhodanese-like domain-containing protein 7 | 38.4 (M:38.4) | 5.3 | 52.6 | 6.4 | 1 | 221 |
| IMGA\|Medtr1g088450.1 | 60S ribosomal protein L22-like | 38.3 (M:38.3) | 10.9 | 13.6 | 10 | 1 | 222 |
| IMGA\|AC235488_9.1 | 60S ribosomal protein L21 | 38.0 (M:38.0) | 6.1 | 18.7 | 11 | 1 | 223 |
| IMGA\|Medtr5g098310.2 | Acetyl-CoA acetyltransferase | 37.7 (M:37.7) | 6.3 | 39 | 6.2 | 1 | 224 |
| IMGA\|Medtr5g072480.2 | Ubiquitin-activating enzyme E1 | 37.7 (M:37.7) | 0.9 | 132 | 5.9 | 1 | 225 |
| IMGA\|Medtr4g125200.1 | Regulatory protein CII | 37.7 (M:37.7) | 12.9 | 25.4 | 9.5 | 1 | 226 |
| IMGA\|Medtr8g039540.1 | Aspartic proteinase nepenthesin-1 | 37.6 (M:37.6) | 6.7 | 46.8 | 10.5 | 1 | 227 |
| RL373_ARATH | 60S ribosomal protein L37-3 | 37.6 (M:37.6) | 11.6 | 10.8 | 12.8 | 1 | 228 |
| IMGA\|Medtr5g037650.1 | TIR-NBS disease resistance-like protein | 37.3 (M:37.3) | 8 | 31.8 | 9.7 | 1 | 229 |
| IMGA\|Medtr6g071090.1 | Translationally-controlled tumor protein homolog | 37.3 (M:37.3) | 5.6 | 18.2 | 4.6 | 1 | 230 |
| IMGA\|Medtr4g064750.3 | cDNA clone 002-143-C11 full insert sequence | 37.3 (M:37.3) | 15.5 | 20.9 | 10.4 | 1 | 231 |
| SPD2_HYONI | Spermidine synthase 2 | 37.1 (M:37.1) | 4.2 | 33.9 | 4.9 | 1 | 232 |
| IMGA\|Medtr6g032870.1 | T-complex protein 1 subunit theta | 37.1 (M:37.1) | 3.1 | 55.7 | 5 | 1 | 233 |
| IMGA\|Medtr4g093980.1 | Rab-GDP dissociation inhibitor | 37.0 (M:37.0) | 5.2 | 49.4 | 4.9 | 1 | 234 |
| IMGA\|Medtr2g028440.1 | Elongation factor G, mitochondrial | 36.8 (M:36.8) | 2.7 | 79.9 | 7.4 | 1 | 235 |
| IMGA\|Medtr5g087870.3 | Protein argonaute 4A | 36.8 (M:36.8) | 1.3 | 101.3 | 9.8 | 1 | 236 |
| IMGA\|Medtr1g100680.1 | Methionine synthase | 36.7 (M:36.7) | 2 | 85.1 | 6.9 | 1 | 237 |
| PSBO1_ARATH | Oxygen-evolving enhancer protein 1-1 | 36.6 (M:36.6) | 8.7 | 35.1 | 5.4 | 1 | 238 |
| PROF_PRUPE | Profilin | 36.5 (M:36.5) | 7.6 | 14 | 4.6 | 1 | 239 |
| TPS6_ARATH | Alpha,alpha-trehalose-phosphate synthase | 36.4 (M:36.4) | 2.7 | 97.6 | 5.9 | 1 | 240 |
| IMGA\|Medtr7g077970.1 | F-box/kelch-repeat protein | 36.3 (M:36.3) | 5 | 52.4 | 5 | 1 | 241 |
| CAF1J_ARATH | Probable CCR4-associated factor 1 homolog | 36.3 (M:36.3) | 2.2 | 31.5 | 4.8 | 1 | 242 |
| IMGA\|Medtr7g113470.1 | T-complex protein 1 subunit beta | 36.0 (M:36.0) | 4.7 | 56.9 | 5.3 | 1 | 243 |
| IMGA\|Medtr4g132270.2 | Lactoylglutathione lyase | 36.0 (M:36.0) | 4.5 | 27 | 4.8 | 1 | 244 |
| IMGA\|Medtr1g075790.1 | Os12g0236050 protein (Fragment) | 35.9 (M:35.9) | 1.9 | 111 | 5 | 1 | 245 |
| FL3H_ARATH | Naringenin,2-oxoglutarate 3-dioxygenase (EC 1.14.11.9) | 35.8 (M:35.8) | 5.3 | 40.3 | 5.2 | 1 | 246 |
| Y1215_ARATH | WEB family protein | 35.8 (M:35.8) | 2.2 | 62.6 | 5.2 | 1 | 247 |
| UGPA_PYRPY | UTP--glucose-1-phosphate uridylyltransferase (EC 2.7.7.9) | 35.8 (M:35.8) | 1.9 | 51.8 | 6 | 1 | 248 |
| IMGA\|Medtr2g036810.1 | cDNA clone J013170O13 full insert sequence | 35.7 (M:35.7) | 7.5 | 35.8 | 5.8 | 1 | 249 |

| | | | | | | | |
|---|---|---|---|---|---|---|---|
| IMGA\|Medtr7g111650.1 | ATP-binding domain-containing protein 4 | 35.5 (M:35.5) | 1.9 | 97.7 | 5.8 | 1 | 250 |
| ACCC1_POPTR | Biotin carboxylase 1 | 35.4 (M:35.4) | 3.4 | 57.7 | 7.1 | 1 | 251 |
| HIBC8_ARATH | 3-hydroxyisobutyryl-CoA hydrolase-like protein | 35.4 (M:35.4) | 5.9 | 43.2 | 5.3 | 1 | 252 |
| NU5C_AMBTC | NAD(P)H-quinone oxidoreductase subunit 5 | 35.4 (M:35.4) | 2.9 | 83.6 | 8.4 | 1 | 253 |
| IMGA\|Medtr6g071550.1 | Disease resistance protein | 35.3 (M:35.3) | 1.3 | 189.7 | 6.3 | 1 | 254 |
| PROF6_HEVBR | Profilin-6 | 35.2 (M:35.2) | 14.5 | 14 | 4.8 | 1 | 255 |
| IMGA\|Medtr4g070140.1 | Glycine-rich RNA binding protein 1 | 35.2 (M:35.2) | 11.1 | 18.4 | 5.1 | 1 | 256 |
| IMGA\|Medtr3g070810.1 | CTD small phosphatase-like protein 2 | 35.2 (M:35.2) | 8 | 31.5 | 4.4 | 1 | 257 |
| IMGA\|Medtr8g011440.1 | Receptor-like protein kinase (Fragment) | 35.1 (M:35.1) | 1.5 | 133.9 | 5.1 | 1 | 258 |
| IMGA\|Medtr1g083400.1 | Pre-mRNA-splicing factor SF2 | 35.0 (M:35.0) | 10.8 | 38.1 | 10.2 | 1 | 259 |
