# Supplementary material for: Regulation of Carbon Partitioning in the Seed of the Model Legume Medicago truncatula and Medicago orbicularis: A Comparative Approach
Source: Front Plant Sci. 2017 Dec 12;8:2070. doi: 10.3389/fpls.2017.02070 (PMC5733034; doi:10.3389/fpls.2017.02070)
Supplement: Supplementary file 4 [file Image_1.PDF]

Supplementary Fig. S1A Phylogram for GLABRA2

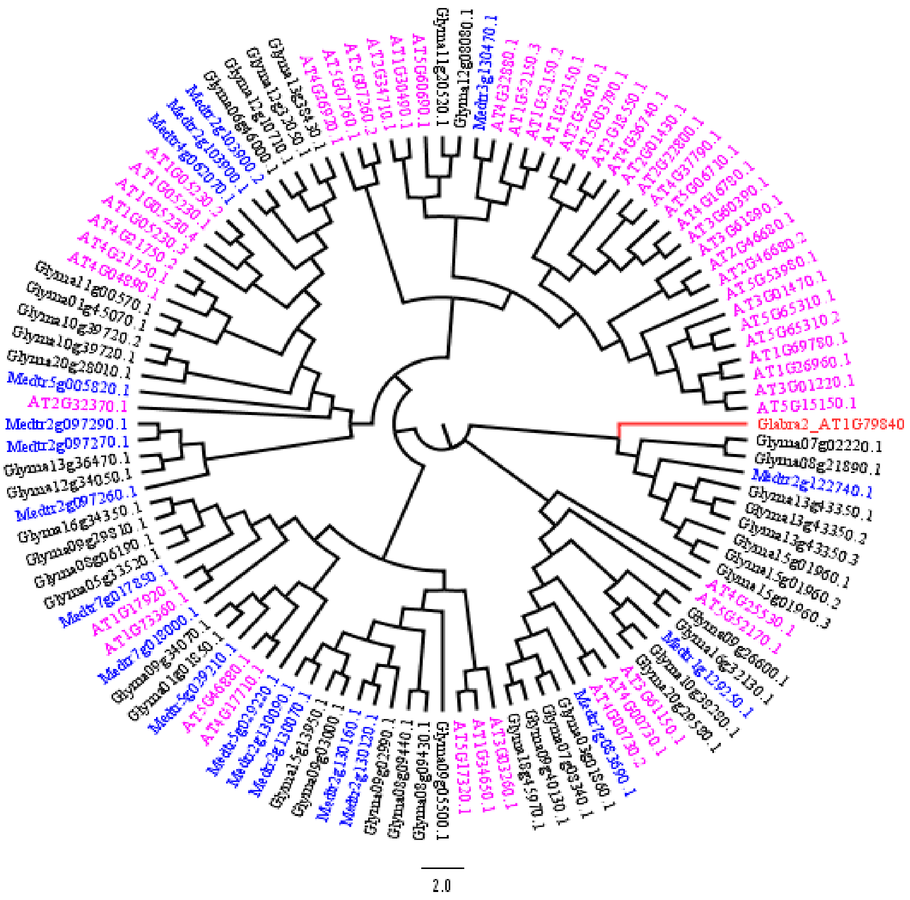

Supplementary Fig. S1b Sequence alignment of qPCR product for *MtGLABRA2* from *M. truncatula* and *M. orbicularis*

```
                     *        20         *        40         *        60         *        80         *       100         *
MtGL2     : CAACCATTGCTTTTGCTCCTTAAAAAACACAATACCCTTCTTTCTTCACCAACGTTGTCAATGTGTGCAGACATGTCTAATAATAATCCTCCTTCTACTTCTAAAGCCAAAGACTTC
MoSeqGL2  : -----------------------------------------------------------AATGTGTACAGACATGTCTAATAATAATCCTCCTTCTACTTCTAAAGCTAAAGACTTC
                                                                       AATGTGT CAGACATGTCTAATAATAATCCTCCTTCTACTTCTAAAGC AAAGACTTC

            120         *       140         *       160         *       180         *       200         *       220         *
MtGL2     : TTTCCCTCTCCAGCTCTCTCTCTTAGCCTAGCGGGGATATTTCGGCATGGTGGCGGTGCGGCGGCGGAGGGTGAAGGAGGGAGTATTAGTAACATGGAGGTGGAAGAAGGGGAGGAA
MoSeqGL2  : TTTCCCTCTCCAGCTCTCTCTCTTAGCCTTGCGGGGATTTTTCGGCATGGCGTTGCGGCGGCGGTGGATGGTGAAGGAGGGAGTATTAGTAATATGGAGGTGGAAGAAGGGGATGAA
            TTTCCCTCTCCAGCTCTCTCTCTTAGCCT GCGGGGAT TTTCGGCATGG G  G  GCGGCGG GGA GGTGAAGGAGGGAGTATTAGTAA ATGGAGGTGGAAGAAGGGGA GAA

               240         *       260         *       280         *       300         *       320         *       340         *
MtGL2     : GGGAGTACAATAGGAG-----------------------------------------------------------------------------------------------------
MoSeqGL2  : GGTAGTACAATAGGAG-----------------------------------------------------------------------------------------------------
            GG AGTACAATAGGAG
```
